# Supplementary figures and images for: Treatment preferences among people at risk of developing tuberculosis: A discrete choice experiment
Source: PLOS Glob Public Health. 2024 Jul 19;4(7):e0002804. doi: 10.1371/journal.pgph.0002804 (PMC11259259; doi:10.1371/journal.pgph.0002804)

**Supplemental Figure S1: Trace plots of Markov chain Monte Carlo draws from model
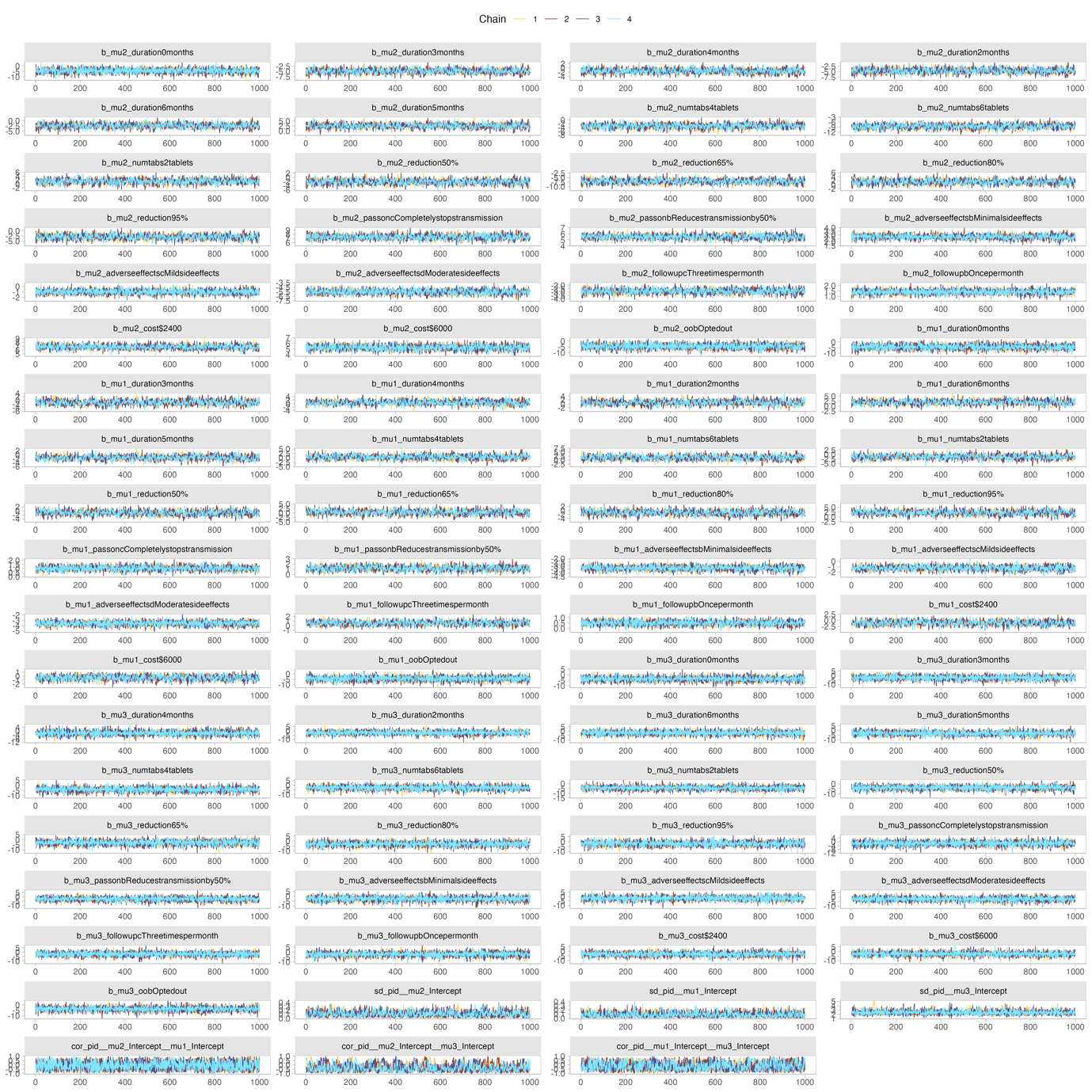
**

Supplement: S1 Fig — (DOCX) [file pgph.0002804.s002.docx]
