# Supplementary material for: Treatment preferences among people at risk of developing tuberculosis: A discrete choice experiment
Source: PLOS Glob Public Health. 2024 Jul 19;4(7):e0002804. doi: 10.1371/journal.pgph.0002804 (PMC11259259; doi:10.1371/journal.pgph.0002804)
Supplement: S1 Table — (DOCX) [file pgph.0002804.s003.docx]

**Supplemental table S1: Priors for regression model**

| **Prior** | **Class** | **Coefficient** | **Group** | **Distributional parameter** |
| --- | --- | --- | --- | --- |
| normal(0, 3) | b |  |  | mu1 |
| normal(0, 3) | b | mu1 |  |  |
| normal(0, 3) | b | mu1 |  |  |
| normal(0, 3) | b | mu1 |  |  |
| normal(0, 3) | b | cost$2400 |  | mu1 |
| normal(0, 3) | b | cost$6000 |  | mu1 |
| normal(0, 3) | b | duration0months | | mu1 |
| normal(0, 3) | b | duration2months | | mu1 |
| normal(0, 3) | b | duration3months | | mu1 |
| normal(0, 3) | b | duration4months | | mu1 |
| normal(0, 3) | b | duration5months | | mu1 |
| normal(0, 3) | b | duration6months | | mu1 |
| normal(0, 3) | b | followupbOncepermonth | | mu1 |
| normal(0, 3) | b | mu1 |  |  |
| normal(0, 3) | b | numtabs2tablets | | mu1 |
| normal(0, 3) | b | numtabs4tablets | | mu1 |
| normal(0, 3) | b | numtabs6tablets | | mu1 |
| normal(0, 3) | b | oobOptedout | | mu1 |
| normal(0, 3) | b | mu1 |  |  |
| normal(0, 3) | b | mu1 |  |  |
| normal(0, 3) | b | reduction50% | | mu1 |
| normal(0, 3) | b | reduction65% | | mu1 |
| normal(0, 3) | b | reduction80% | | mu1 |
| normal(0, 3) | b | reduction95% | | mu1 |
| normal(0, 3) | b |  |  | mu2 |
| normal(0, 3) | b | mu2 |  |  |
| normal(0, 3) | b | mu2 |  |  |
| normal(0, 3) | b | mu2 |  |  |
| normal(0, 3) | b | cost$2400 |  | mu2 |
| normal(0, 3) | b | cost$6000 |  | mu2 |
| normal(0, 3) | b | duration0months | | mu2 |
| normal(0, 3) | b | duration2months | | mu2 |
| normal(0, 3) | b | duration3months | | mu2 |
| normal(0, 3) | b | duration4months | | mu2 |
| normal(0, 3) | b | duration5months | | mu2 |
| normal(0, 3) | b | duration6months | | mu2 |
| normal(0, 3) | b | followupbOncepermonth | | mu2 |
| normal(0, 3) | b | mu2 |  |  |
| normal(0, 3) | b | numtabs2tablets | | mu2 |
| normal(0, 3) | b | numtabs4tablets | | mu2 |
| normal(0, 3) | b | numtabs6tablets | | mu2 |
| normal(0, 3) | b | oobOptedout | | mu2 |
| normal(0, 3) | b | mu2 |  |  |
| normal(0, 3) | b | mu2 |  |  |
| normal(0, 3) | b | reduction50% | | mu2 |
| normal(0, 3) | b | reduction65% | | mu2 |
| normal(0, 3) | b | reduction80% | | mu2 |
| normal(0, 3) | b | reduction95% | | mu2 |
| normal(0, 3) | b |  |  | mu3 |
| normal(0, 3) | b | mu3 |  |  |
| normal(0, 3) | b | mu3 |  |  |
| normal(0, 3) | b | mu3 |  |  |
| normal(0, 3) | b | cost$2400 |  | mu3 |
| normal(0, 3) | b | cost$6000 |  | mu3 |
| normal(0, 3) | b | duration0months | | mu3 |
| normal(0, 3) | b | duration2months | | mu3 |
| normal(0, 3) | b | duration3months | | mu3 |
| normal(0, 3) | b | duration4months | | mu3 |
| normal(0, 3) | b | duration5months | | mu3 |
| normal(0, 3) | b | duration6months | | mu3 |
| normal(0, 3) | b | followupbOncepermonth | | mu3 |
| normal(0, 3) | b | mu3 |  |  |
| normal(0, 3) | b | numtabs2tablets | | mu3 |
| normal(0, 3) | b | numtabs4tablets | | mu3 |
| normal(0, 3) | b | numtabs6tablets | | mu3 |
| normal(0, 3) | b | oobOptedout | | mu3 |
| normal(0, 3) | b | mu3 |  |  |
| normal(0, 3) | b | mu3 |  |  |
| normal(0, 3) | b | reduction50% | | mu3 |
| normal(0, 3) | b | reduction65% | | mu3 |
| normal(0, 3) | b | reduction80% | | mu3 |
| normal(0, 3) | b | reduction95% | | mu3 |
| lkj_corr_cholesky(1) | L |  |  |  |
| lkj_corr_cholesky(1) | L |  | pid |  |
| exponential(1) | sd |  |  | mu1 |
| exponential(1) | sd |  |  | mu2 |
| exponential(1) | sd |  |  | mu3 |
| exponential(1) | sd |  | pid | mu1 |
| exponential(1) | sd | Intercept | pid | mu1 |
| exponential(1) | sd |  | pid | mu2 |
| exponential(1) | sd | Intercept | pid | mu2 |
| exponential(1) | sd |  | pid | mu3 |
| exponential(1) | sd | Intercept | pid | mu3 |
