# Supplementary material for: Treatment preferences among people at risk of developing tuberculosis: A discrete choice experiment
Source: PLOS Glob Public Health. 2024 Jul 19;4(7):e0002804. doi: 10.1371/journal.pgph.0002804 (PMC11259259; doi:10.1371/journal.pgph.0002804)
Supplement: S2 Table — (DOCX) [file pgph.0002804.s004.docx]

**Supplemental Table S2: Summary of posterior draws**

| **Variable** | **Mean** | **Median** | **SD** | **q5** | **q95** | **Rhat** | **ESS Bulk** | **ESS Tail** |
| --- | --- | --- | --- | --- | --- | --- | --- | --- |
| b_mu2_duration0months | -4.43 | -4.41 | 2.29 | -8.27 | -0.67 | 1 | 4,150.34 | 2,801.23 |
| b_mu2_duration3months | -4.79 | -4.81 | 1.18 | -6.68 | -2.85 | 1 | 1,230.24 | 1,913.58 |
| b_mu2_duration4months | -1.43 | -1.44 | 1.18 | -3.34 | 0.52 | 1 | 1,284.54 | 1,901.96 |
| b_mu2_duration2months | -4.56 | -4.57 | 1.17 | -6.46 | -2.61 | 1 | 1,208.28 | 1,829.61 |
| b_mu2_duration6months | -2.33 | -2.35 | 1.17 | -4.24 | -0.42 | 1 | 1,215.05 | 1,783.99 |
| b_mu2_duration5months | 2.59 | 2.57 | 1.2 | 0.64 | 4.57 | 1 | 1,231.14 | 1,894.20 |
| b_mu2_numtabs4tablets | -3.22 | -3.18 | 1.29 | -5.37 | -1.14 | 1 | 1,382.49 | 2,080.05 |
| b_mu2_numtabs6tablets | -8.52 | -8.48 | 1.42 | -10.88 | -6.2 | 1 | 1,447.88 | 2,074.23 |
| b_mu2_numtabs2tablets | 1.37 | 1.39 | 1.31 | -0.81 | 3.51 | 1 | 1,389.74 | 2,164.85 |
| b_mu2_reduction50% | -2.13 | -2.15 | 1.24 | -4.16 | -0.07 | 1 | 1,372.53 | 2,085.84 |
| b_mu2_reduction65% | -7 | -6.98 | 1.33 | -9.18 | -4.83 | 1 | 1,364.06 | 2,078.14 |
| b_mu2_reduction80% | 1.44 | 1.41 | 1.25 | -0.57 | 3.54 | 1 | 1,369.67 | 2,129.88 |
| b_mu2_reduction95% | -2.93 | -2.95 | 1.27 | -5.03 | -0.83 | 1 | 1,388.55 | 2,201.80 |
| b_mu2_passoncCompletelystopstransmission | 7.38 | 7.4 | 0.6 | 6.39 | 8.39 | 1 | 1,716.54 | 2,117.43 |
| b_mu2_passonbReducestransmissionby50% | 5.5 | 5.49 | 0.39 | 4.86 | 6.14 | 1 | 1,710.28 | 2,453.08 |
| b_mu2_adverseeffectsbMinimalsideeffects | 2.73 | 2.73 | 0.32 | 2.22 | 3.27 | 1 | 2,419.77 | 2,733.04 |
| b_mu2_adverseeffectscMildsideeffects | -0.99 | -0.98 | 0.44 | -1.7 | -0.26 | 1 | 3,923.01 | 2,802.47 |
| b_mu2_adverseeffectsdModeratesideeffects | -5.57 | -5.58 | 0.55 | -6.48 | -4.67 | 1 | 1,917.81 | 2,799.14 |
| b_mu2_followupcThreetimespermonth | -2.87 | -2.87 | 0.38 | -3.49 | -2.27 | 1 | 1,652.29 | 2,335.37 |
| b_mu2_followupbOncepermonth | 1.43 | 1.43 | 0.22 | 1.06 | 1.79 | 1 | 5,829.64 | 2,921.81 |
| b_mu2_cost$2400 | 6.87 | 6.86 | 0.61 | 5.88 | 7.87 | 1 | 1,957.70 | 2,167.01 |
| b_mu2_cost$6000 | 5.29 | 5.29 | 0.46 | 4.53 | 6.06 | 1 | 2,575.36 | 2,781.52 |
| b_mu2_oobOptedout | -4.47 | -4.45 | 2.27 | -8.26 | -0.71 | 1 | 4,656.58 | 3,464.68 |
| b_mu1_duration0months | -4.4 | -4.39 | 2.3 | -8.24 | -0.63 | 1 | 4,683.45 | 3,048.01 |
| b_mu1_duration3months | -1.17 | -1.15 | 1.38 | -3.41 | 1.16 | 1 | 1,194.18 | 1,837.97 |
| b_mu1_duration4months | 0.59 | 0.62 | 1.28 | -1.5 | 2.69 | 1.01 | 1,346.14 | 1,920.86 |
| b_mu1_duration2months | 0.64 | 0.64 | 1.21 | -1.37 | 2.62 | 1.01 | 1,202.94 | 1,678.67 |
| b_mu1_duration6months | 1.96 | 1.97 | 1.17 | 0.06 | 3.9 | 1.01 | 1,260.48 | 1,891.68 |
| b_mu1_duration5months | -1.49 | -1.46 | 1.17 | -3.42 | 0.4 | 1.01 | 1,255.67 | 1,848.31 |
| b_mu1_numtabs4tablets | 0.42 | 0.42 | 1.3 | -1.72 | 2.53 | 1 | 1,384.39 | 2,272.63 |
| b_mu1_numtabs6tablets | 1.59 | 1.58 | 1.44 | -0.77 | 3.96 | 1 | 1,337.91 | 2,139.02 |
| b_mu1_numtabs2tablets | -1.36 | -1.35 | 1.29 | -3.44 | 0.7 | 1 | 1,396.14 | 1,939.41 |
| b_mu1_reduction50% | -0.93 | -0.95 | 1.24 | -2.92 | 1.2 | 1 | 1,233.19 | 1,846.01 |
| b_mu1_reduction65% | 0.34 | 0.32 | 1.37 | -1.85 | 2.57 | 1 | 1,317.77 | 1,944.62 |
| b_mu1_reduction80% | -1.13 | -1.17 | 1.26 | -3.14 | 1.02 | 1 | 1,213.44 | 1,870.58 |
| b_mu1_reduction95% | 2.21 | 2.21 | 1.28 | 0.11 | 4.39 | 1 | 1,328.92 | 1,961.30 |
| b_mu1_passoncCompletelystopstransmission | 1.03 | 1.03 | 0.29 | 0.56 | 1.52 | 1 | 3,622.89 | 2,812.80 |
| b_mu1_passonbReducestransmissionby50% | 1.21 | 1.22 | 0.48 | 0.42 | 1.99 | 1 | 1,497.59 | 2,703.50 |
| b_mu1_adverseeffectsbMinimalsideeffects | -3.33 | -3.32 | 0.34 | -3.89 | -2.77 | 1 | 2,514.16 | 2,394.11 |
| b_mu1_adverseeffectscMildsideeffects | -1.22 | -1.21 | 0.44 | -1.97 | -0.53 | 1 | 2,214.58 | 2,457.70 |
| b_mu1_adverseeffectsdModeratesideeffects | -3.58 | -3.57 | 0.46 | -4.37 | -2.84 | 1 | 3,162.82 | 3,002.72 |
| b_mu1_followupcThreetimespermonth | 0.55 | 0.56 | 0.5 | -0.26 | 1.37 | 1 | 1,503.53 | 2,306.64 |
| b_mu1_followupbOncepermonth | 0.5 | 0.5 | 0.23 | 0.13 | 0.87 | 1 | 3,222.76 | 3,268.81 |
| b_mu1_cost$2400 | -1 | -0.98 | 1 | -2.68 | 0.61 | 1 | 1,315.12 | 1,812.33 |
| b_mu1_cost$6000 | -0.42 | -0.41 | 0.63 | -1.49 | 0.61 | 1 | 1,606.72 | 2,217.55 |
| b_mu1_oobOptedout | -4.43 | -4.39 | 2.34 | -8.29 | -0.63 | 1 | 4,184.60 | 2,646.93 |
| b_mu3_duration0months | -3.75 | -3.74 | 2.13 | -7.18 | -0.22 | 1 | 4,831.33 | 3,317.12 |
| b_mu3_duration3months | -2.37 | -2.32 | 2.51 | -6.53 | 1.67 | 1 | 6,183.24 | 3,533.54 |
| b_mu3_duration4months | -2.2 | -2.21 | 2.65 | -6.53 | 2.21 | 1 | 7,067.88 | 3,191.69 |
| b_mu3_duration2months | -2.1 | -2.05 | 2.44 | -6.15 | 1.74 | 1 | 6,513.50 | 3,242.37 |
| b_mu3_duration6months | -1.89 | -1.82 | 2.56 | -6.13 | 2.21 | 1 | 7,157.01 | 3,247.82 |
| b_mu3_duration5months | -3.11 | -3.09 | 2.43 | -7.07 | 0.8 | 1 | 6,325.47 | 3,084.55 |
| b_mu3_numtabs4tablets | -4.08 | -4.07 | 2.31 | -7.98 | -0.33 | 1 | 4,233.79 | 3,300.65 |
| b_mu3_numtabs6tablets | -2.87 | -2.84 | 2.49 | -6.94 | 1.17 | 1 | 5,798.60 | 3,429.90 |
| b_mu3_numtabs2tablets | -4.56 | -4.52 | 2.27 | -8.29 | -0.82 | 1 | 5,886.46 | 3,191.53 |
| b_mu3_reduction50% | -3.79 | -3.75 | 2.21 | -7.52 | -0.27 | 1 | 5,932.15 | 3,523.90 |
| b_mu3_reduction65% | -3 | -2.98 | 2.49 | -7.25 | 0.95 | 1 | 5,945.48 | 3,165.15 |
| b_mu3_reduction80% | -2.86 | -2.85 | 2.39 | -6.86 | 1.03 | 1 | 6,180.78 | 3,264.50 |
| b_mu3_reduction95% | -1.96 | -1.95 | 2.46 | -6 | 1.98 | 1 | 6,144.51 | 3,089.57 |
| b_mu3_passoncCompletelystopstransmission | -1.72 | -1.66 | 2.55 | -6.05 | 2.34 | 1 | 6,735.52 | 3,410.49 |
| b_mu3_passonbReducestransmissionby50% | -2.51 | -2.45 | 2.45 | -6.67 | 1.43 | 1 | 5,899.07 | 3,187.29 |
| b_mu3_adverseeffectsbMinimalsideeffects | -2.51 | -2.43 | 2.44 | -6.63 | 1.44 | 1 | 6,135.16 | 3,103.07 |
| b_mu3_adverseeffectscMildsideeffects | -1.88 | -1.82 | 2.56 | -6.14 | 2.25 | 1 | 7,497.69 | 3,243.66 |
| b_mu3_adverseeffectsdModeratesideeffects | -2.03 | -1.99 | 2.49 | -6.13 | 1.98 | 1 | 6,213.11 | 2,937.52 |
| b_mu3_followupcThreetimespermonth | -2.01 | -1.94 | 2.52 | -6.2 | 1.98 | 1 | 6,138.19 | 3,345.07 |
| b_mu3_followupbOncepermonth | -2.25 | -2.21 | 2.39 | -6.25 | 1.67 | 1 | 6,269.33 | 3,648.79 |
| b_mu3_cost$2400 | -3.02 | -2.98 | 2.4 | -7.1 | 0.88 | 1 | 5,774.96 | 3,230.75 |
| b_mu3_cost$6000 | -2.52 | -2.54 | 2.42 | -6.53 | 1.4 | 1 | 7,252.31 | 3,095.86 |
| b_mu3_oobOptedout | -3.79 | -3.74 | 2.17 | -7.46 | -0.28 | 1 | 4,934.35 | 3,247.32 |
| sd_pid__mu2_Intercept | 0.11 | 0.1 | 0.07 | 0.01 | 0.24 | 1 | 1,087.36 | 1,573.83 |
| sd_pid__mu1_Intercept | 0.08 | 0.07 | 0.06 | 0.01 | 0.2 | 1 | 1,703.15 | 2,013.14 |
| sd_pid__mu3_Intercept | 2.45 | 2.4 | 0.5 | 1.73 | 3.34 | 1 | 5,674.10 | 2,836.06 |
| cor_pid__mu2_Intercept__mu1_Intercept | -0.1 | -0.13 | 0.5 | -0.85 | 0.76 | 1 | 4,113.73 | 2,944.20 |
| cor_pid__mu2_Intercept__mu3_Intercept | -0.35 | -0.45 | 0.45 | -0.92 | 0.53 | 1 | 734.28 | 939.3 |
| cor_pid__mu1_Intercept__mu3_Intercept | -0.15 | -0.19 | 0.48 | -0.85 | 0.71 | 1 | 1,208.19 | 1,999.93 |
| r_pid__mu2[R_ADX52844,Intercept] | 0.02 | 0.01 | 0.13 | -0.18 | 0.24 | 1 | 7,500.50 | 3,222.87 |
| r_pid__mu2[R_AEE89210,Intercept] | -0.03 | -0.01 | 0.13 | -0.27 | 0.15 | 1 | 6,174.83 | 3,103.78 |
| r_pid__mu2[R_AET12881,Intercept] | 0 | 0 | 0.12 | -0.19 | 0.21 | 1 | 7,466.95 | 2,772.61 |
| r_pid__mu2[R_AMW22913,Intercept] | -0.03 | -0.01 | 0.12 | -0.26 | 0.15 | 1 | 5,952.02 | 2,867.85 |
| r_pid__mu2[R_APT22198,Intercept] | 0.02 | 0 | 0.13 | -0.18 | 0.25 | 1 | 7,824.99 | 3,286.27 |
| r_pid__mu2[R_ARB38687,Intercept] | 0 | 0 | 0.12 | -0.2 | 0.2 | 1 | 8,076.40 | 3,108.77 |
| r_pid__mu2[R_AXH77869,Intercept] | 0.02 | 0 | 0.12 | -0.17 | 0.24 | 1 | 7,247.80 | 3,017.05 |
| r_pid__mu2[R_BAX11825,Intercept] | 0.02 | 0.01 | 0.12 | -0.17 | 0.23 | 1 | 7,212.15 | 2,779.78 |
| r_pid__mu2[R_BDC43351,Intercept] | 0.04 | 0.01 | 0.13 | -0.14 | 0.28 | 1 | 6,278.41 | 2,688.79 |
| r_pid__mu2[R_BDP76879,Intercept] | -0.01 | 0 | 0.12 | -0.22 | 0.17 | 1 | 7,071.16 | 2,840.29 |
| r_pid__mu2[R_BFK48356,Intercept] | -0.03 | -0.01 | 0.13 | -0.27 | 0.16 | 1 | 7,153.20 | 2,690.02 |
| r_pid__mu2[R_BFX21373,Intercept] | 0 | 0 | 0.12 | -0.2 | 0.2 | 1 | 8,516.01 | 2,949.90 |
| r_pid__mu2[R_BNW41129,Intercept] | 0.02 | 0 | 0.12 | -0.17 | 0.23 | 1 | 7,138.66 | 3,015.74 |
| r_pid__mu2[R_BSH79618,Intercept] | 0 | 0 | 0.12 | -0.19 | 0.21 | 1 | 8,099.88 | 3,144.59 |
| r_pid__mu2[R_BSX76340,Intercept] | 0 | 0 | 0.12 | -0.2 | 0.2 | 1 | 7,712.04 | 3,490.11 |
| r_pid__mu2[R_BTE61611,Intercept] | 0 | 0 | 0.12 | -0.19 | 0.21 | 1 | 7,595.68 | 3,416.49 |
| r_pid__mu2[R_BTJ75900,Intercept] | -0.02 | 0 | 0.13 | -0.24 | 0.17 | 1 | 6,786.33 | 2,797.82 |
| r_pid__mu2[R_CBA74800,Intercept] | 0.02 | 0.01 | 0.12 | -0.16 | 0.23 | 1 | 7,164.82 | 3,359.19 |
| r_pid__mu2[R_CEF14080,Intercept] | 0 | 0 | 0.13 | -0.2 | 0.21 | 1 | 7,315.03 | 3,004.57 |
| r_pid__mu2[R_CGG16808,Intercept] | -0.03 | -0.01 | 0.13 | -0.27 | 0.15 | 1 | 6,080.17 | 3,116.77 |
| r_pid__mu2[R_CGH41789,Intercept] | 0.03 | 0.01 | 0.13 | -0.15 | 0.29 | 1 | 6,257.04 | 2,960.69 |
| r_pid__mu2[R_CND16181,Intercept] | -0.05 | -0.02 | 0.13 | -0.29 | 0.13 | 1 | 4,796.73 | 3,128.98 |
| r_pid__mu2[R_CNM44275,Intercept] | 0.01 | 0 | 0.13 | -0.18 | 0.24 | 1 | 7,083.77 | 2,699.58 |
| r_pid__mu2[R_CPS56694,Intercept] | 0.05 | 0.02 | 0.14 | -0.12 | 0.32 | 1 | 4,398.13 | 3,125.26 |
| r_pid__mu2[R_CRN93379,Intercept] | 0.04 | 0.01 | 0.13 | -0.14 | 0.27 | 1 | 6,128.18 | 3,158.14 |
| r_pid__mu2[R_DAE75724,Intercept] | -0.03 | -0.01 | 0.13 | -0.27 | 0.15 | 1 | 7,077.51 | 3,005.57 |
| r_pid__mu2[R_DCT50457,Intercept] | 0.02 | 0.01 | 0.12 | -0.17 | 0.24 | 1 | 8,274.04 | 3,384.60 |
| r_pid__mu2[R_DEF34111,Intercept] | 0.05 | 0.02 | 0.13 | -0.11 | 0.31 | 1 | 4,846.11 | 3,162.01 |
| r_pid__mu2[R_DKR81851,Intercept] | 0.02 | 0.01 | 0.12 | -0.17 | 0.23 | 1 | 7,751.37 | 3,084.33 |
| r_pid__mu2[R_DMT33649,Intercept] | -0.03 | -0.01 | 0.12 | -0.25 | 0.15 | 1 | 6,015.70 | 3,192.97 |
| r_pid__mu2[R_ECF12320,Intercept] | 0.02 | 0 | 0.12 | -0.17 | 0.23 | 1 | 6,212.34 | 3,289.43 |
| r_pid__mu2[R_ECH91080,Intercept] | 0.02 | 0 | 0.12 | -0.17 | 0.23 | 1 | 7,096.68 | 3,280.95 |
| r_pid__mu2[R_EEW53493,Intercept] | -0.02 | 0 | 0.12 | -0.23 | 0.17 | 1 | 7,882.94 | 3,148.86 |
| r_pid__mu2[R_EGA85998,Intercept] | 0.02 | 0.01 | 0.12 | -0.16 | 0.25 | 1 | 8,401.90 | 2,414.06 |
| r_pid__mu2[R_EJF69850,Intercept] | 0.02 | 0 | 0.13 | -0.18 | 0.23 | 1 | 6,779.07 | 2,634.80 |
| r_pid__mu2[R_EJY87615,Intercept] | 0.02 | 0.01 | 0.12 | -0.17 | 0.23 | 1 | 7,356.18 | 2,649.57 |
| r_pid__mu2[R_ENA26070,Intercept] | 0.03 | 0.01 | 0.13 | -0.15 | 0.28 | 1 | 6,279.18 | 3,101.58 |
| r_pid__mu2[R_ESJ12452,Intercept] | 0.02 | 0 | 0.12 | -0.16 | 0.23 | 1 | 8,193.74 | 3,230.62 |
| r_pid__mu2[R_ETG23474,Intercept] | 0 | 0 | 0.12 | -0.2 | 0.21 | 1 | 8,747.76 | 3,064.49 |
| r_pid__mu2[R_ETP91641,Intercept] | 0 | 0 | 0.12 | -0.19 | 0.2 | 1 | 8,369.20 | 3,334.61 |
| r_pid__mu2[R_EYC37015,Intercept] | 0.02 | 0 | 0.13 | -0.17 | 0.25 | 1 | 7,378.88 | 2,680.04 |
| r_pid__mu2[R_FAK74382,Intercept] | 0.02 | 0 | 0.12 | -0.17 | 0.24 | 1 | 7,113.70 | 3,175.35 |
| r_pid__mu2[R_FNY84524,Intercept] | -0.19 | -0.12 | 0.23 | -0.64 | 0.09 | 1 | 923.03 | 2,438.30 |
| r_pid__mu2[R_FTY43593,Intercept] | -0.05 | -0.02 | 0.13 | -0.3 | 0.13 | 1 | 5,353.23 | 3,337.73 |
| r_pid__mu2[R_GKC46288,Intercept] | 0.03 | 0.01 | 0.13 | -0.14 | 0.26 | 1 | 6,180.18 | 2,718.13 |
| r_pid__mu2[R_HBM13772,Intercept] | 0.02 | 0.01 | 0.12 | -0.16 | 0.22 | 1 | 7,336.86 | 3,667.56 |
| r_pid__mu2[R_HGJ66011,Intercept] | -0.01 | 0 | 0.12 | -0.22 | 0.18 | 1 | 8,619.19 | 3,323.88 |
| r_pid__mu2[R_HPN37609,Intercept] | 0.03 | 0.01 | 0.13 | -0.15 | 0.27 | 1 | 6,288.17 | 3,048.75 |
| r_pid__mu2[R_HTB72919,Intercept] | -0.01 | 0 | 0.12 | -0.23 | 0.18 | 1 | 7,192.70 | 3,056.55 |
| r_pid__mu2[R_HWW23584,Intercept] | 0.02 | 0 | 0.12 | -0.17 | 0.22 | 1 | 7,495.60 | 2,829.66 |
| r_pid__mu2[R_JHE35541,Intercept] | 0.04 | 0.01 | 0.13 | -0.14 | 0.28 | 1 | 4,753.60 | 2,989.63 |
| r_pid__mu2[R_JKA14894,Intercept] | 0 | 0 | 0.12 | -0.2 | 0.21 | 1 | 8,850.59 | 3,294.72 |
| r_pid__mu2[R_JNG26169,Intercept] | -0.03 | -0.01 | 0.12 | -0.27 | 0.15 | 1 | 6,430.38 | 2,861.52 |
| r_pid__mu2[R_JPS35508,Intercept] | -0.01 | 0 | 0.12 | -0.22 | 0.18 | 1 | 7,106.08 | 3,395.60 |
| r_pid__mu2[R_JRM72468,Intercept] | -0.02 | -0.01 | 0.12 | -0.23 | 0.18 | 1 | 7,214.22 | 2,846.23 |
| r_pid__mu2[R_JTS74668,Intercept] | 0 | 0 | 0.12 | -0.2 | 0.22 | 1 | 8,517.05 | 3,395.14 |
| r_pid__mu2[R_JWB53075,Intercept] | -0.01 | 0 | 0.13 | -0.25 | 0.19 | 1 | 7,654.41 | 2,615.49 |
| r_pid__mu2[R_JWW24288,Intercept] | 0.04 | 0.01 | 0.13 | -0.14 | 0.27 | 1 | 5,286.75 | 2,959.41 |
| r_pid__mu2[R_KBX55077,Intercept] | -0.01 | 0 | 0.12 | -0.22 | 0.17 | 1 | 7,019.15 | 3,248.11 |
| r_pid__mu2[R_KFD52470,Intercept] | -0.02 | 0 | 0.13 | -0.25 | 0.18 | 1 | 7,526.37 | 3,099.53 |
| r_pid__mu2[R_KJR20878,Intercept] | -0.02 | -0.01 | 0.12 | -0.23 | 0.18 | 1 | 6,751.67 | 3,084.90 |
| r_pid__mu2[R_KKY83600,Intercept] | 0.02 | 0.01 | 0.12 | -0.18 | 0.23 | 1 | 6,565.83 | 3,022.03 |
| r_pid__mu2[R_KNR62095,Intercept] | 0.02 | 0.01 | 0.12 | -0.16 | 0.23 | 1 | 6,870.97 | 3,253.67 |
| r_pid__mu2[R_KPB60082,Intercept] | 0.03 | 0.01 | 0.12 | -0.15 | 0.27 | 1 | 6,072.98 | 3,324.95 |
| r_pid__mu2[R_KRW92587,Intercept] | 0.02 | 0 | 0.12 | -0.16 | 0.24 | 1 | 7,257.80 | 3,160.10 |
| r_pid__mu2[R_KRY63228,Intercept] | 0 | 0 | 0.12 | -0.19 | 0.2 | 1 | 7,286.54 | 3,166.92 |
| r_pid__mu2[R_KSY61941,Intercept] | -0.05 | -0.02 | 0.13 | -0.29 | 0.13 | 1 | 4,260.44 | 3,139.01 |
| r_pid__mu2[R_KTT74789,Intercept] | 0 | 0 | 0.12 | -0.21 | 0.2 | 1 | 7,876.11 | 3,225.76 |
| r_pid__mu2[R_KWJ44418,Intercept] | 0.02 | 0.01 | 0.13 | -0.17 | 0.25 | 1 | 8,097.27 | 3,223.94 |
| r_pid__mu2[R_KWY94138,Intercept] | 0 | 0 | 0.12 | -0.2 | 0.2 | 1 | 8,119.16 | 3,619.91 |
| r_pid__mu2[R_MBP81026,Intercept] | 0 | 0 | 0.12 | -0.2 | 0.2 | 1 | 8,452.76 | 3,258.23 |
| r_pid__mu2[R_MDA27874,Intercept] | 0.02 | 0 | 0.12 | -0.18 | 0.24 | 1 | 6,595.40 | 3,011.51 |
| r_pid__mu2[R_MEP52349,Intercept] | 0.02 | 0.01 | 0.13 | -0.17 | 0.25 | 1 | 8,697.56 | 3,291.39 |
| r_pid__mu2[R_MHN28908,Intercept] | -0.11 | -0.07 | 0.17 | -0.44 | 0.09 | 1 | 1,138.80 | 2,702.00 |
| r_pid__mu2[R_MHT39842,Intercept] | 0.04 | 0.01 | 0.13 | -0.14 | 0.29 | 1 | 5,861.58 | 3,256.42 |
| r_pid__mu2[R_MRF29260,Intercept] | 0 | 0 | 0.12 | -0.2 | 0.2 | 1 | 8,813.90 | 2,886.63 |
| r_pid__mu2[R_NBM30041,Intercept] | 0.02 | 0.01 | 0.12 | -0.17 | 0.22 | 1 | 7,374.25 | 2,960.07 |
| r_pid__mu2[R_NBP11132,Intercept] | 0 | 0 | 0.12 | -0.2 | 0.21 | 1 | 8,547.04 | 2,676.70 |
| r_pid__mu2[R_NEG35827,Intercept] | -0.01 | 0 | 0.12 | -0.23 | 0.19 | 1 | 7,105.26 | 3,272.22 |
| r_pid__mu2[R_NEX14696,Intercept] | 0 | 0 | 0.12 | -0.21 | 0.2 | 1 | 8,028.44 | 3,152.32 |
| r_pid__mu2[R_NHR51920,Intercept] | 0 | 0 | 0.12 | -0.2 | 0.21 | 1 | 9,571.61 | 3,017.16 |
| r_pid__mu2[R_NPP48092,Intercept] | 0 | 0 | 0.12 | -0.21 | 0.19 | 1 | 9,014.11 | 3,387.71 |
| r_pid__mu2[R_NRE89991,Intercept] | -0.01 | 0 | 0.12 | -0.23 | 0.18 | 1 | 7,961.47 | 3,611.95 |
| r_pid__mu2[R_NRY64108,Intercept] | -0.01 | 0 | 0.12 | -0.23 | 0.18 | 1 | 7,140.94 | 3,263.53 |
| r_pid__mu2[R_NXE26961,Intercept] | -0.02 | -0.01 | 0.12 | -0.23 | 0.17 | 1 | 7,609.46 | 3,332.17 |
| r_pid__mu2[R_PAE91905,Intercept] | 0.01 | 0 | 0.12 | -0.19 | 0.22 | 1 | 9,385.81 | 3,198.39 |
| r_pid__mu2[R_PCY27038,Intercept] | 0.02 | 0 | 0.12 | -0.15 | 0.24 | 1 | 8,348.17 | 3,373.66 |
| r_pid__mu2[R_PEH31350,Intercept] | -0.01 | 0 | 0.12 | -0.24 | 0.17 | 1 | 7,787.08 | 3,366.11 |
| r_pid__mu2[R_PJH58124,Intercept] | 0.02 | 0.01 | 0.12 | -0.16 | 0.23 | 1 | 7,521.94 | 3,093.40 |
| r_pid__mu2[R_PNK26928,Intercept] | 0.02 | 0 | 0.12 | -0.16 | 0.24 | 1 | 7,664.92 | 3,539.69 |
| r_pid__mu2[R_PPP64130,Intercept] | 0 | 0 | 0.12 | -0.2 | 0.21 | 1 | 8,260.23 | 2,781.90 |
| r_pid__mu2[R_PRE99583,Intercept] | -0.03 | -0.01 | 0.12 | -0.26 | 0.15 | 1 | 5,832.34 | 3,079.17 |
| r_pid__mu2[R_PXD76098,Intercept] | -0.03 | -0.01 | 0.12 | -0.25 | 0.15 | 1 | 6,189.34 | 3,147.37 |
| r_pid__mu2[R_PYM49852,Intercept] | -0.01 | 0 | 0.12 | -0.22 | 0.19 | 1 | 8,326.10 | 3,196.46 |
| r_pid__mu2[R_RBX48367,Intercept] | 0.02 | 0 | 0.13 | -0.17 | 0.25 | 1 | 7,836.40 | 3,151.27 |
| r_pid__mu2[R_RDF56375,Intercept] | 0 | 0 | 0.12 | -0.21 | 0.2 | 1 | 8,044.66 | 2,970.48 |
| r_pid__mu2[R_RFJ16126,Intercept] | 0.02 | 0.01 | 0.12 | -0.17 | 0.24 | 1 | 7,060.56 | 3,217.97 |
| r_pid__mu2[R_RJJ20361,Intercept] | 0.03 | 0.01 | 0.12 | -0.15 | 0.26 | 1 | 6,648.42 | 3,121.81 |
| r_pid__mu2[R_SAH46673,Intercept] | 0.02 | 0.01 | 0.12 | -0.17 | 0.24 | 1 | 7,168.58 | 3,290.17 |
| r_pid__mu2[R_SMJ83721,Intercept] | 0.05 | 0.02 | 0.13 | -0.12 | 0.3 | 1 | 4,232.91 | 3,288.90 |
| r_pid__mu2[R_SNF56749,Intercept] | 0.02 | 0 | 0.12 | -0.17 | 0.23 | 1 | 7,584.59 | 2,660.45 |
| r_pid__mu2[R_SRF84799,Intercept] | -0.05 | -0.02 | 0.13 | -0.3 | 0.12 | 1 | 4,723.19 | 3,025.73 |
| r_pid__mu2[R_STY48191,Intercept] | -0.02 | 0 | 0.12 | -0.23 | 0.17 | 1 | 6,744.70 | 2,644.31 |
| r_pid__mu2[R_SXW67243,Intercept] | 0.02 | 0.01 | 0.12 | -0.17 | 0.23 | 1 | 7,277.25 | 3,046.20 |
| r_pid__mu2[R_TCB44726,Intercept] | 0.03 | 0.01 | 0.13 | -0.14 | 0.27 | 1 | 5,247.88 | 3,075.04 |
| r_pid__mu2[R_TCE65670,Intercept] | -0.02 | -0.01 | 0.12 | -0.24 | 0.17 | 1 | 6,968.91 | 3,233.13 |
| r_pid__mu2[R_TCS30250,Intercept] | 0 | 0 | 0.12 | -0.19 | 0.2 | 1 | 7,153.15 | 3,182.52 |
| r_pid__mu2[R_TPC83039,Intercept] | -0.03 | -0.01 | 0.13 | -0.28 | 0.15 | 1 | 6,135.30 | 2,849.64 |
| r_pid__mu2[R_TRP98835,Intercept] | 0 | 0 | 0.12 | -0.19 | 0.2 | 1 | 8,710.58 | 3,225.52 |
| r_pid__mu2[R_TWX60236,Intercept] | 0.03 | 0.01 | 0.13 | -0.14 | 0.27 | 1 | 6,994.39 | 2,970.69 |
| r_pid__mu2[R_TXM75460,Intercept] | 0 | 0 | 0.12 | -0.19 | 0.2 | 1 | 7,134.83 | 2,805.28 |
| r_pid__mu2[R_TYA73238,Intercept] | 0 | 0 | 0.12 | -0.19 | 0.21 | 1 | 8,198.76 | 3,129.77 |
| r_pid__mu2[R_WJT89749,Intercept] | 0 | 0 | 0.12 | -0.2 | 0.19 | 1 | 8,724.57 | 3,244.17 |
| r_pid__mu2[R_WKW46574,Intercept] | 0.03 | 0.01 | 0.12 | -0.13 | 0.26 | 1 | 5,755.30 | 3,279.21 |
| r_pid__mu2[R_WNM42658,Intercept] | 0.02 | 0.01 | 0.12 | -0.17 | 0.23 | 1 | 6,320.81 | 3,186.83 |
| r_pid__mu2[R_WTC54945,Intercept] | -0.15 | -0.1 | 0.2 | -0.53 | 0.11 | 1 | 1,005.79 | 2,650.51 |
| r_pid__mu2[R_WXA34243,Intercept] | 0.03 | 0.01 | 0.12 | -0.14 | 0.26 | 1 | 5,387.74 | 3,010.16 |
| r_pid__mu2[R_XDX16489,Intercept] | 0 | 0 | 0.12 | -0.2 | 0.21 | 1 | 8,697.90 | 3,055.61 |
| r_pid__mu2[R_XJD47234,Intercept] | -0.01 | 0 | 0.13 | -0.24 | 0.18 | 1 | 8,159.44 | 2,262.83 |
| r_pid__mu2[R_XKE91696,Intercept] | -0.02 | 0 | 0.12 | -0.24 | 0.17 | 1 | 7,378.01 | 2,815.08 |
| r_pid__mu2[R_XNA94006,Intercept] | 0.03 | 0.01 | 0.13 | -0.14 | 0.27 | 1 | 6,457.49 | 2,846.72 |
| r_pid__mu2[R_XTF49093,Intercept] | 0 | 0 | 0.12 | -0.19 | 0.19 | 1 | 8,112.34 | 3,475.49 |
| r_pid__mu2[R_XYB26906,Intercept] | 0 | 0 | 0.12 | -0.2 | 0.2 | 1 | 9,292.44 | 2,628.99 |
| r_pid__mu2[R_XYE30129,Intercept] | 0.02 | 0.01 | 0.13 | -0.18 | 0.25 | 1 | 7,106.51 | 3,040.17 |
| r_pid__mu2[R_YBP87571,Intercept] | 0 | 0 | 0.12 | -0.2 | 0.2 | 1 | 9,149.74 | 2,760.55 |
| r_pid__mu2[R_YGA78661,Intercept] | 0.02 | 0 | 0.13 | -0.18 | 0.24 | 1 | 6,803.21 | 3,021.87 |
| r_pid__mu2[R_YSW98879,Intercept] | 0 | 0 | 0.12 | -0.2 | 0.2 | 1 | 7,516.31 | 2,635.64 |
| r_pid__mu2[R_YYH17600,Intercept] | 0.04 | 0.01 | 0.14 | -0.15 | 0.3 | 1 | 5,333.82 | 2,845.59 |
| r_pid__mu1[R_ADX52844,Intercept] | -0.01 | 0 | 0.1 | -0.17 | 0.14 | 1 | 4,945.87 | 3,454.70 |
| r_pid__mu1[R_AEE89210,Intercept] | 0.02 | 0.01 | 0.11 | -0.12 | 0.22 | 1 | 4,380.52 | 3,007.97 |
| r_pid__mu1[R_AET12881,Intercept] | 0.01 | 0 | 0.09 | -0.14 | 0.16 | 1 | 4,827.12 | 3,022.47 |
| r_pid__mu1[R_AMW22913,Intercept] | 0.03 | 0.01 | 0.1 | -0.11 | 0.21 | 1 | 4,771.06 | 2,878.84 |
| r_pid__mu1[R_APT22198,Intercept] | -0.01 | 0 | 0.1 | -0.17 | 0.14 | 1 | 4,728.16 | 3,172.64 |
| r_pid__mu1[R_ARB38687,Intercept] | 0 | 0 | 0.1 | -0.15 | 0.18 | 1 | 5,512.35 | 3,306.31 |
| r_pid__mu1[R_AXH77869,Intercept] | -0.01 | 0 | 0.1 | -0.17 | 0.15 | 1 | 4,841.25 | 2,681.91 |
| r_pid__mu1[R_BAX11825,Intercept] | -0.01 | 0 | 0.1 | -0.17 | 0.15 | 1 | 4,314.20 | 3,413.76 |
| r_pid__mu1[R_BDC43351,Intercept] | -0.02 | 0 | 0.1 | -0.2 | 0.13 | 1 | 5,030.89 | 3,030.23 |
| r_pid__mu1[R_BDP76879,Intercept] | 0.02 | 0 | 0.1 | -0.13 | 0.19 | 1 | 4,933.84 | 3,205.06 |
| r_pid__mu1[R_BFK48356,Intercept] | 0.03 | 0.01 | 0.1 | -0.12 | 0.21 | 1 | 4,283.36 | 3,459.79 |
| r_pid__mu1[R_BFX21373,Intercept] | 0.01 | 0 | 0.1 | -0.15 | 0.17 | 1 | 5,298.80 | 3,478.92 |
| r_pid__mu1[R_BNW41129,Intercept] | -0.01 | 0 | 0.09 | -0.17 | 0.13 | 1 | 4,638.62 | 3,457.62 |
| r_pid__mu1[R_BSH79618,Intercept] | 0 | 0 | 0.1 | -0.15 | 0.17 | 1 | 5,686.74 | 3,858.58 |
| r_pid__mu1[R_BSX76340,Intercept] | 0 | 0 | 0.1 | -0.15 | 0.17 | 1 | 5,237.31 | 2,975.71 |
| r_pid__mu1[R_BTE61611,Intercept] | 0 | 0 | 0.1 | -0.15 | 0.16 | 1 | 6,081.24 | 3,029.33 |
| r_pid__mu1[R_BTJ75900,Intercept] | 0.02 | 0.01 | 0.1 | -0.12 | 0.18 | 1 | 3,737.22 | 3,350.61 |
| r_pid__mu1[R_CBA74800,Intercept] | 0 | 0 | 0.1 | -0.16 | 0.15 | 1 | 4,855.00 | 2,794.44 |
| r_pid__mu1[R_CEF14080,Intercept] | 0 | 0 | 0.1 | -0.15 | 0.17 | 1 | 4,885.16 | 3,465.38 |
| r_pid__mu1[R_CGG16808,Intercept] | 0.03 | 0.01 | 0.1 | -0.11 | 0.21 | 1 | 4,296.49 | 3,277.34 |
| r_pid__mu1[R_CGH41789,Intercept] | -0.02 | -0.01 | 0.1 | -0.19 | 0.12 | 1 | 4,670.67 | 3,171.46 |
| r_pid__mu1[R_CND16181,Intercept] | 0.04 | 0.01 | 0.11 | -0.09 | 0.24 | 1 | 3,631.69 | 3,169.88 |
| r_pid__mu1[R_CNM44275,Intercept] | -0.01 | 0 | 0.1 | -0.17 | 0.15 | 1 | 5,156.53 | 3,154.76 |
| r_pid__mu1[R_CPS56694,Intercept] | -0.03 | -0.01 | 0.1 | -0.21 | 0.11 | 1 | 3,967.71 | 3,123.67 |
| r_pid__mu1[R_CRN93379,Intercept] | -0.02 | 0 | 0.1 | -0.2 | 0.13 | 1 | 5,304.11 | 3,575.06 |
| r_pid__mu1[R_DAE75724,Intercept] | 0.03 | 0.01 | 0.1 | -0.11 | 0.21 | 1 | 4,163.21 | 2,867.51 |
| r_pid__mu1[R_DCT50457,Intercept] | -0.01 | 0 | 0.1 | -0.18 | 0.14 | 1 | 4,549.11 | 3,113.14 |
| r_pid__mu1[R_DEF34111,Intercept] | -0.03 | -0.01 | 0.1 | -0.22 | 0.11 | 1 | 4,450.79 | 3,547.23 |
| r_pid__mu1[R_DKR81851,Intercept] | -0.01 | 0 | 0.09 | -0.17 | 0.14 | 1 | 4,614.61 | 2,456.48 |
| r_pid__mu1[R_DMT33649,Intercept] | 0.02 | 0.01 | 0.1 | -0.12 | 0.21 | 1 | 4,396.45 | 3,033.97 |
| r_pid__mu1[R_ECF12320,Intercept] | -0.01 | 0 | 0.1 | -0.17 | 0.15 | 1 | 4,693.03 | 3,471.55 |
| r_pid__mu1[R_ECH91080,Intercept] | -0.01 | 0 | 0.1 | -0.17 | 0.14 | 1 | 6,739.75 | 3,306.52 |
| r_pid__mu1[R_EEW53493,Intercept] | 0.02 | 0 | 0.1 | -0.12 | 0.19 | 1 | 5,091.35 | 2,883.45 |
| r_pid__mu1[R_EGA85998,Intercept] | -0.01 | 0 | 0.1 | -0.17 | 0.15 | 1 | 5,474.54 | 3,169.89 |
| r_pid__mu1[R_EJF69850,Intercept] | -0.01 | 0 | 0.1 | -0.17 | 0.15 | 1 | 4,883.99 | 3,228.05 |
| r_pid__mu1[R_EJY87615,Intercept] | -0.01 | 0 | 0.1 | -0.17 | 0.14 | 1 | 4,851.63 | 3,343.67 |
| r_pid__mu1[R_ENA26070,Intercept] | -0.02 | 0 | 0.1 | -0.19 | 0.12 | 1 | 5,170.39 | 3,478.40 |
| r_pid__mu1[R_ESJ12452,Intercept] | -0.01 | 0 | 0.1 | -0.17 | 0.15 | 1 | 4,767.29 | 3,438.55 |
| r_pid__mu1[R_ETG23474,Intercept] | 0 | 0 | 0.1 | -0.15 | 0.16 | 1 | 5,481.95 | 3,605.35 |
| r_pid__mu1[R_ETP91641,Intercept] | 0.01 | 0 | 0.1 | -0.15 | 0.17 | 1 | 5,006.01 | 3,388.22 |
| r_pid__mu1[R_EYC37015,Intercept] | -0.01 | 0 | 0.1 | -0.17 | 0.14 | 1 | 5,038.92 | 2,990.05 |
| r_pid__mu1[R_FAK74382,Intercept] | -0.01 | 0 | 0.1 | -0.17 | 0.14 | 1 | 5,091.67 | 3,204.16 |
| r_pid__mu1[R_FNY84524,Intercept] | -0.06 | -0.02 | 0.17 | -0.38 | 0.18 | 1 | 1,718.80 | 2,822.78 |
| r_pid__mu1[R_FTY43593,Intercept] | 0.04 | 0.01 | 0.11 | -0.1 | 0.25 | 1 | 3,915.79 | 2,780.00 |
| r_pid__mu1[R_GKC46288,Intercept] | -0.02 | 0 | 0.1 | -0.2 | 0.12 | 1 | 4,951.57 | 3,031.83 |
| r_pid__mu1[R_HBM13772,Intercept] | -0.01 | 0 | 0.1 | -0.18 | 0.14 | 1 | 4,848.51 | 3,349.32 |
| r_pid__mu1[R_HGJ66011,Intercept] | 0.01 | 0 | 0.1 | -0.13 | 0.19 | 1 | 5,097.63 | 3,201.72 |
| r_pid__mu1[R_HPN37609,Intercept] | -0.02 | -0.01 | 0.1 | -0.19 | 0.13 | 1 | 4,897.61 | 3,390.68 |
| r_pid__mu1[R_HTB72919,Intercept] | 0.02 | 0 | 0.1 | -0.13 | 0.19 | 1 | 4,881.98 | 3,606.16 |
| r_pid__mu1[R_HWW23584,Intercept] | -0.01 | 0 | 0.1 | -0.17 | 0.14 | 1 | 5,928.49 | 3,445.60 |
| r_pid__mu1[R_JHE35541,Intercept] | -0.02 | 0 | 0.1 | -0.21 | 0.13 | 1 | 4,381.30 | 3,303.61 |
| r_pid__mu1[R_JKA14894,Intercept] | 0 | 0 | 0.1 | -0.14 | 0.16 | 1 | 4,953.09 | 3,392.87 |
| r_pid__mu1[R_JNG26169,Intercept] | 0.03 | 0.01 | 0.1 | -0.11 | 0.22 | 1 | 3,975.29 | 3,326.49 |
| r_pid__mu1[R_JPS35508,Intercept] | 0.02 | 0 | 0.1 | -0.12 | 0.18 | 1 | 4,750.85 | 3,194.36 |
| r_pid__mu1[R_JRM72468,Intercept] | 0.02 | 0 | 0.1 | -0.14 | 0.18 | 1 | 5,374.63 | 3,237.85 |
| r_pid__mu1[R_JTS74668,Intercept] | 0.01 | 0 | 0.1 | -0.15 | 0.17 | 1 | 5,187.47 | 3,181.96 |
| r_pid__mu1[R_JWB53075,Intercept] | 0.01 | 0 | 0.1 | -0.14 | 0.19 | 1 | 5,101.75 | 3,160.67 |
| r_pid__mu1[R_JWW24288,Intercept] | -0.02 | 0 | 0.1 | -0.19 | 0.12 | 1 | 4,419.28 | 2,634.51 |
| r_pid__mu1[R_KBX55077,Intercept] | 0.01 | 0 | 0.1 | -0.14 | 0.19 | 1 | 5,131.03 | 2,870.56 |
| r_pid__mu1[R_KFD52470,Intercept] | 0.02 | 0 | 0.1 | -0.14 | 0.19 | 1 | 4,573.06 | 3,028.27 |
| r_pid__mu1[R_KJR20878,Intercept] | 0.01 | 0 | 0.1 | -0.13 | 0.19 | 1 | 4,932.59 | 3,675.81 |
| r_pid__mu1[R_KKY83600,Intercept] | 0 | 0 | 0.1 | -0.17 | 0.16 | 1 | 5,855.17 | 3,442.74 |
| r_pid__mu1[R_KNR62095,Intercept] | -0.01 | 0 | 0.1 | -0.16 | 0.15 | 1 | 5,541.26 | 3,333.83 |
| r_pid__mu1[R_KPB60082,Intercept] | -0.02 | 0 | 0.1 | -0.2 | 0.13 | 1 | 4,318.70 | 3,211.61 |
| r_pid__mu1[R_KRW92587,Intercept] | -0.01 | 0 | 0.1 | -0.17 | 0.15 | 1 | 5,027.52 | 3,312.91 |
| r_pid__mu1[R_KRY63228,Intercept] | 0 | 0 | 0.1 | -0.15 | 0.17 | 1 | 5,409.21 | 2,173.65 |
| r_pid__mu1[R_KSY61941,Intercept] | 0.04 | 0.01 | 0.11 | -0.1 | 0.25 | 1 | 4,187.99 | 3,551.38 |
| r_pid__mu1[R_KTT74789,Intercept] | 0 | 0 | 0.1 | -0.15 | 0.16 | 1 | 5,343.32 | 3,439.49 |
| r_pid__mu1[R_KWJ44418,Intercept] | -0.01 | 0 | 0.1 | -0.18 | 0.15 | 1 | 5,267.25 | 3,067.28 |
| r_pid__mu1[R_KWY94138,Intercept] | 0 | 0 | 0.09 | -0.15 | 0.16 | 1 | 4,712.47 | 3,164.79 |
| r_pid__mu1[R_MBP81026,Intercept] | 0.01 | 0 | 0.1 | -0.14 | 0.17 | 1 | 5,135.58 | 3,265.10 |
| r_pid__mu1[R_MDA27874,Intercept] | -0.01 | 0 | 0.1 | -0.17 | 0.16 | 1 | 4,968.05 | 2,790.60 |
| r_pid__mu1[R_MEP52349,Intercept] | -0.01 | 0 | 0.1 | -0.18 | 0.15 | 1 | 5,031.41 | 3,283.37 |
| r_pid__mu1[R_MHN28908,Intercept] | -0.04 | -0.01 | 0.13 | -0.28 | 0.15 | 1 | 1,896.60 | 2,741.49 |
| r_pid__mu1[R_MHT39842,Intercept] | -0.02 | 0 | 0.1 | -0.19 | 0.13 | 1 | 4,686.91 | 3,468.43 |
| r_pid__mu1[R_MRF29260,Intercept] | 0.01 | 0 | 0.1 | -0.14 | 0.18 | 1 | 4,795.24 | 2,841.20 |
| r_pid__mu1[R_NBM30041,Intercept] | -0.01 | 0 | 0.09 | -0.16 | 0.14 | 1 | 4,913.53 | 2,923.40 |
| r_pid__mu1[R_NBP11132,Intercept] | 0.01 | 0 | 0.1 | -0.15 | 0.17 | 1 | 5,223.56 | 3,200.56 |
| r_pid__mu1[R_NEG35827,Intercept] | 0.01 | 0 | 0.1 | -0.13 | 0.18 | 1 | 4,209.81 | 3,304.08 |
| r_pid__mu1[R_NEX14696,Intercept] | 0.01 | 0 | 0.1 | -0.15 | 0.18 | 1 | 5,134.44 | 3,368.37 |
| r_pid__mu1[R_NHR51920,Intercept] | 0 | 0 | 0.09 | -0.15 | 0.16 | 1 | 4,694.41 | 3,412.90 |
| r_pid__mu1[R_NPP48092,Intercept] | 0 | 0 | 0.1 | -0.15 | 0.17 | 1 | 5,540.02 | 3,157.70 |
| r_pid__mu1[R_NRE89991,Intercept] | 0.02 | 0 | 0.1 | -0.13 | 0.19 | 1 | 4,796.79 | 3,377.23 |
| r_pid__mu1[R_NRY64108,Intercept] | 0.01 | 0 | 0.1 | -0.14 | 0.18 | 1 | 4,954.72 | 3,329.45 |
| r_pid__mu1[R_NXE26961,Intercept] | 0.01 | 0 | 0.1 | -0.13 | 0.18 | 1 | 5,490.80 | 3,219.65 |
| r_pid__mu1[R_PAE91905,Intercept] | 0 | 0 | 0.1 | -0.15 | 0.17 | 1 | 5,072.26 | 3,145.93 |
| r_pid__mu1[R_PCY27038,Intercept] | -0.01 | 0 | 0.1 | -0.17 | 0.14 | 1 | 5,238.56 | 3,423.38 |
| r_pid__mu1[R_PEH31350,Intercept] | 0.01 | 0 | 0.1 | -0.13 | 0.19 | 1 | 5,727.83 | 3,225.83 |
| r_pid__mu1[R_PJH58124,Intercept] | -0.01 | 0 | 0.1 | -0.18 | 0.15 | 1 | 4,934.36 | 3,390.98 |
| r_pid__mu1[R_PNK26928,Intercept] | -0.01 | 0 | 0.1 | -0.17 | 0.15 | 1 | 4,919.17 | 3,107.47 |
| r_pid__mu1[R_PPP64130,Intercept] | 0 | 0 | 0.1 | -0.16 | 0.17 | 1 | 4,974.25 | 3,582.65 |
| r_pid__mu1[R_PRE99583,Intercept] | 0.03 | 0.01 | 0.1 | -0.11 | 0.22 | 1 | 3,959.19 | 3,093.66 |
| r_pid__mu1[R_PXD76098,Intercept] | 0.03 | 0.01 | 0.1 | -0.12 | 0.22 | 1 | 4,383.71 | 3,329.17 |
| r_pid__mu1[R_PYM49852,Intercept] | 0.02 | 0 | 0.1 | -0.13 | 0.18 | 1 | 4,879.77 | 3,579.04 |
| r_pid__mu1[R_RBX48367,Intercept] | 0 | 0 | 0.1 | -0.17 | 0.15 | 1 | 5,408.36 | 3,133.26 |
| r_pid__mu1[R_RDF56375,Intercept] | 0 | 0 | 0.09 | -0.14 | 0.16 | 1 | 5,190.33 | 3,647.46 |
| r_pid__mu1[R_RFJ16126,Intercept] | -0.01 | 0 | 0.1 | -0.17 | 0.14 | 1 | 5,020.00 | 2,925.54 |
| r_pid__mu1[R_RJJ20361,Intercept] | -0.02 | 0 | 0.1 | -0.21 | 0.12 | 1 | 4,552.27 | 3,033.65 |
| r_pid__mu1[R_SAH46673,Intercept] | -0.01 | 0 | 0.09 | -0.17 | 0.14 | 1 | 5,211.12 | 2,640.36 |
| r_pid__mu1[R_SMJ83721,Intercept] | -0.03 | -0.01 | 0.1 | -0.22 | 0.12 | 1 | 4,290.10 | 3,020.82 |
| r_pid__mu1[R_SNF56749,Intercept] | -0.01 | 0 | 0.1 | -0.17 | 0.15 | 1 | 5,250.96 | 3,510.11 |
| r_pid__mu1[R_SRF84799,Intercept] | 0.04 | 0.01 | 0.11 | -0.11 | 0.25 | 1 | 4,121.50 | 2,927.81 |
| r_pid__mu1[R_STY48191,Intercept] | 0.02 | 0 | 0.1 | -0.13 | 0.19 | 1 | 4,566.24 | 3,670.05 |
| r_pid__mu1[R_SXW67243,Intercept] | -0.01 | 0 | 0.09 | -0.17 | 0.14 | 1 | 4,514.50 | 3,116.48 |
| r_pid__mu1[R_TCB44726,Intercept] | -0.02 | 0 | 0.1 | -0.2 | 0.12 | 1 | 4,924.09 | 3,509.82 |
| r_pid__mu1[R_TCE65670,Intercept] | 0.02 | 0 | 0.1 | -0.13 | 0.19 | 1 | 5,108.23 | 3,166.63 |
| r_pid__mu1[R_TCS30250,Intercept] | 0 | 0 | 0.09 | -0.15 | 0.15 | 1 | 4,939.87 | 3,540.09 |
| r_pid__mu1[R_TPC83039,Intercept] | 0.02 | 0.01 | 0.11 | -0.12 | 0.22 | 1 | 4,484.63 | 2,746.51 |
| r_pid__mu1[R_TRP98835,Intercept] | 0 | 0 | 0.09 | -0.15 | 0.16 | 1 | 4,581.38 | 3,311.83 |
| r_pid__mu1[R_TWX60236,Intercept] | -0.02 | 0 | 0.1 | -0.21 | 0.12 | 1 | 4,576.05 | 2,917.67 |
| r_pid__mu1[R_TXM75460,Intercept] | 0 | 0 | 0.1 | -0.15 | 0.16 | 1 | 4,935.89 | 3,227.94 |
| r_pid__mu1[R_TYA73238,Intercept] | 0 | 0 | 0.09 | -0.15 | 0.16 | 1 | 5,354.47 | 3,228.45 |
| r_pid__mu1[R_WJT89749,Intercept] | 0 | 0 | 0.1 | -0.15 | 0.16 | 1 | 5,517.99 | 3,507.47 |
| r_pid__mu1[R_WKW46574,Intercept] | -0.02 | 0 | 0.1 | -0.21 | 0.13 | 1 | 4,690.66 | 3,381.53 |
| r_pid__mu1[R_WNM42658,Intercept] | -0.01 | 0 | 0.1 | -0.17 | 0.14 | 1 | 5,220.81 | 3,074.30 |
| r_pid__mu1[R_WTC54945,Intercept] | -0.06 | -0.02 | 0.16 | -0.35 | 0.16 | 1 | 1,571.58 | 2,993.54 |
| r_pid__mu1[R_WXA34243,Intercept] | -0.02 | -0.01 | 0.1 | -0.2 | 0.13 | 1 | 4,687.69 | 3,268.21 |
| r_pid__mu1[R_XDX16489,Intercept] | 0 | 0 | 0.1 | -0.15 | 0.18 | 1 | 5,850.51 | 3,642.62 |
| r_pid__mu1[R_XJD47234,Intercept] | 0.02 | 0 | 0.1 | -0.13 | 0.2 | 1 | 4,280.30 | 3,037.37 |
| r_pid__mu1[R_XKE91696,Intercept] | 0.01 | 0 | 0.1 | -0.13 | 0.19 | 1 | 5,419.62 | 3,294.90 |
| r_pid__mu1[R_XNA94006,Intercept] | -0.02 | 0 | 0.1 | -0.19 | 0.13 | 1 | 4,888.12 | 3,156.14 |
| r_pid__mu1[R_XTF49093,Intercept] | 0.01 | 0 | 0.1 | -0.15 | 0.17 | 1 | 5,323.83 | 3,185.43 |
| r_pid__mu1[R_XYB26906,Intercept] | 0.01 | 0 | 0.09 | -0.14 | 0.17 | 1 | 5,190.22 | 2,834.97 |
| r_pid__mu1[R_XYE30129,Intercept] | -0.01 | 0 | 0.1 | -0.17 | 0.14 | 1 | 4,935.60 | 3,066.64 |
| r_pid__mu1[R_YBP87571,Intercept] | 0 | 0 | 0.1 | -0.15 | 0.16 | 1 | 4,716.82 | 3,394.14 |
| r_pid__mu1[R_YGA78661,Intercept] | -0.01 | 0 | 0.1 | -0.17 | 0.14 | 1 | 4,606.09 | 3,437.10 |
| r_pid__mu1[R_YSW98879,Intercept] | 0 | 0 | 0.1 | -0.15 | 0.16 | 1 | 4,870.13 | 3,101.26 |
| r_pid__mu1[R_YYH17600,Intercept] | -0.02 | 0 | 0.1 | -0.2 | 0.13 | 1 | 4,444.05 | 3,044.59 |
| r_pid__mu3[R_ADX52844,Intercept] | -0.26 | -0.18 | 2.37 | -4.28 | 3.47 | 1 | 7,283.08 | 3,283.35 |
| r_pid__mu3[R_AEE89210,Intercept] | -0.12 | -0.04 | 2.35 | -4.14 | 3.57 | 1 | 5,486.88 | 3,259.28 |
| r_pid__mu3[R_AET12881,Intercept] | -0.23 | -0.18 | 2.24 | -4.07 | 3.29 | 1 | 7,136.17 | 3,216.53 |
| r_pid__mu3[R_AMW22913,Intercept] | -0.1 | -0.05 | 2.29 | -3.96 | 3.55 | 1 | 5,306.07 | 3,081.11 |
| r_pid__mu3[R_APT22198,Intercept] | -0.27 | -0.22 | 2.32 | -4.2 | 3.4 | 1 | 6,626.11 | 3,234.67 |
| r_pid__mu3[R_ARB38687,Intercept] | -0.21 | -0.18 | 2.29 | -3.95 | 3.46 | 1 | 6,443.55 | 3,325.56 |
| r_pid__mu3[R_AXH77869,Intercept] | -0.28 | -0.2 | 2.29 | -4.22 | 3.4 | 1 | 6,932.94 | 3,258.78 |
| r_pid__mu3[R_BAX11825,Intercept] | -0.3 | -0.23 | 2.3 | -4.25 | 3.38 | 1 | 6,837.98 | 3,500.54 |
| r_pid__mu3[R_BDC43351,Intercept] | -0.39 | -0.31 | 2.33 | -4.28 | 3.25 | 1 | 6,200.59 | 3,025.26 |
| r_pid__mu3[R_BDP76879,Intercept] | -0.14 | -0.13 | 2.25 | -3.93 | 3.47 | 1 | 5,748.79 | 3,556.43 |
| r_pid__mu3[R_BFK48356,Intercept] | -0.11 | -0.1 | 2.36 | -4.09 | 3.71 | 1 | 5,967.76 | 3,228.78 |
| r_pid__mu3[R_BFX21373,Intercept] | -0.19 | -0.13 | 2.27 | -3.93 | 3.38 | 1 | 6,435.10 | 3,084.81 |
| r_pid__mu3[R_BNW41129,Intercept] | -0.26 | -0.15 | 2.32 | -4.32 | 3.33 | 1 | 6,576.51 | 3,036.02 |
| r_pid__mu3[R_BSH79618,Intercept] | -0.22 | -0.12 | 2.34 | -4.16 | 3.54 | 1 | 6,604.15 | 3,054.08 |
| r_pid__mu3[R_BSX76340,Intercept] | -0.24 | -0.18 | 2.29 | -4.13 | 3.42 | 1 | 6,444.79 | 2,903.34 |
| r_pid__mu3[R_BTE61611,Intercept] | -0.26 | -0.14 | 2.31 | -4.16 | 3.36 | 1 | 7,456.17 | 3,370.22 |
| r_pid__mu3[R_BTJ75900,Intercept] | -0.12 | -0.07 | 2.28 | -3.97 | 3.48 | 1 | 6,207.72 | 3,201.22 |
| r_pid__mu3[R_CBA74800,Intercept] | -0.3 | -0.24 | 2.23 | -4.14 | 3.18 | 1 | 6,784.20 | 3,202.06 |
| r_pid__mu3[R_CEF14080,Intercept] | -0.24 | -0.17 | 2.32 | -4.06 | 3.38 | 1 | 6,286.70 | 2,934.85 |
| r_pid__mu3[R_CGG16808,Intercept] | -0.04 | 0.05 | 2.3 | -4.01 | 3.62 | 1 | 6,480.17 | 3,350.37 |
| r_pid__mu3[R_CGH41789,Intercept] | -0.29 | -0.23 | 2.35 | -4.32 | 3.34 | 1 | 6,345.52 | 3,544.19 |
| r_pid__mu3[R_CND16181,Intercept] | -0.06 | 0.01 | 2.32 | -4 | 3.56 | 1 | 5,127.79 | 3,461.56 |
| r_pid__mu3[R_CNM44275,Intercept] | -0.23 | -0.14 | 2.31 | -4.21 | 3.47 | 1 | 7,013.73 | 3,040.57 |
| r_pid__mu3[R_CPS56694,Intercept] | -0.43 | -0.36 | 2.38 | -4.47 | 3.46 | 1 | 5,267.42 | 3,251.40 |
| r_pid__mu3[R_CRN93379,Intercept] | -0.41 | -0.34 | 2.36 | -4.41 | 3.22 | 1 | 6,385.39 | 3,376.54 |
| r_pid__mu3[R_DAE75724,Intercept] | -0.06 | 0 | 2.34 | -4.1 | 3.55 | 1 | 5,397.42 | 2,963.23 |
| r_pid__mu3[R_DCT50457,Intercept] | -0.28 | -0.18 | 2.32 | -4.21 | 3.35 | 1 | 7,064.47 | 3,446.76 |
| r_pid__mu3[R_DEF34111,Intercept] | -0.46 | -0.4 | 2.28 | -4.23 | 3.15 | 1 | 5,410.33 | 3,023.49 |
| r_pid__mu3[R_DKR81851,Intercept] | -0.29 | -0.23 | 2.31 | -4.14 | 3.42 | 1 | 6,445.02 | 3,039.75 |
| r_pid__mu3[R_DMT33649,Intercept] | -0.09 | 0.04 | 2.33 | -4.14 | 3.47 | 1 | 5,517.59 | 3,261.84 |
| r_pid__mu3[R_ECF12320,Intercept] | -0.34 | -0.24 | 2.33 | -4.17 | 3.33 | 1 | 6,487.32 | 3,275.64 |
| r_pid__mu3[R_ECH91080,Intercept] | -0.29 | -0.22 | 2.32 | -4.21 | 3.42 | 1 | 7,351.07 | 3,612.16 |
| r_pid__mu3[R_EEW53493,Intercept] | -0.13 | -0.04 | 2.25 | -3.95 | 3.49 | 1 | 6,718.84 | 3,206.14 |
| r_pid__mu3[R_EGA85998,Intercept] | -0.29 | -0.26 | 2.28 | -4.11 | 3.38 | 1 | 6,785.30 | 3,487.61 |
| r_pid__mu3[R_EJF69850,Intercept] | -0.26 | -0.15 | 2.35 | -4.33 | 3.49 | 1 | 5,772.51 | 3,585.23 |
| r_pid__mu3[R_EJY87615,Intercept] | -0.32 | -0.29 | 2.25 | -3.97 | 3.36 | 1 | 7,112.04 | 3,454.72 |
| r_pid__mu3[R_ENA26070,Intercept] | -0.33 | -0.25 | 2.31 | -4.16 | 3.36 | 1 | 5,723.05 | 3,206.47 |
| r_pid__mu3[R_ESJ12452,Intercept] | -0.3 | -0.24 | 2.29 | -4.12 | 3.33 | 1 | 6,317.51 | 3,419.12 |
| r_pid__mu3[R_ETG23474,Intercept] | -0.19 | -0.1 | 2.23 | -3.97 | 3.37 | 1 | 6,136.68 | 3,451.05 |
| r_pid__mu3[R_ETP91641,Intercept] | -0.23 | -0.21 | 2.36 | -4.16 | 3.47 | 1 | 6,677.94 | 2,632.91 |
| r_pid__mu3[R_EYC37015,Intercept] | -0.24 | -0.15 | 2.29 | -4.12 | 3.3 | 1 | 6,301.30 | 2,875.07 |
| r_pid__mu3[R_FAK74382,Intercept] | -0.29 | -0.21 | 2.33 | -4.34 | 3.43 | 1 | 6,472.96 | 3,247.86 |
| r_pid__mu3[R_FNY84524,Intercept] | 7.23 | 7.18 | 1.17 | 5.39 | 9.17 | 1 | 5,444.82 | 3,704.05 |
| r_pid__mu3[R_FTY43593,Intercept] | -0.06 | 0.01 | 2.33 | -4.15 | 3.57 | 1 | 5,732.71 | 3,661.32 |
| r_pid__mu3[R_GKC46288,Intercept] | -0.3 | -0.2 | 2.36 | -4.26 | 3.43 | 1 | 5,288.48 | 3,017.76 |
| r_pid__mu3[R_HBM13772,Intercept] | -0.29 | -0.21 | 2.34 | -4.17 | 3.4 | 1 | 6,456.49 | 3,394.90 |
| r_pid__mu3[R_HGJ66011,Intercept] | -0.14 | -0.05 | 2.28 | -4.07 | 3.43 | 1 | 6,611.80 | 3,667.15 |
| r_pid__mu3[R_HPN37609,Intercept] | -0.33 | -0.25 | 2.32 | -4.17 | 3.34 | 1 | 6,956.12 | 3,664.21 |
| r_pid__mu3[R_HTB72919,Intercept] | -0.15 | -0.05 | 2.31 | -4.09 | 3.55 | 1 | 6,686.23 | 3,230.86 |
| r_pid__mu3[R_HWW23584,Intercept] | -0.28 | -0.17 | 2.33 | -4.2 | 3.45 | 1 | 6,469.68 | 3,446.42 |
| r_pid__mu3[R_JHE35541,Intercept] | -0.39 | -0.29 | 2.37 | -4.35 | 3.33 | 1 | 6,144.13 | 3,213.71 |
| r_pid__mu3[R_JKA14894,Intercept] | -0.23 | -0.2 | 2.27 | -4.02 | 3.35 | 1 | 6,148.07 | 3,477.86 |
| r_pid__mu3[R_JNG26169,Intercept] | -0.05 | 0.02 | 2.26 | -3.97 | 3.47 | 1 | 5,430.29 | 3,429.15 |
| r_pid__mu3[R_JPS35508,Intercept] | -0.17 | -0.1 | 2.26 | -4.01 | 3.34 | 1 | 5,163.08 | 3,207.79 |
| r_pid__mu3[R_JRM72468,Intercept] | -0.15 | -0.08 | 2.29 | -4.06 | 3.51 | 1 | 5,765.33 | 2,829.53 |
| r_pid__mu3[R_JTS74668,Intercept] | -0.18 | -0.1 | 2.26 | -4.04 | 3.46 | 1 | 5,541.31 | 3,300.05 |
| r_pid__mu3[R_JWB53075,Intercept] | -0.17 | -0.1 | 2.36 | -4.16 | 3.57 | 1 | 6,223.56 | 3,059.17 |
| r_pid__mu3[R_JWW24288,Intercept] | -0.35 | -0.24 | 2.32 | -4.24 | 3.29 | 1 | 5,816.84 | 2,750.26 |
| r_pid__mu3[R_KBX55077,Intercept] | -0.14 | -0.05 | 2.28 | -4.02 | 3.48 | 1 | 5,640.56 | 2,791.53 |
| r_pid__mu3[R_KFD52470,Intercept] | -0.18 | -0.16 | 2.28 | -4.04 | 3.48 | 1 | 5,822.43 | 3,571.18 |
| r_pid__mu3[R_KJR20878,Intercept] | -0.15 | -0.07 | 2.29 | -4.05 | 3.45 | 1 | 6,819.04 | 3,355.89 |
| r_pid__mu3[R_KKY83600,Intercept] | -0.26 | -0.2 | 2.34 | -4.24 | 3.5 | 1 | 7,023.95 | 3,637.58 |
| r_pid__mu3[R_KNR62095,Intercept] | -0.26 | -0.17 | 2.29 | -4.27 | 3.42 | 1 | 6,192.69 | 3,319.93 |
| r_pid__mu3[R_KPB60082,Intercept] | -0.33 | -0.31 | 2.32 | -4.2 | 3.35 | 1 | 6,667.15 | 3,125.14 |
| r_pid__mu3[R_KRW92587,Intercept] | -0.24 | -0.15 | 2.3 | -4.15 | 3.49 | 1 | 5,720.22 | 3,618.47 |
| r_pid__mu3[R_KRY63228,Intercept] | -0.19 | -0.15 | 2.25 | -3.99 | 3.38 | 1 | 6,872.21 | 3,477.76 |
| r_pid__mu3[R_KSY61941,Intercept] | -0.09 | -0.02 | 2.32 | -4.08 | 3.6 | 1 | 5,825.97 | 3,369.50 |
| r_pid__mu3[R_KTT74789,Intercept] | -0.23 | -0.19 | 2.25 | -4.03 | 3.3 | 1 | 6,574.52 | 3,255.31 |
| r_pid__mu3[R_KWJ44418,Intercept] | -0.33 | -0.26 | 2.32 | -4.18 | 3.33 | 1 | 5,786.91 | 2,689.65 |
| r_pid__mu3[R_KWY94138,Intercept] | -0.18 | -0.14 | 2.24 | -3.98 | 3.46 | 1 | 6,160.14 | 3,068.69 |
| r_pid__mu3[R_MBP81026,Intercept] | -0.27 | -0.18 | 2.37 | -4.3 | 3.4 | 1 | 7,979.61 | 3,231.35 |
| r_pid__mu3[R_MDA27874,Intercept] | -0.29 | -0.2 | 2.31 | -4.11 | 3.49 | 1 | 6,676.15 | 2,953.63 |
| r_pid__mu3[R_MEP52349,Intercept] | -0.31 | -0.22 | 2.33 | -4.28 | 3.32 | 1 | 5,896.84 | 2,858.94 |
| r_pid__mu3[R_MHN28908,Intercept] | 5.01 | 5.06 | 1.44 | 2.65 | 7.38 | 1 | 4,303.76 | 3,122.41 |
| r_pid__mu3[R_MHT39842,Intercept] | -0.35 | -0.3 | 2.32 | -4.21 | 3.3 | 1 | 6,739.36 | 3,514.35 |
| r_pid__mu3[R_MRF29260,Intercept] | -0.23 | -0.18 | 2.32 | -4.19 | 3.35 | 1 | 6,883.29 | 3,650.76 |
| r_pid__mu3[R_NBM30041,Intercept] | -0.28 | -0.24 | 2.35 | -4.31 | 3.42 | 1 | 6,719.71 | 3,291.79 |
| r_pid__mu3[R_NBP11132,Intercept] | -0.25 | -0.16 | 2.43 | -4.42 | 3.57 | 1 | 7,840.00 | 3,276.96 |
| r_pid__mu3[R_NEG35827,Intercept] | -0.16 | -0.06 | 2.29 | -4.1 | 3.42 | 1 | 5,433.39 | 3,082.36 |
| r_pid__mu3[R_NEX14696,Intercept] | -0.24 | -0.21 | 2.38 | -4.24 | 3.56 | 1 | 7,161.93 | 2,931.39 |
| r_pid__mu3[R_NHR51920,Intercept] | -0.2 | -0.17 | 2.29 | -4.03 | 3.54 | 1 | 6,869.84 | 3,057.06 |
| r_pid__mu3[R_NPP48092,Intercept] | -0.25 | -0.13 | 2.3 | -4.21 | 3.43 | 1 | 6,640.71 | 2,940.72 |
| r_pid__mu3[R_NRE89991,Intercept] | -0.17 | -0.06 | 2.36 | -4.22 | 3.43 | 1 | 6,356.85 | 2,663.52 |
| r_pid__mu3[R_NRY64108,Intercept] | -0.17 | -0.09 | 2.22 | -3.98 | 3.37 | 1 | 6,608.75 | 3,137.57 |
| r_pid__mu3[R_NXE26961,Intercept] | -0.15 | -0.01 | 2.31 | -4.03 | 3.47 | 1 | 6,383.43 | 3,293.05 |
| r_pid__mu3[R_PAE91905,Intercept] | -0.22 | -0.17 | 2.37 | -4.28 | 3.52 | 1 | 6,483.63 | 3,162.68 |
| r_pid__mu3[R_PCY27038,Intercept] | -0.3 | -0.19 | 2.29 | -4.24 | 3.3 | 1 | 6,363.32 | 3,397.83 |
| r_pid__mu3[R_PEH31350,Intercept] | -0.19 | -0.12 | 2.31 | -4.08 | 3.51 | 1 | 7,108.46 | 2,915.67 |
| r_pid__mu3[R_PJH58124,Intercept] | -0.34 | -0.28 | 2.25 | -4.17 | 3.3 | 1 | 6,337.56 | 3,412.76 |
| r_pid__mu3[R_PNK26928,Intercept] | -0.23 | -0.17 | 2.25 | -3.98 | 3.39 | 1 | 6,269.98 | 3,261.38 |
| r_pid__mu3[R_PPP64130,Intercept] | -0.19 | -0.11 | 2.36 | -4.18 | 3.52 | 1 | 7,244.37 | 3,470.66 |
| r_pid__mu3[R_PRE99583,Intercept] | -0.16 | -0.08 | 2.29 | -4.07 | 3.47 | 1 | 5,279.17 | 3,096.61 |
| r_pid__mu3[R_PXD76098,Intercept] | -0.08 | 0.01 | 2.34 | -4.05 | 3.57 | 1 | 5,899.65 | 3,389.99 |
| r_pid__mu3[R_PYM49852,Intercept] | -0.18 | -0.13 | 2.37 | -4.28 | 3.59 | 1 | 6,682.84 | 2,839.31 |
| r_pid__mu3[R_RBX48367,Intercept] | -0.31 | -0.23 | 2.41 | -4.33 | 3.49 | 1 | 6,658.26 | 3,112.18 |
| r_pid__mu3[R_RDF56375,Intercept] | -0.16 | -0.08 | 2.3 | -4 | 3.52 | 1 | 6,165.63 | 2,971.59 |
| r_pid__mu3[R_RFJ16126,Intercept] | -0.26 | -0.24 | 2.25 | -4.06 | 3.34 | 1 | 7,086.90 | 3,426.81 |
| r_pid__mu3[R_RJJ20361,Intercept] | -0.3 | -0.23 | 2.27 | -4.07 | 3.25 | 1 | 6,258.63 | 3,218.56 |
| r_pid__mu3[R_SAH46673,Intercept] | -0.26 | -0.21 | 2.27 | -4.11 | 3.3 | 1 | 6,237.95 | 3,057.84 |
| r_pid__mu3[R_SMJ83721,Intercept] | -0.4 | -0.3 | 2.33 | -4.41 | 3.29 | 1 | 5,177.06 | 3,328.60 |
| r_pid__mu3[R_SNF56749,Intercept] | -0.26 | -0.2 | 2.3 | -4.16 | 3.39 | 1 | 7,006.39 | 3,216.90 |
| r_pid__mu3[R_SRF84799,Intercept] | -0.07 | 0.03 | 2.36 | -4.15 | 3.61 | 1 | 5,011.14 | 3,540.96 |
| r_pid__mu3[R_STY48191,Intercept] | -0.11 | -0.01 | 2.23 | -3.91 | 3.51 | 1 | 5,776.92 | 2,804.89 |
| r_pid__mu3[R_SXW67243,Intercept] | -0.24 | -0.18 | 2.29 | -4.08 | 3.37 | 1 | 5,741.56 | 3,129.22 |
| r_pid__mu3[R_TCB44726,Intercept] | -0.29 | -0.25 | 2.27 | -4.19 | 3.35 | 1 | 5,912.47 | 3,313.12 |
| r_pid__mu3[R_TCE65670,Intercept] | -0.16 | -0.15 | 2.31 | -4.02 | 3.47 | 1 | 6,933.52 | 3,153.96 |
| r_pid__mu3[R_TCS30250,Intercept] | -0.21 | -0.16 | 2.27 | -4.12 | 3.37 | 1 | 6,727.90 | 3,265.15 |
| r_pid__mu3[R_TPC83039,Intercept] | -0.12 | -0.03 | 2.31 | -4.01 | 3.51 | 1 | 6,232.90 | 3,049.55 |
| r_pid__mu3[R_TRP98835,Intercept] | -0.22 | -0.15 | 2.28 | -4.03 | 3.37 | 1 | 7,034.33 | 2,990.24 |
| r_pid__mu3[R_TWX60236,Intercept] | -0.38 | -0.27 | 2.39 | -4.52 | 3.4 | 1 | 6,657.16 | 3,271.57 |
| r_pid__mu3[R_TXM75460,Intercept] | -0.27 | -0.16 | 2.33 | -4.29 | 3.39 | 1 | 5,507.43 | 3,011.99 |
| r_pid__mu3[R_TYA73238,Intercept] | -0.17 | -0.1 | 2.27 | -4.03 | 3.4 | 1 | 5,952.25 | 3,043.43 |
| r_pid__mu3[R_WJT89749,Intercept] | -0.2 | -0.09 | 2.27 | -4.09 | 3.37 | 1 | 6,180.23 | 2,912.53 |
| r_pid__mu3[R_WKW46574,Intercept] | -0.35 | -0.28 | 2.39 | -4.41 | 3.41 | 1 | 5,868.16 | 3,417.00 |
| r_pid__mu3[R_WNM42658,Intercept] | -0.25 | -0.2 | 2.34 | -4.25 | 3.46 | 1 | 6,261.58 | 3,358.09 |
| r_pid__mu3[R_WTC54945,Intercept] | 6.37 | 6.35 | 1.23 | 4.45 | 8.46 | 1 | 5,093.71 | 3,100.71 |
| r_pid__mu3[R_WXA34243,Intercept] | -0.38 | -0.27 | 2.33 | -4.29 | 3.24 | 1 | 5,994.00 | 3,057.11 |
| r_pid__mu3[R_XDX16489,Intercept] | -0.23 | -0.22 | 2.28 | -3.95 | 3.42 | 1 | 6,437.24 | 3,075.32 |
| r_pid__mu3[R_XJD47234,Intercept] | -0.22 | -0.15 | 2.42 | -4.27 | 3.53 | 1 | 6,369.73 | 2,838.52 |
| r_pid__mu3[R_XKE91696,Intercept] | -0.24 | -0.19 | 2.29 | -4.06 | 3.44 | 1 | 7,245.05 | 3,468.03 |
| r_pid__mu3[R_XNA94006,Intercept] | -0.32 | -0.26 | 2.35 | -4.26 | 3.42 | 1 | 5,497.07 | 2,766.28 |
| r_pid__mu3[R_XTF49093,Intercept] | -0.25 | -0.18 | 2.32 | -4.14 | 3.36 | 1 | 6,523.65 | 3,216.37 |
| r_pid__mu3[R_XYB26906,Intercept] | -0.18 | -0.08 | 2.28 | -4.03 | 3.38 | 1 | 6,457.60 | 2,933.62 |
| r_pid__mu3[R_XYE30129,Intercept] | -0.33 | -0.26 | 2.31 | -4.21 | 3.27 | 1 | 5,653.24 | 3,395.86 |
| r_pid__mu3[R_YBP87571,Intercept] | -0.23 | -0.13 | 2.27 | -4.04 | 3.36 | 1 | 6,268.34 | 3,544.58 |
| r_pid__mu3[R_YGA78661,Intercept] | -0.25 | -0.16 | 2.29 | -4.2 | 3.29 | 1 | 5,770.43 | 3,193.10 |
| r_pid__mu3[R_YSW98879,Intercept] | -0.2 | -0.15 | 2.29 | -4.14 | 3.44 | 1 | 6,584.30 | 3,266.41 |
| r_pid__mu3[R_YYH17600,Intercept] | -0.34 | -0.26 | 2.29 | -4.23 | 3.3 | 1 | 6,057.28 | 3,082.86 |
