# Supplementary material for: Treatment preferences among people at risk of developing tuberculosis: A discrete choice experiment
Source: PLOS Glob Public Health. 2024 Jul 19;4(7):e0002804. doi: 10.1371/journal.pgph.0002804 (PMC11259259; doi:10.1371/journal.pgph.0002804)
Supplement: S3 Table — (DOCX) [file pgph.0002804.s005.docx]

**Supplemental Table S3:** Treatment preferences among those with consistent responses N=103

| **Attribute** | **Level** | **Odds Ratio (95%CI)** | ***P-value** |
| --- | --- | --- | --- |
| Number of tablets per dose | 2 | 1 |  |
|  | 4 | 0.82 (0.60, 1.11) | 0.2 |
|  | 6 | 0.67 (0.41, 1.10) | 0.11 |
| Duration of treatment (months) | | 1.44 (1.28, 1.61) | <0.001 |
| Reduction in risk of developing disease after completing treatment | 50% | 1 |  |
|  | 65% | 0.85 (0.60, 1.21) | 0.37 |
|  | 80% | 3.20 (2.13, 4.79) | <0.001 |
|  | 95% | 1.67 (1.08, 2.59) | 0.021 |
| Likelihood of infecting others with TB disease | Does not stop transmission | 1 |  |
|  | reduces the chances of transmission by half | 10.36 (7.15, 15.02) | <0.001 |
|  | Completely stops transmission | 9.63 (6.68, 13.88) | <0.001 |
| Adverse effects | None | 1 |  |
|  | Minimal | 0.33 (0.23, 0.49) | <0.001 |
|  | Mild | 0.19 (0.13, 0.28) | <0.001 |
|  | Moderate | 0.18 (0.11, 0.29) | <0.001 |
| Follow up | No follow up | 1 |  |
|  | Once a month | 2.53 (1.88, 3.39) | <0.001 |
|  | 3 times a month | 1.23 (0.9, 1.66) | 0.19 |
| Annual travel cost | No cost | 1 |  |
|  | USD$2.95 | 1.90 (0.89, 4.05) | 0.099 |
|  | USD$7.38 | 3.59 (2.05, 6.30) | <0.001 |
| Opting out of treatment | | 0.15 (0.05, 0.44) | <0.001 |

CI: Confidence Interval

USD$1.00 = MWK812.51 as of 14/09/22(26)

**Sensitivity Analysis**

Supplemental table S3 shows the results of the multinomial logit model, however excluding the individuals who did not choose the same treatment option during the repeated task. As compared to the previous analysis illustrated in table 3, the results show slight changes in OR. Of note the OR for choosing 6 tablets per dose as compared to those with 2 tablets per dose increased from 0.60 to 0.67 with p-value increasing from p=0.02 to p=0.11. Another notable change is that the coefficients for the “likelihood of infecting others with TB disease” attribute levels had increased and represented the greatest change in magnitude as compared to the other attributes. With regards to the other attributes, there were slight changes in the magnitude of coefficients and their significance, however the interpretation of results remains the same.
