# Supplementary material for: Treatment preferences among people at risk of developing tuberculosis: A discrete choice experiment
Source: PLOS Glob Public Health. 2024 Jul 19;4(7):e0002804. doi: 10.1371/journal.pgph.0002804 (PMC11259259; doi:10.1371/journal.pgph.0002804)
Supplement: S1 Appendix — (PDF) [file pgph.0002804.s006.pdf]

RADIO+ TB: DISCRETE CHOICE EXPERIMENT QUESTIONNAIRE (SET ONE)

Scenario 1: **If you had a positive test result which means your risk of developing TB disease over the next 12 months is 30%, which treatment would you prefer?**

	Treatment A	Treatment B	No Treatment
The amount of time you would have to be on treatment if you were at risk of developing TB disease.	**Three (3) months of tablets** 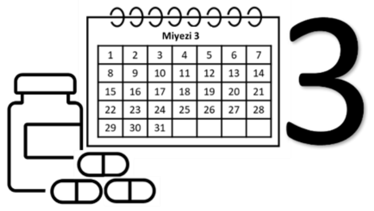	**Four (4) months of tablets** 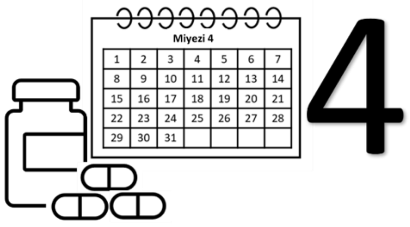	**No treatment** 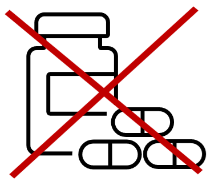
How many tablets you would have to take per dose.	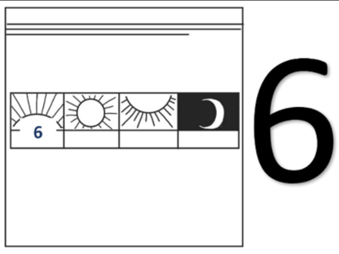	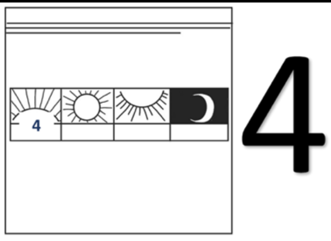	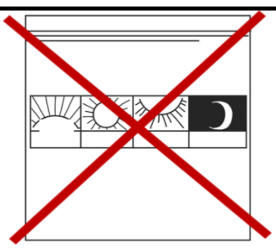
The reduction in the risk of you being unwell with TB disease, after completing treatment.	**50%** Chiopsezo cha matenda a TB mukamaliza kumwa mankhwala chitsika kuchoka pa 30% kufika pa 15% 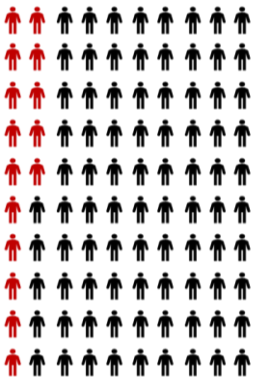	**65%** Chiopsezo cha matenda a TB mukamaliza kumwa mankhwala chitsika kuchoka pa 30% kufika pa 11% 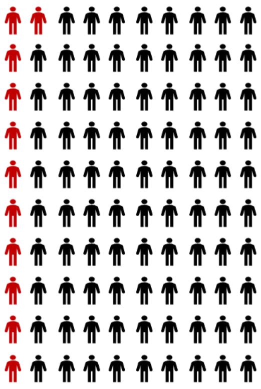	**0%** Chiopsezo chikhalabe pa 30% 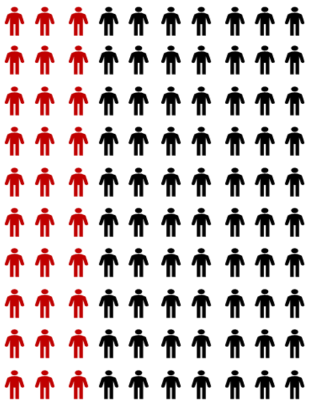
Whether you can still infect others after completing treatment.	**Will completely stop you from passing TB on to others.** 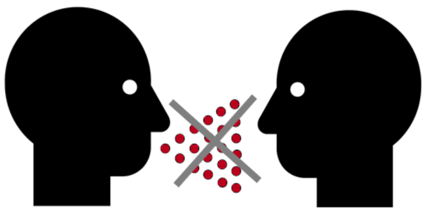	**Will completely stop you from passing TB on to others.** 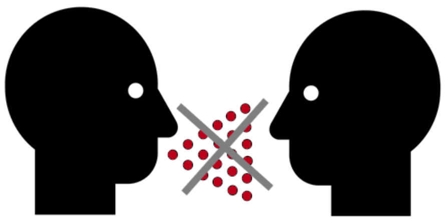	**Does not stop you from passing TB on to others.** 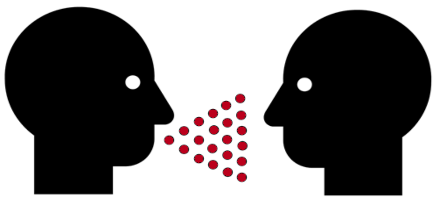
Possible harmful side-effects from the treatment.	Mild side effects present each day- may make you feel like not enjoying socializing but able to work. 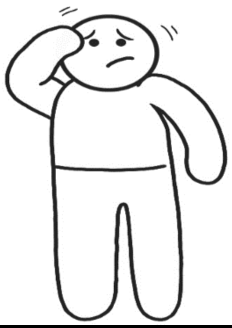	Minimal side effects- generally not noticeable such as brief feeling of sickness, 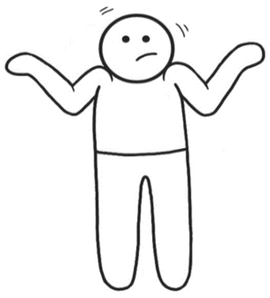	No side effects 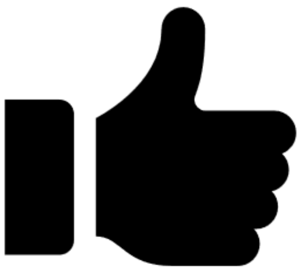
How often you would have to be checked on by health workers.	**None** 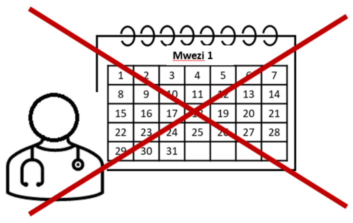	**Three (3) times a month** 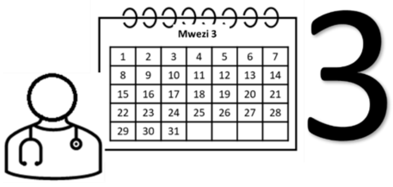	None 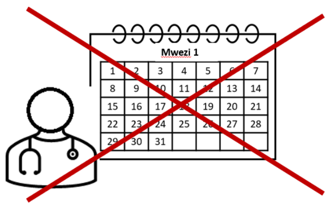
How much you would spend travelling from your home to a healthcare facility to access care in a year.	**K2,400** 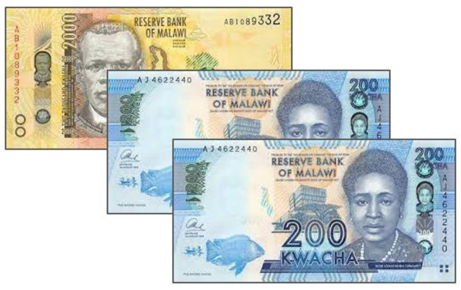	**K6,000** 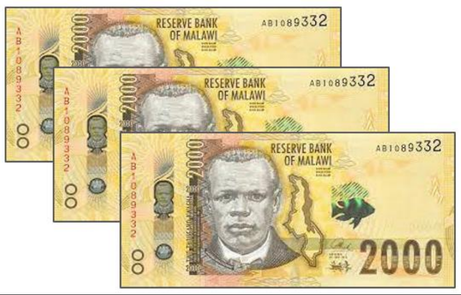	**K0** 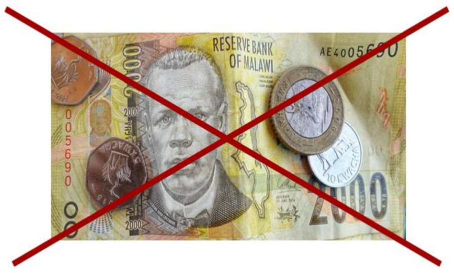
**Please choose which treatment you prefer**			

Scenario 2: **If you had a positive test result which means your risk of developing TB disease over the next 12 months is 10%, which treatment would you prefer?**

	Treatment A	Treatment B	No Treatment
The amount of time you would have to be on treatment if you were at risk of developing TB disease.	Two (2) months of tablets 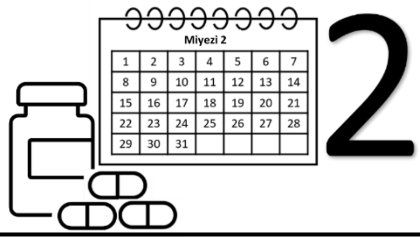	Three (3) months of tablets 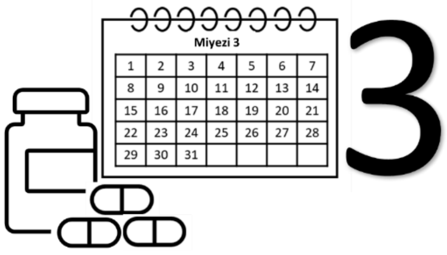	No treatment 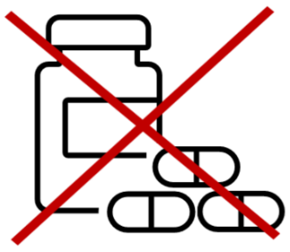
How many tablets you would have to take per dose.	4 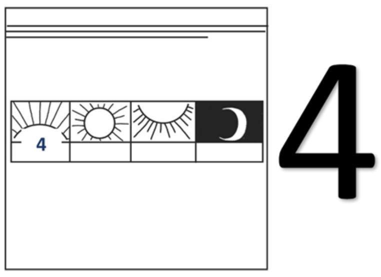	2 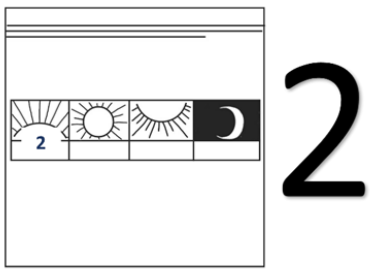	0 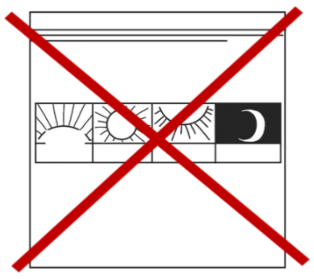
The reduction in the risk of you being unwell with TB disease, after completing treatment.	80% Chiopsezo cha matenda a TB mukamaliza kumwa mankhwala chitsika kuchoka pa 10% kufika pa 2% 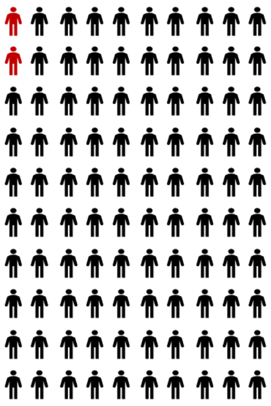	95% Chiopsezo cha matenda a TB mukamaliza kumwa mankhwala chitsika kuchoka pa 10% kufik pa 1% 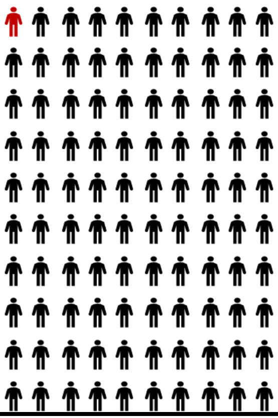	0% Chiopsezo chikhalabe pa 10% 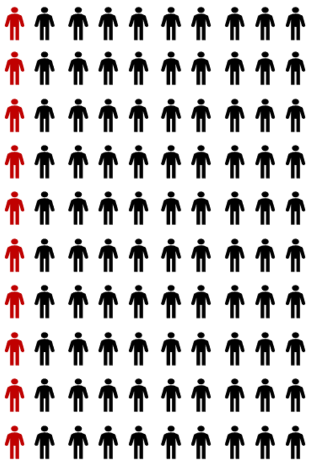
Whether you can still infect others after completing treatment.	Reduces the chances of passing TB on to others by half 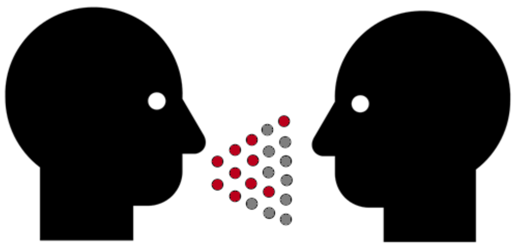	Does not stop you from passing TB on to others. 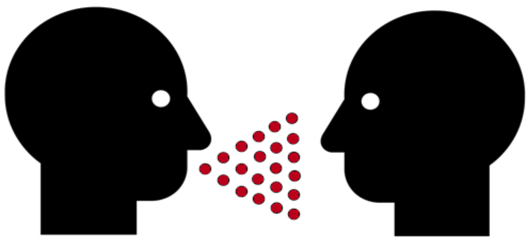	Does not stop you from passing TB on to others. 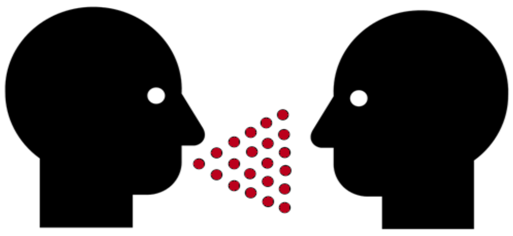
Possible harmful side-effects from the treatment.	No side effects 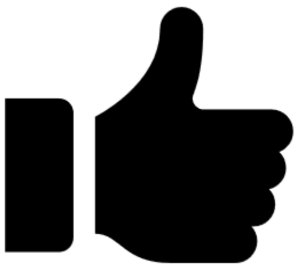	Mild side effects present each day- may make you feel like not enjoying socializing but able to work. 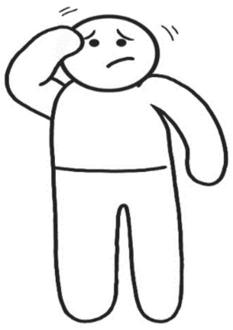	No side effects 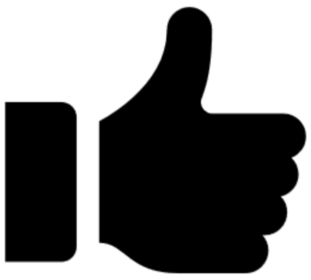
How often you would have to be checked on by health workers.	None 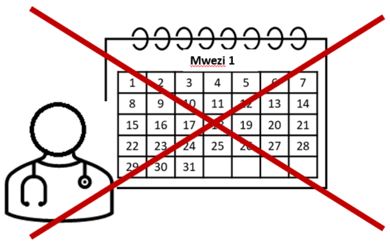	None 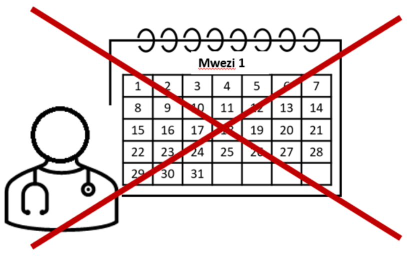	None 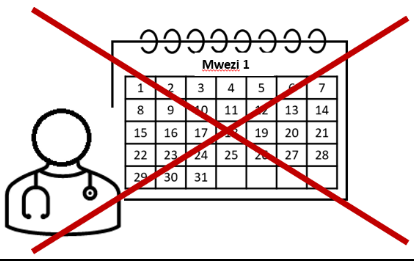
How much you would spend travelling from your home to a healthcare facility to access care in a year.	K0 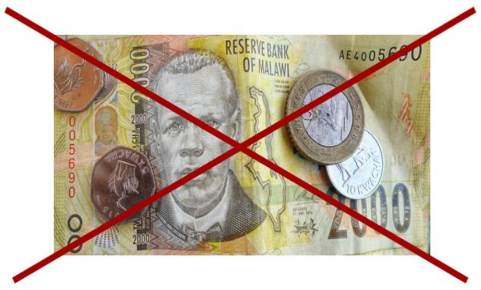	K6,000 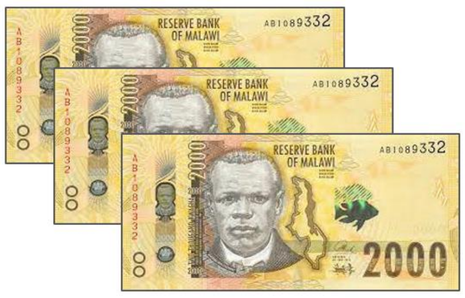	K0 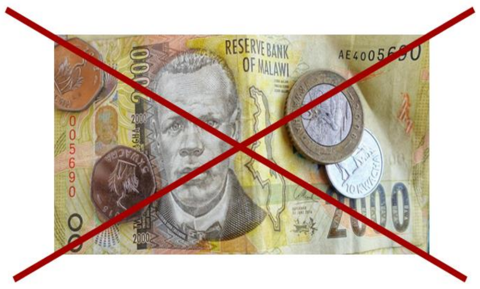
Please choose which treatment you prefer			

Scenario 3: If you had a positive test result which means your risk of developing TB disease over the next 12 months is 50%, which treatment would you prefer?

	Treatment A	Treatment B	No Treatment
The amount of time you would have to be on treatment if you were at risk of developing TB disease.	Four (4) months of tablets 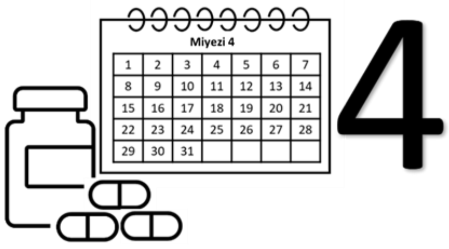	Six (6) months of tablets 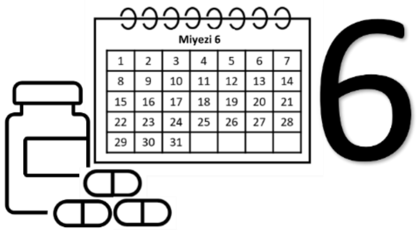	No treatment 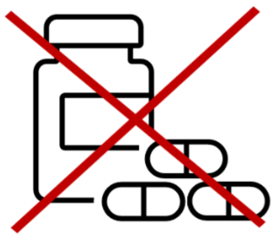
How many tablets you would have to take per dose.	2 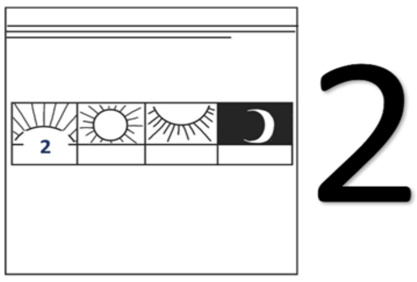	2 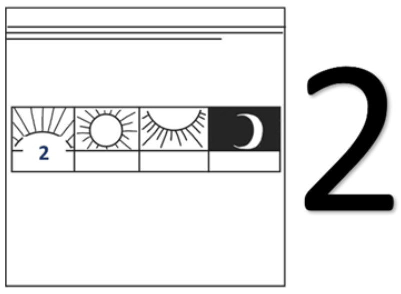	0 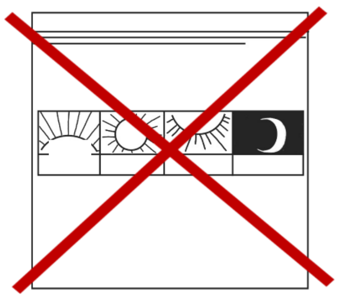
The reduction in the risk of you being unwell with TB disease, after completing treatment.	50% Chiopsezo cha matenda a TB mukamaliza kumwa mankhwala chitsika kuchoka pa 50% kufika pa 25% 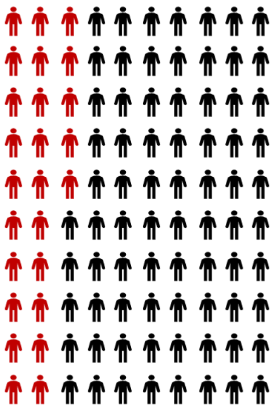	80% Chiopsezo cha matenda a TB mukamaliza kumwa mankhwala chitsika kuchoka pa 50% kufika pa 10% 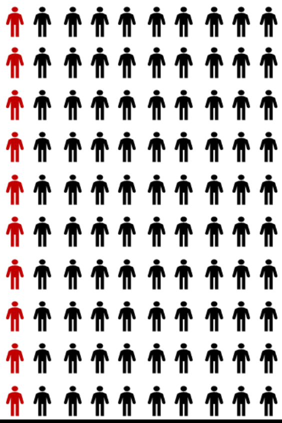	0% Chiopsezo chikhalabe pa 50% 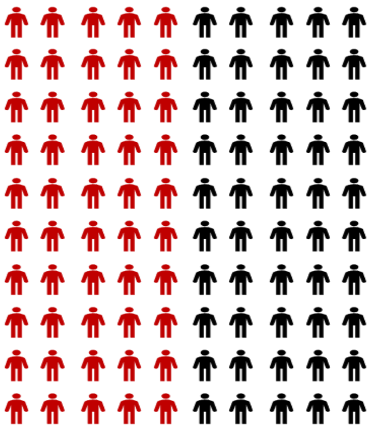
Whether you can still infect others after completing treatment.	Does not stop you from passing TB on to others. 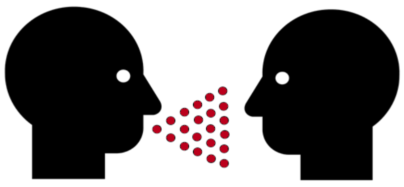	Does not stop you from passing TB on to others. 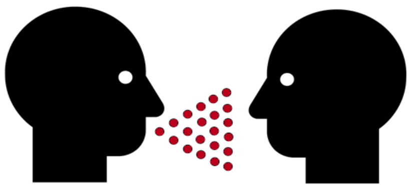	Does not stop you from passing TB on to others. 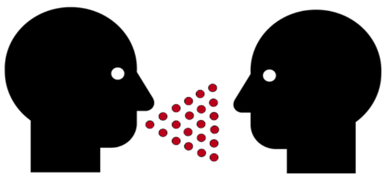
Possible harmful side-effects from the treatment.	Moderate side effects- bad enough to stop work and see the nurse/doctor for help 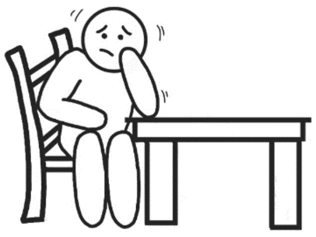	No side effects 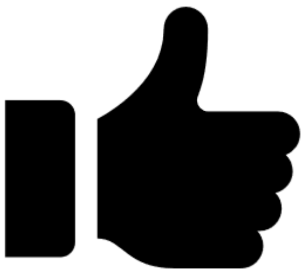	No side effects 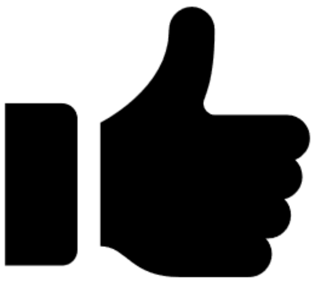
How often you would have to be checked on by health workers.	None 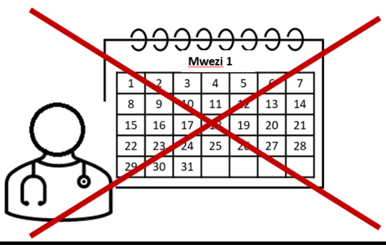	Once (1) a month 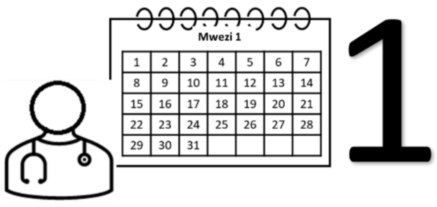	None 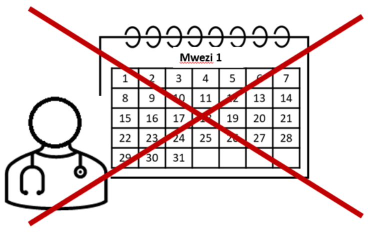
How much you would spend travelling from your home to a healthcare facility to access care in a year.	K6000 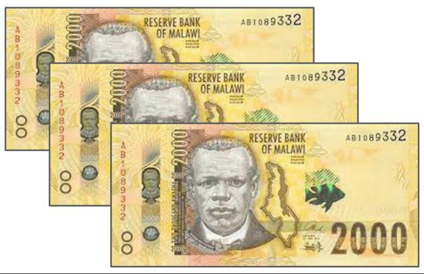	K0 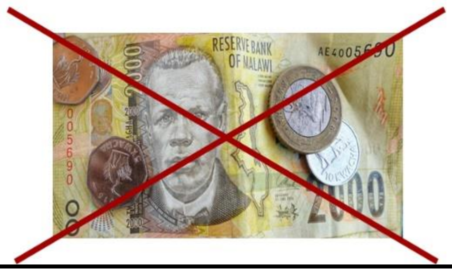	K0 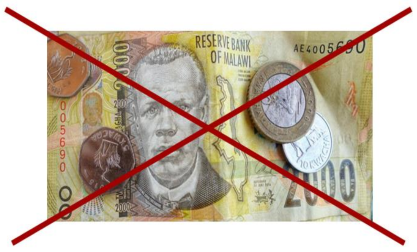
Please choose which treatment you prefer			

Scenario 4: **If you had a positive test result which means your risk of developing TB disease over the next 12 months is 10%, which treatment would you prefer?**

	Treatment A	Treatment B	No Treatment
The amount of time you would have to be on treatment if you were at risk of developing TB disease.	Six (6) months of tablets 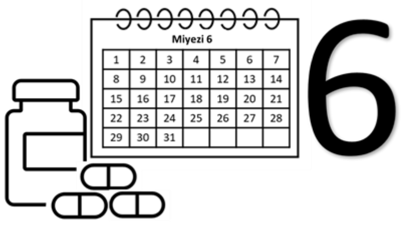	Four (4) months of tablets 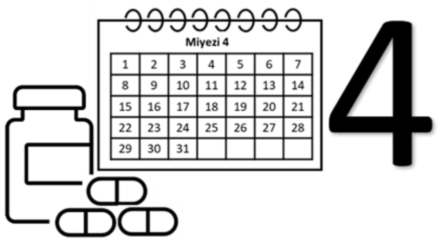	No treatment 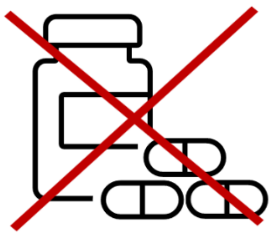
How many tablets you would have to take per dose.	4 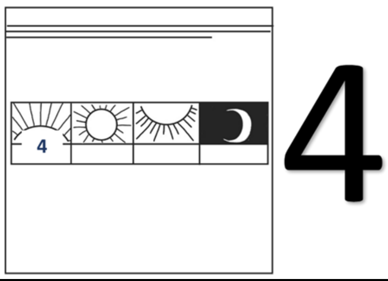	6 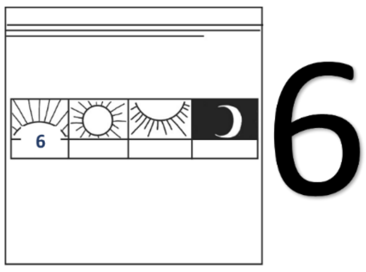	0 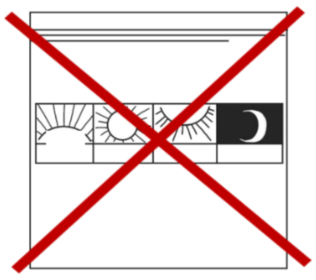
The reduction in the risk of you being unwell with TB disease, after completing treatment.	95% Chiopsezo cha matenda a TB mukamaliza kumwa mankhwala chitsika kuchoka pa 10% kufika pa 1% 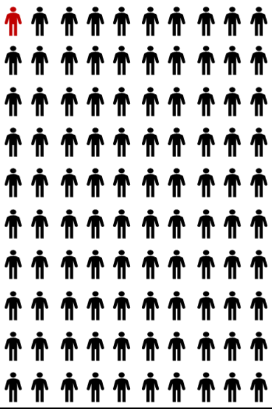	65% Chiopsezo cha matenda a TB mukamaliza kumwa mankhwala chitsika kuchoka pa 10% kufika pa 4% 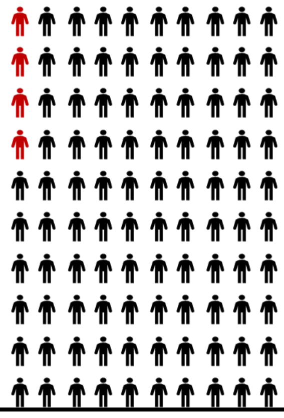	0% Chiopsezo chikhalabe pa 10% 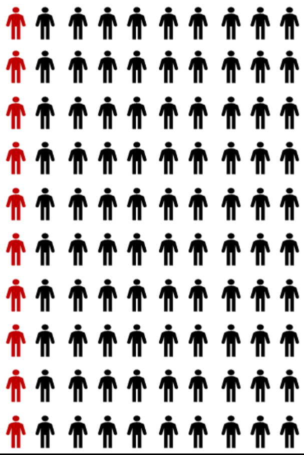
Whether you can still infect others after completing treatment.	Will completely stop you from passing TB on to others. 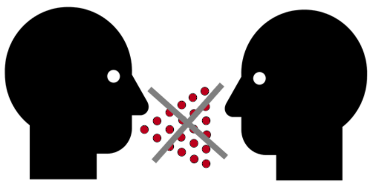	Does not stop you from passing TB on to others. 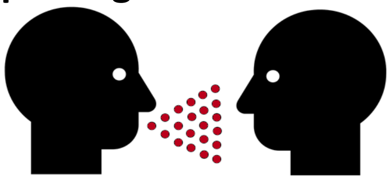	Does not stop you from passing TB on to others. 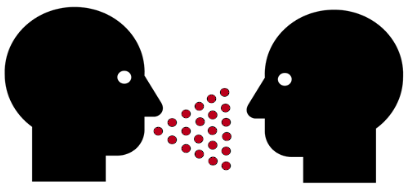
Possible harmful side-effects from the treatment.	Moderate side effects- bad enough to stop work and see the nurse/doctor for help 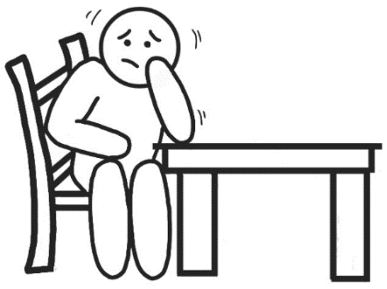	Moderate side effects- bad enough to stop work and see the nurse/doctor for help 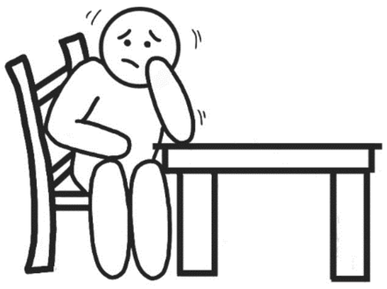	No side effects 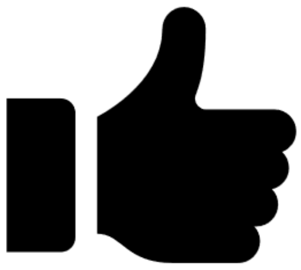
How often you would have to be checked on by health workers.	Once (1) a month 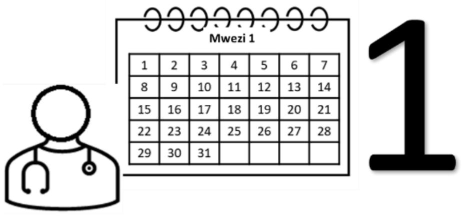	None 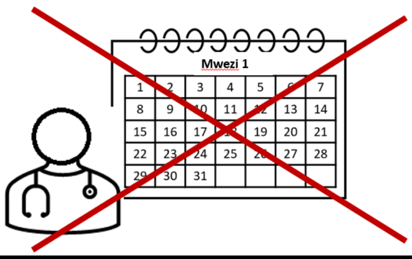	None 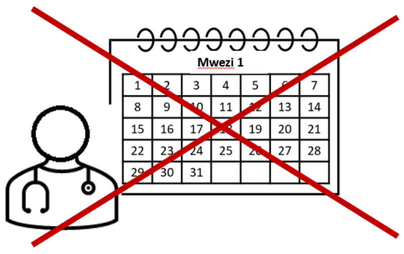
How much you would spend travelling from your home to a healthcare facility to access care in a year.	K6,000 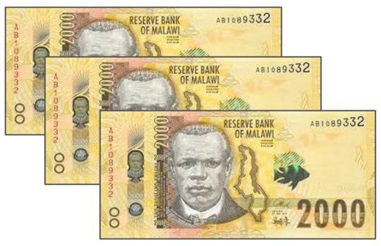	K2,400 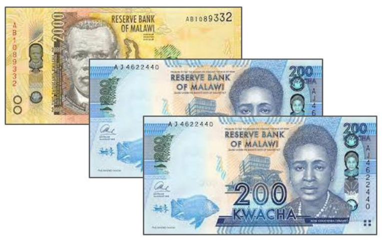	K0 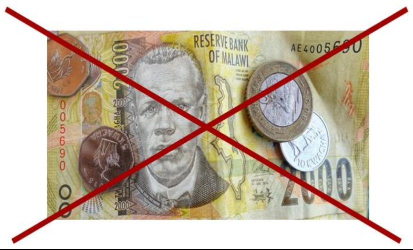
Please choose which treatment you prefer			

Scenario 5: **If you had a positive test result which means your risk of developing TB disease over the next 12 months is 10%, which treatment would you prefer?**

	Treatment A	Treatment B	No Treatment
The amount of time you would have to be on treatment if you were at risk of developing TB disease.	Five (5) months of tablets 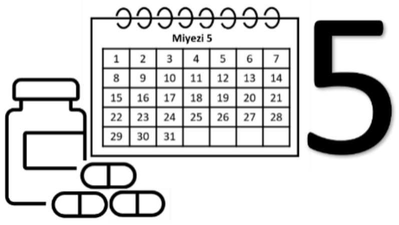	Five (5) months of tablets 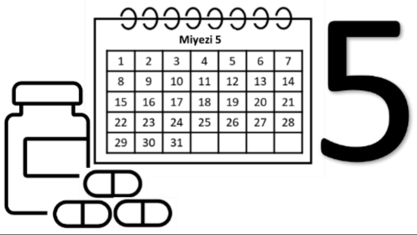	No treatment 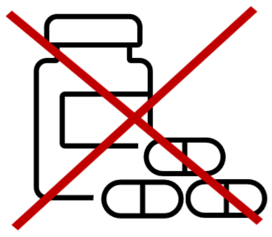
How many tablets you would have to take per dose.	6 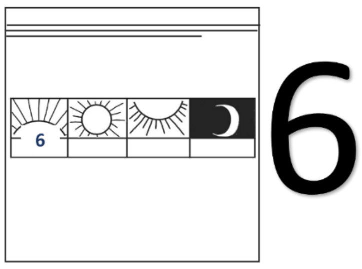	6 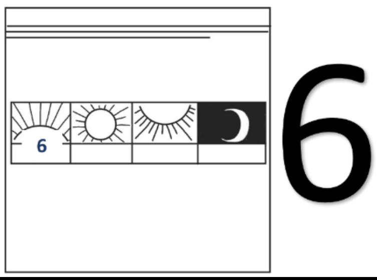	0 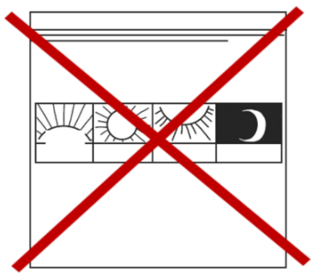
The reduction in the risk of you being unwell with TB disease, after completing treatment.	95% Chiopsezo cha matenda a TB mukamaliza kumwa mankhwala chitsika kuchoka pa 10% kufika pa 1% 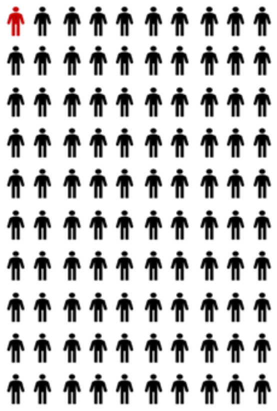	50% Chiopsezo cha matenda a TB mukamaliza kumwa mankhwala chitsika kuchoka pa 10% kufika pa 5% 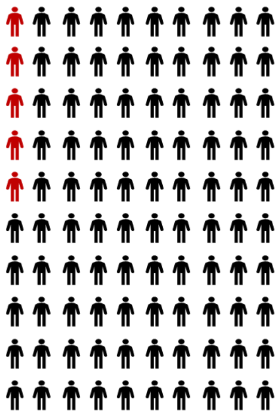	0% Chiopsezo chikhalabe pa 10% 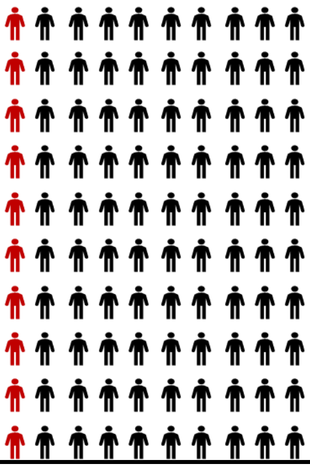
Whether you can still infect others after completing treatment.	Does not stop you from passing TB on to others. 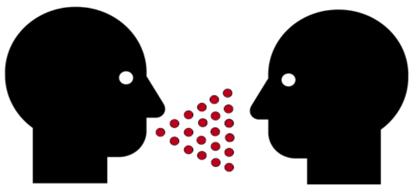	Will completely stop you from passing TB on to others. 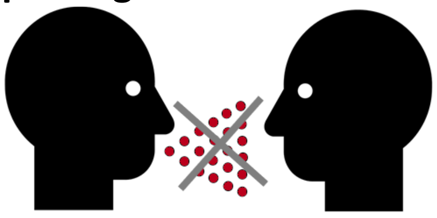	Does not stop you from passing TB on to others. 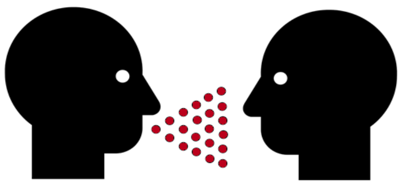
Possible harmful side-effects from the treatment.	Minimal side effects- generally not noticeable such as brief feeling of sickness. 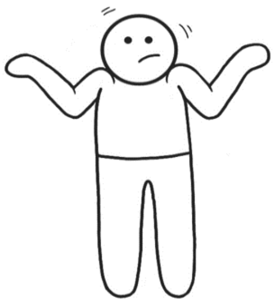	Mild side effects present each day- may make you feel like not enjoying socializing but able to work. 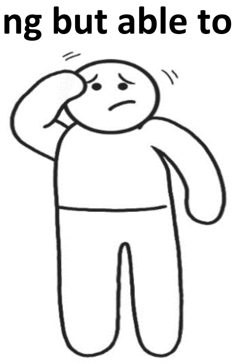	No side effects 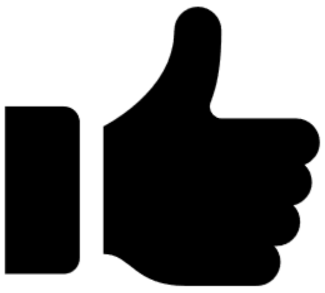
How often you would have to be checked on by health workers.	Three (3) times a month 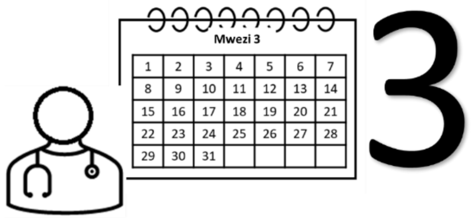	Three (3) times a month 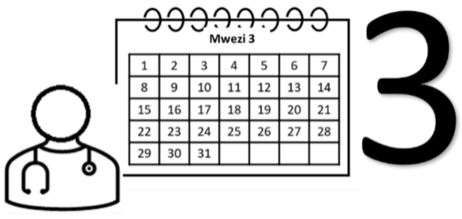	None 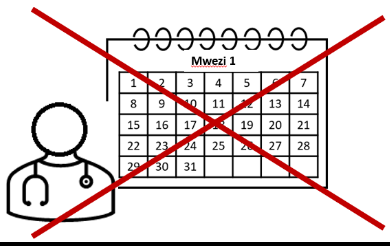
How much you would spend travelling from your home to a healthcare facility to access care in a year.	K2,400 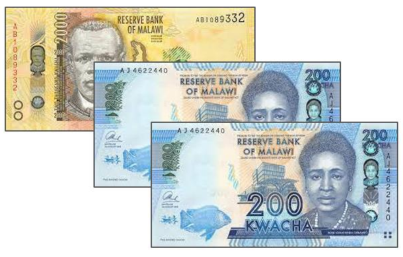	K6,000 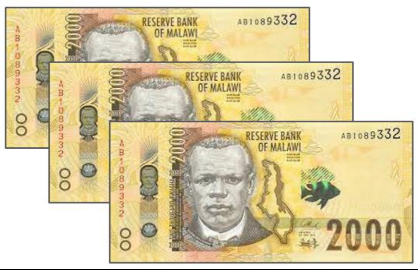	K0 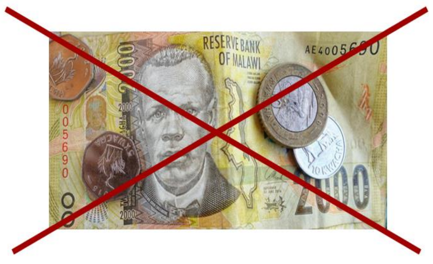
Please choose which treatment you prefer			

Scenario 6: If you had a positive test result which means your risk of developing TB disease over the next 12 months is 30%, which treatment would you prefer?

	Treatment A	Treatment B	No Treatment
The amount of time you would have to be on treatment if you were at risk of developing TB disease.	Three (3) months of tablets<br>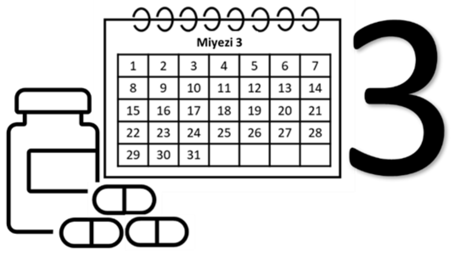	Three (3) months of tablets<br>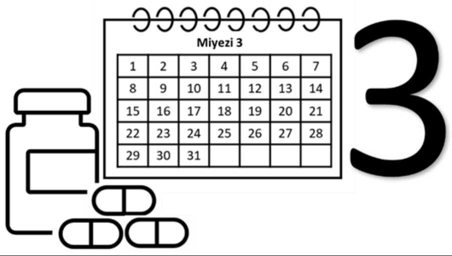	No treatment<br>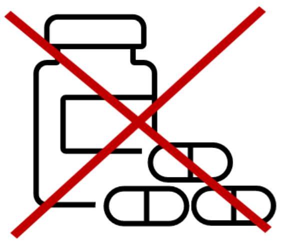
How many tablets you would have to take per dose.	2<br>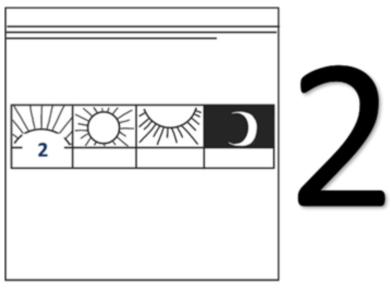	6<br>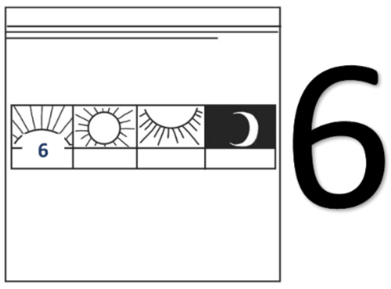	0<br>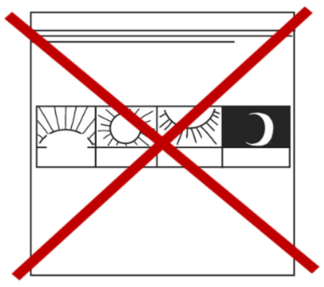
The reduction in the risk of you being unwell with TB disease, after completing treatment.	80%<br>Chiopsezo cha matenda a TB mukamaliza kumwa mankhwala chitsika kuchoka pa 30% kufika pa 6%<br>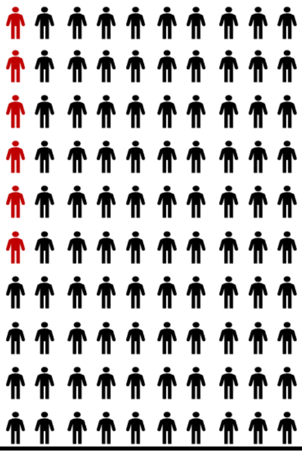	80%<br>Chiopsezo cha matenda a TB mukamaliza kumwa mankhwala chitsika kuchoka pa 30% kufika pa 6%<br>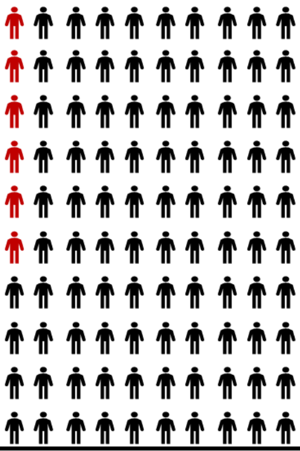	0%<br>Chiopsezo chikhalabe pa 30%<br>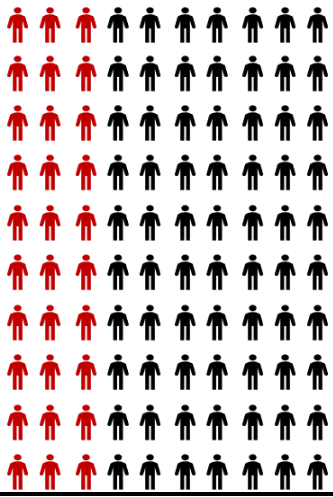
Whether you can still infect others after completing treatment.	Does not stop you from passing TB on to others.<br>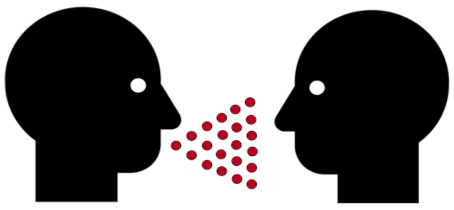	Reduces the chances of passing TB on to others by half<br>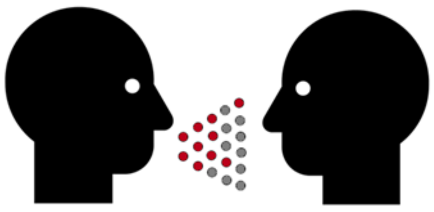	Does not stop you from passing TB on to others.<br>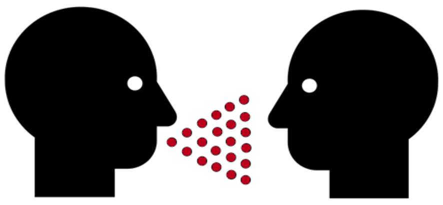
Possible harmful side-effects from the treatment.	Minimal side effects- generally not noticeable such as brief feeling of sickness.<br>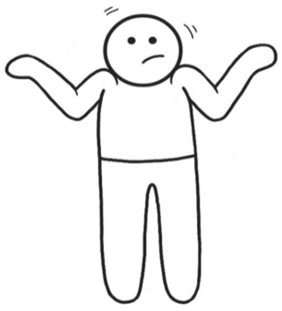	No side effects<br>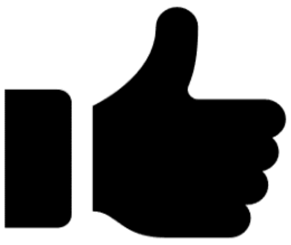	No side effects<br>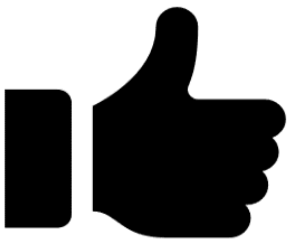
How often you would have to be checked on by health workers.	Three (3) times a month<br>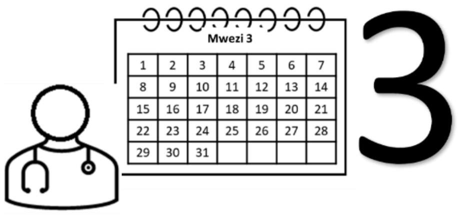	Once (1) a month<br>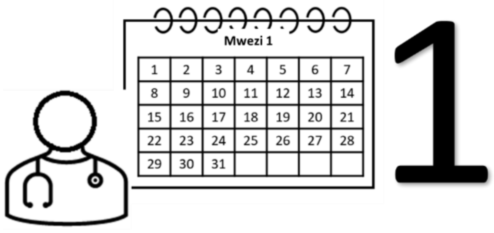	None<br>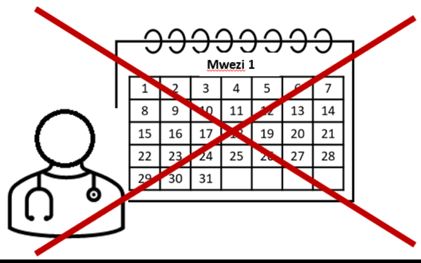
How much you would spend travelling from your home to a healthcare facility to access care in a year.	K0<br>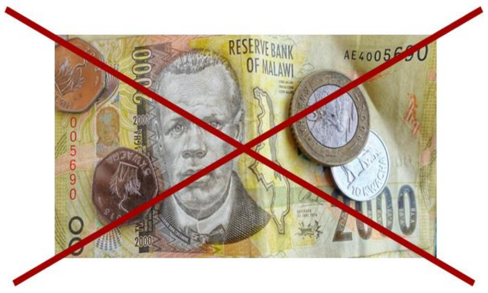	K2,400<br>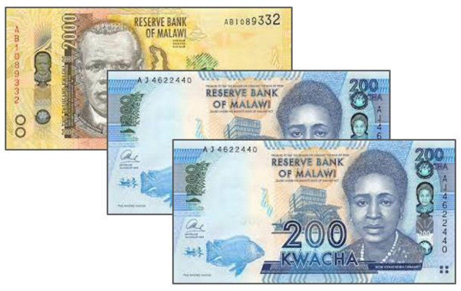	K0<br>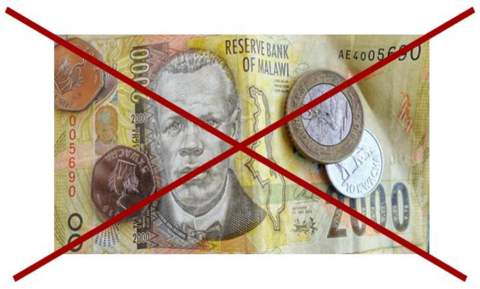
Please choose which treatment you prefer			

Scenario 7: **If you had a positive test result which means your risk of developing TB disease over the next 12 months is 50%, which treatment would you prefer?**

	Treatment A	Treatment B	No Treatment
The amount of time you would have to be on treatment if you were at risk of developing TB disease.	Four (4) months of tablets 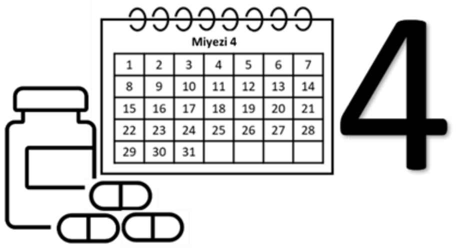	Two (2) months of tablets 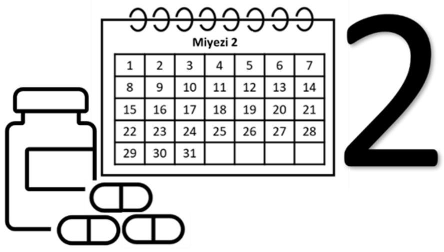	No treatment 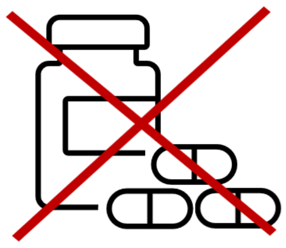
How many tablets you would have to take per dose.	6 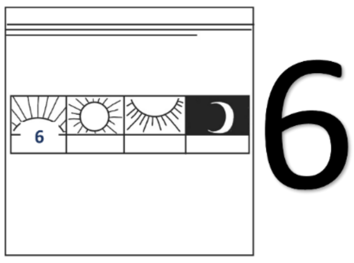	2 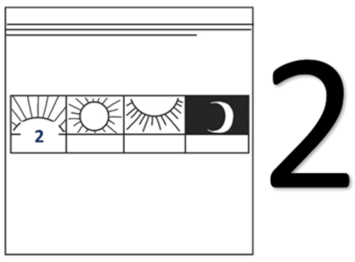	0 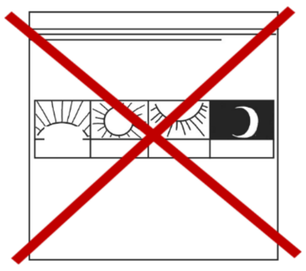
The reduction in the risk of you being unwell with TB disease, after completing treatment.	65% Chiopsezo cha matenda a TB mukamaliza kumwa mankhwala chitsika kuchoka pa 50% kufika pa 18% 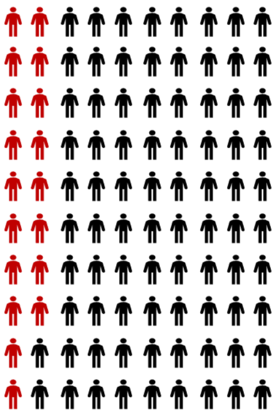	95% Chiopsezo cha matenda a TB mukamaliza kumwa mankhwala chitsika kuchoka pa 50% kufika pa 2% 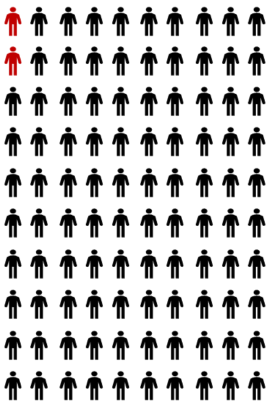	0% Chiopsezo chikhalabe pa 50% 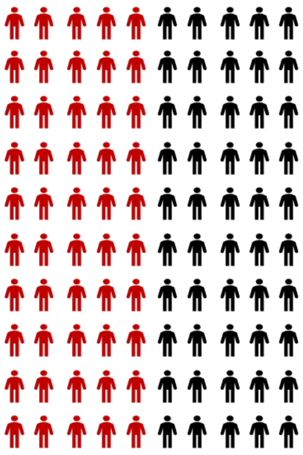
Whether you can still infect others after completing treatment.	Will completely stop you from passing TB on to others. 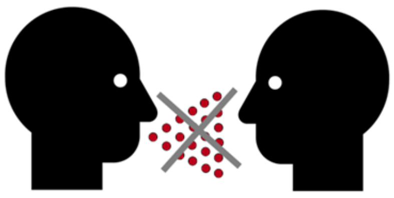	Will completely stop you from passing TB on to others. 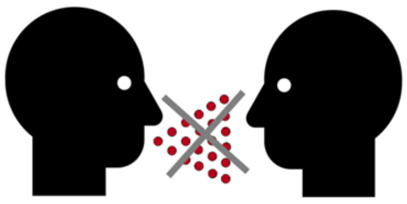	Does not stop you from passing TB on to others. 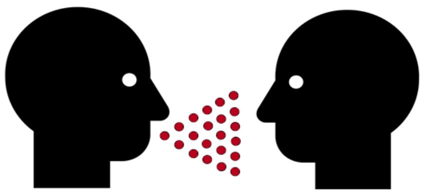
Possible harmful side-effects from the treatment.	Minimal side effects- generally not noticeable such as brief feeling of sickness. 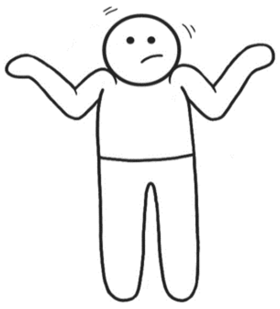	Moderate side effects- bad enough to stop work and see the nurse/doctor for help 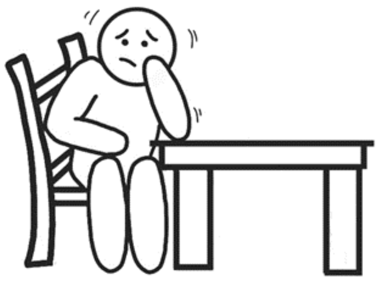	No side effects 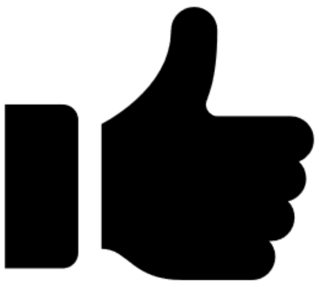
How often you would have to be checked on by health workers.	None 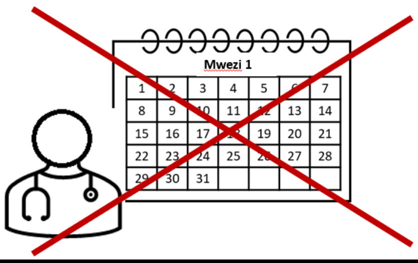	Three (3) times a month 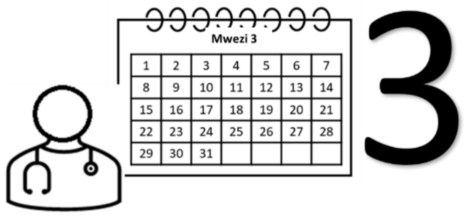	None 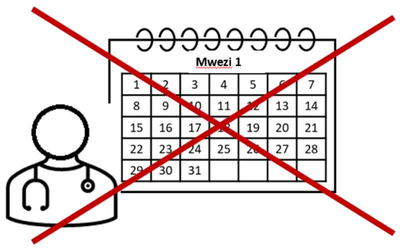
How much you would spend travelling from your home to a healthcare facility to access care in a year.	K6,000 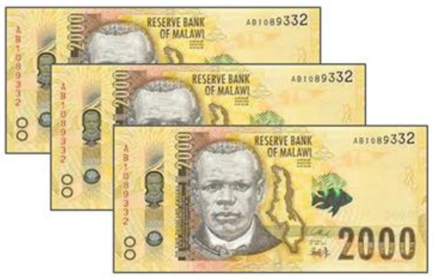	K2400 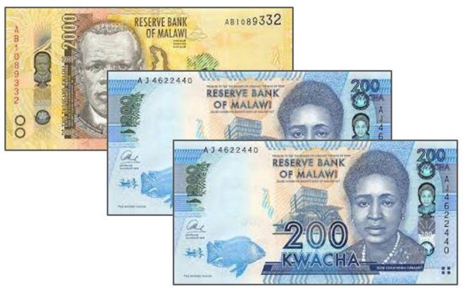	K0 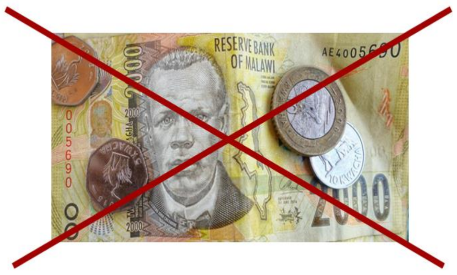
Please choose which treatment you prefer			

Scenario 8: **If you had a positive test result which means your risk of developing TB disease over the next 12 months is 50%, which treatment would you prefer?**

	Treatment A	Treatment B	No Treatment
The amount of time you would have to be on treatment if you were at risk of developing TB disease.	Six (6) months of tablets 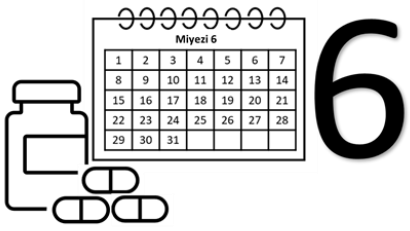	Five (5) months of tablets 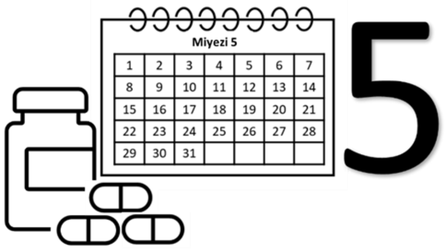	No treatment 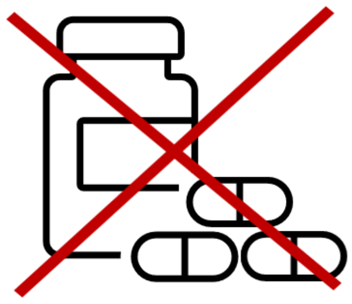
How many tablets you would have to take per dose.	2 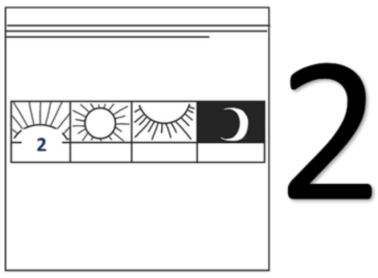	4 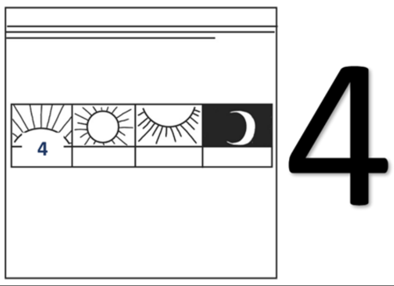	0 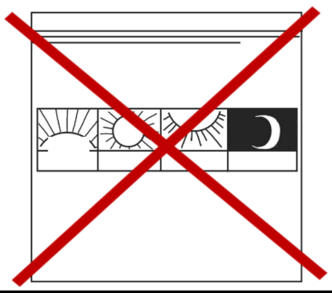
The reduction in the risk of you being unwell with TB disease, after completing treatment.	50% Chiopsezo cha matenda a TB mukamaliza kumwa mankhwala chitsika kuchoka pa 50% kufika pa 25% 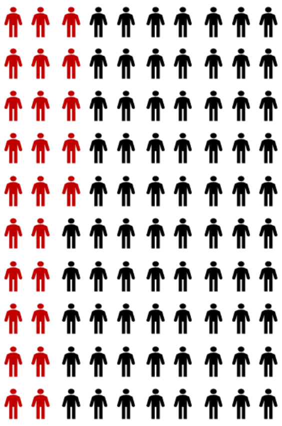	50% Chiopsezo cha matenda a TB mukamaliza kumwa mankhwala chitsika kuchoka pa 50% kufika pa 25% 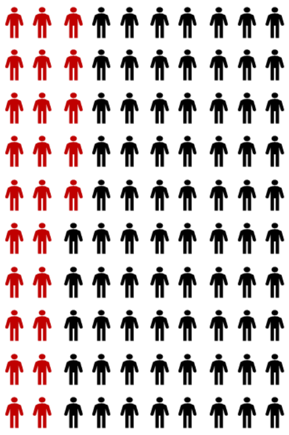	0% Chiopsezo chikhalabe pa 50% 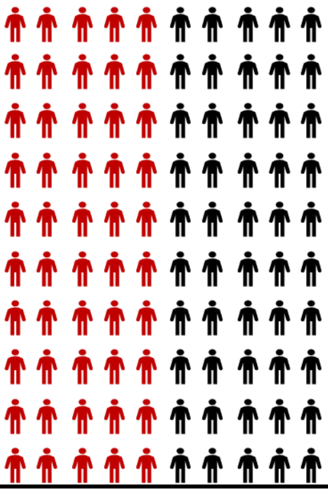
Whether you can still infect others after completing treatment.	Reduces the chances of passing TB on to others by half 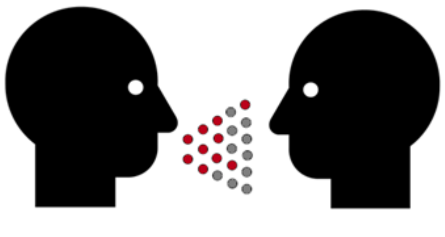	Does not stop you from passing TB on to others. 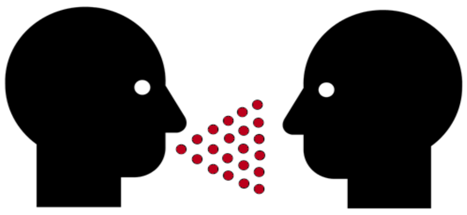	Does not stop you from passing TB on to others. 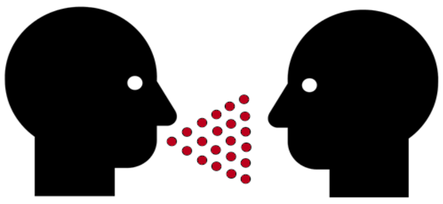
Possible harmful side-effects from the treatment.	No side effects 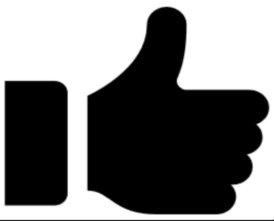	No side effects 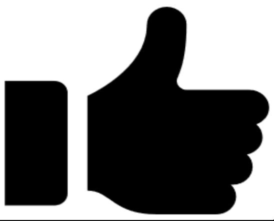	No side effects 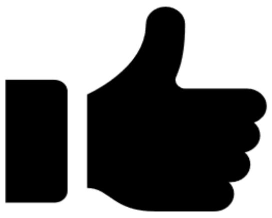
How often you would have to be checked on by health workers.	Three (3) times a month 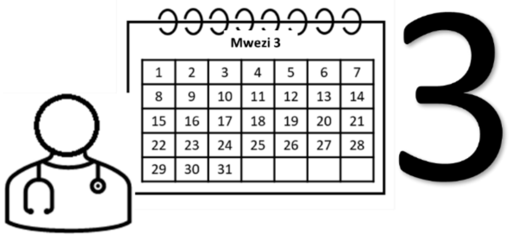	None 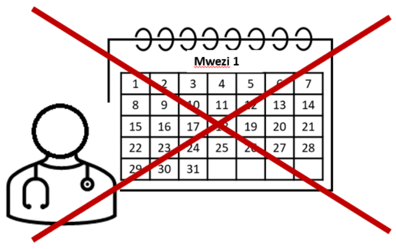	None 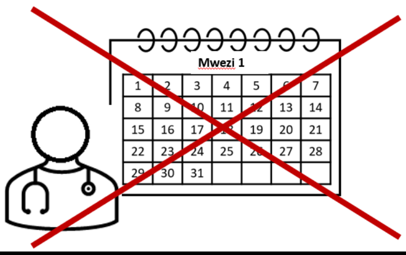
How much you would spend travelling from your home to a healthcare facility to access care in a year.	K0 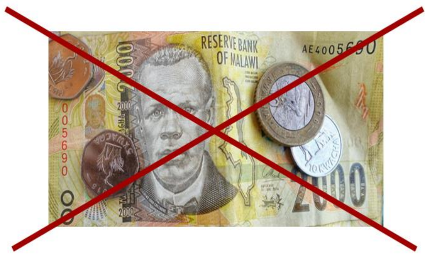	K0 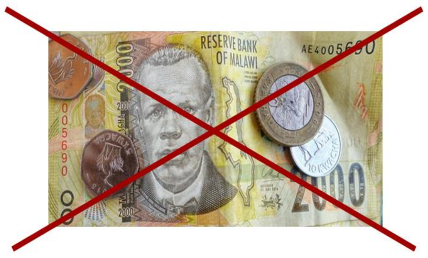	K0 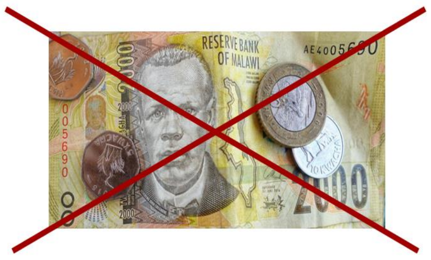
**Please choose which treatment you prefer**			

Scenario 9: **If you had a positive test result which means your risk of developing TB disease over the next 12 months is 30%, which treatment would you prefer?**

	Treatment A	Treatment B	No Treatment
The amount of time you would have to be on treatment if you were at risk of developing TB disease.	Five (5) months of tablets 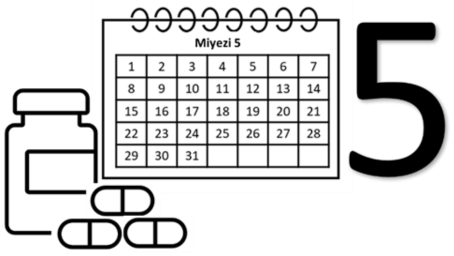	Two (2) months of tablets 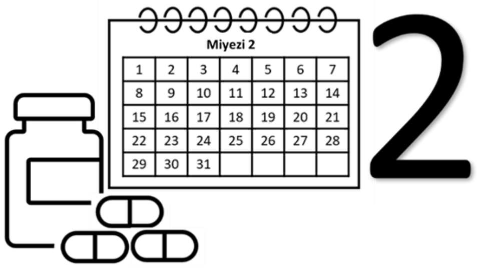	No treatment 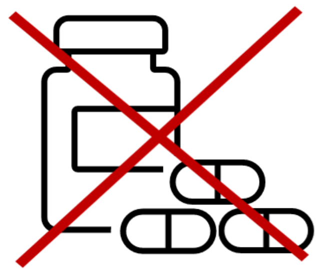
How many tablets you would have to take per dose.	4 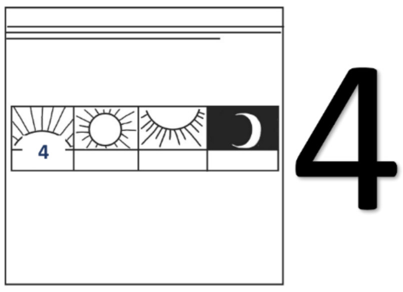	4 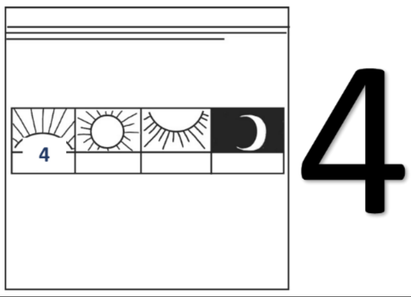	0 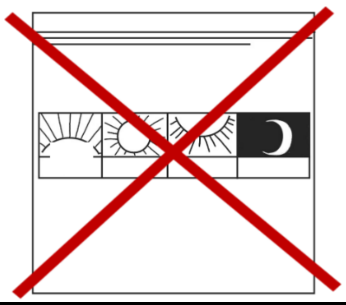
The reduction in the risk of you being unwell with TB disease, after completing treatment.	65% Chiopsezo cha matenda a TB mukamaliza kumwa mankhwala chitsika kuchoka pa 30% kufika pa 11% 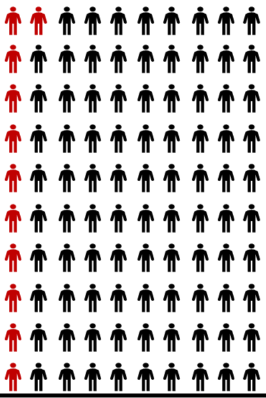	50% Chiopsezo cha matenda a TB mukamaliza kumwa mankhwala chitsika kuchoka pa 30% kufika pa 15% 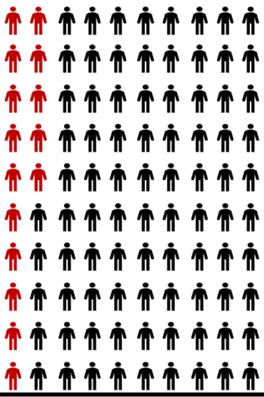	0% Chiopsezo chikhalabe pa 30% 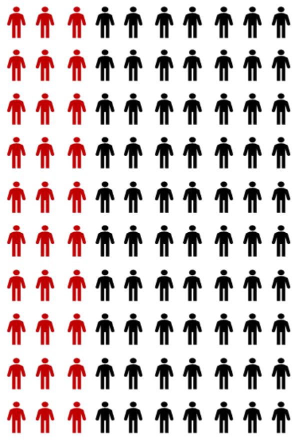
Whether you can still infect others after completing treatment.	Reduces the chances of passing TB on to others by half 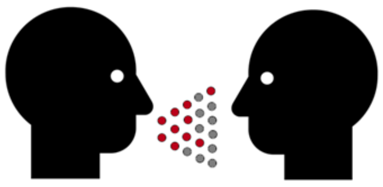	Reduces the chances of passing TB on to others by half 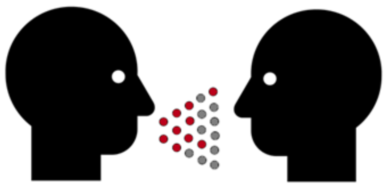	Does not stop you from passing TB on to others. 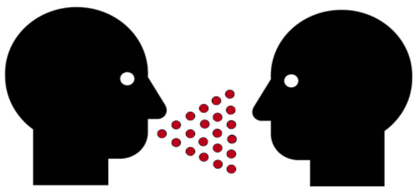
Possible harmful side-effects from the treatment.	Mild side effects present each day- may make you feel like not enjoying socializing but able to work. 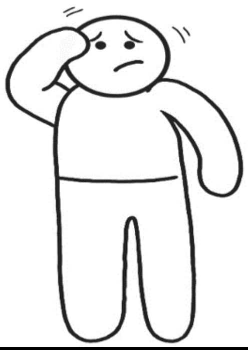	Minimal side effects- generally not noticeable such as brief feeling of sickness. 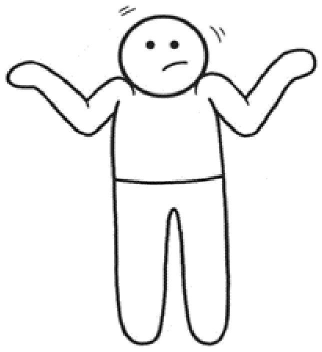	No side effects 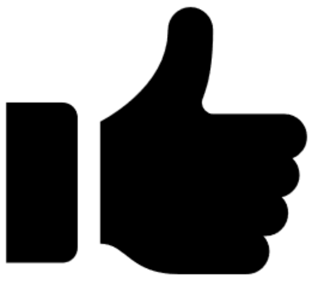
How often you would have to be checked on by health workers.	Once (1) a month 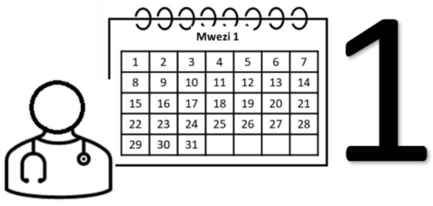	Once (1) a month 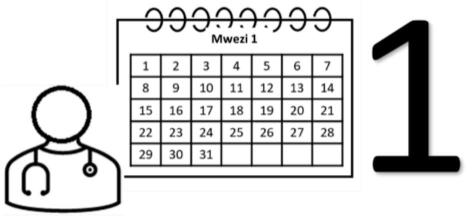	None 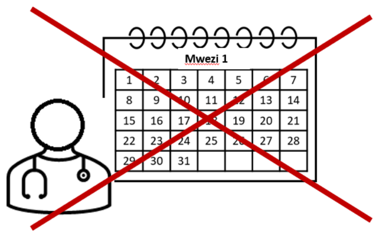
How much you would spend travelling from your home to a healthcare facility to access care in a year.	K0 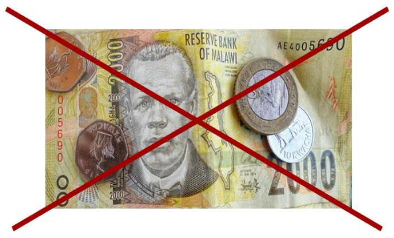	K0 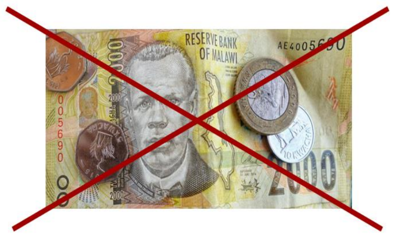	K0 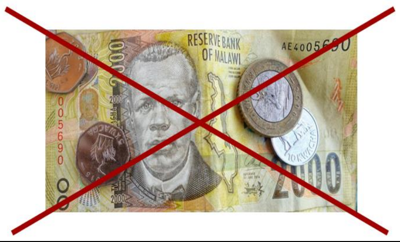
Please choose which treatment you prefer			

Scenario 10: If you had a positive test result which means your risk of developing TB disease over the next 12 months is 10%, which treatment would you prefer?

	Treatment A	Treatment B	No Treatment
The amount of time you would have to be on treatment if you were at risk of developing TB disease.	Two (2) months of tablets 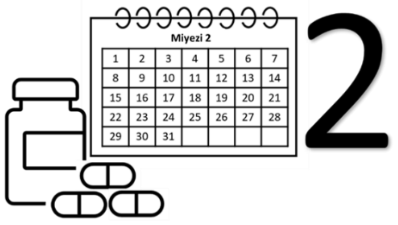	Six (6) months of tablets 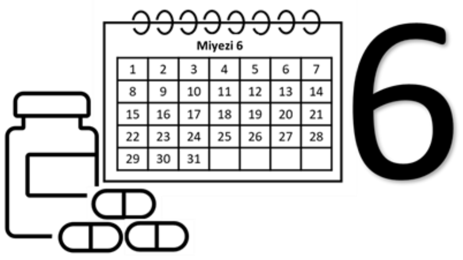	No treatment 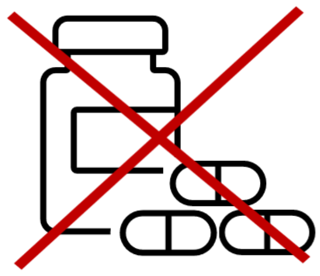
How many tablets you would have to take per dose.	2 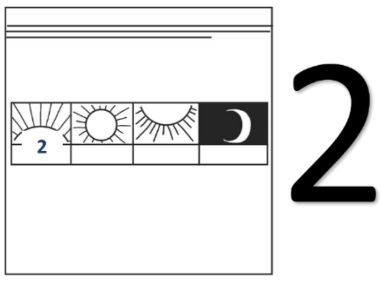	2 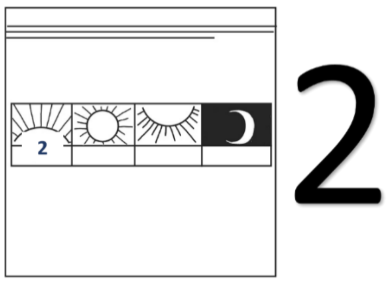	0 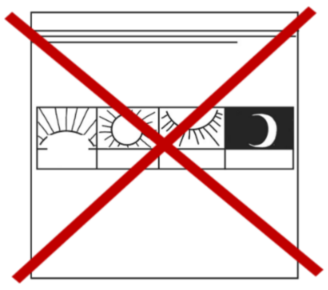
The reduction in the risk of you being unwell with TB disease, after completing treatment.	65%<br>Chiopsezo cha matenda a TB mukamaliza kumwa mankhwala chitsika kuchoka pa 10% kufika pa 4% 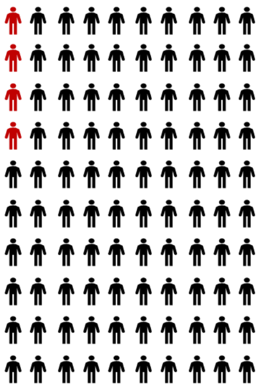	65%<br>Chiopsezo cha matenda a TB mukamaliza kumwa mankhwala chitsika kuchoka pa 10% kufika pa 4% 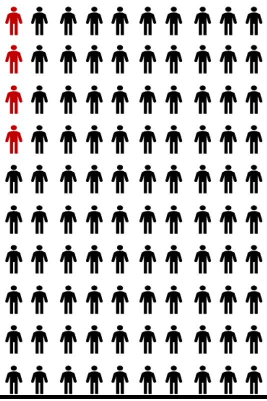	0%<br>Chiopsezo chikhalabe pa 10% 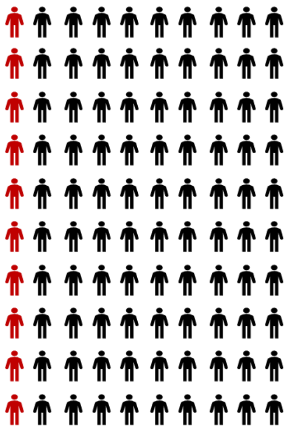
Whether you can still infect others after completing treatment.	Does not stop you from passing TB on to others. 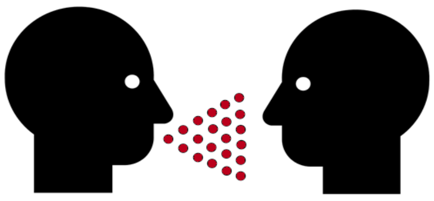	Reduces the chances of passing TB on to others by half 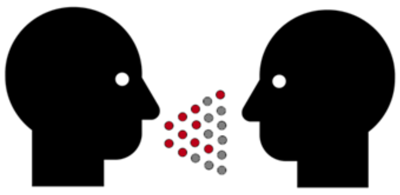	Does not stop you from passing TB on to others. 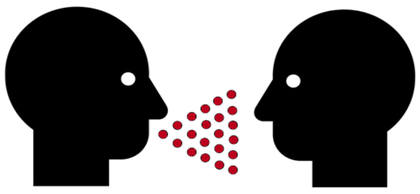
Possible harmful side-effects from the treatment.	No side effects 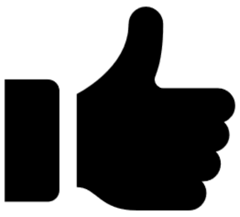	Minimal side effects- generally not noticeable such as brief feeling of sickness. 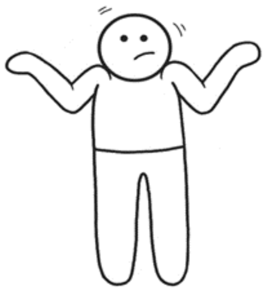	No side effects 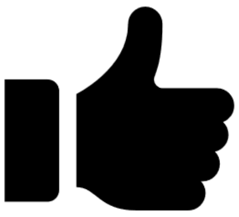
How often you would have to be checked on by health workers.	Once (1) a month 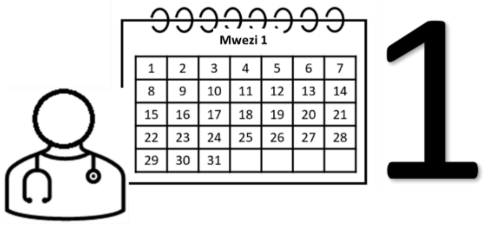	None 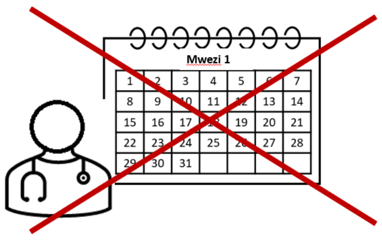	None 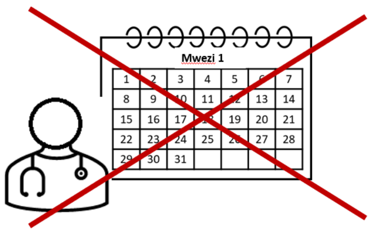
How much you would spend travelling from your home to a healthcare facility to access care in a year.	K2400 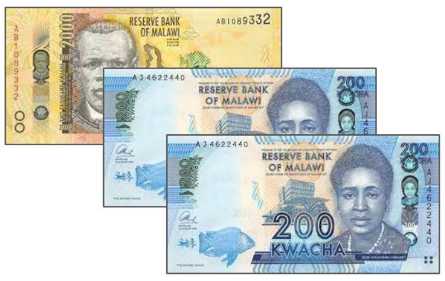	K0 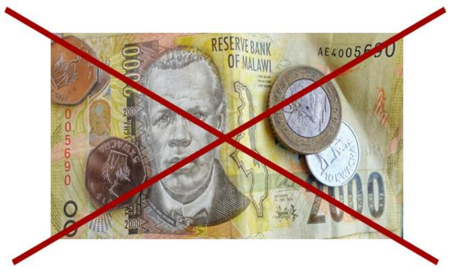	K0 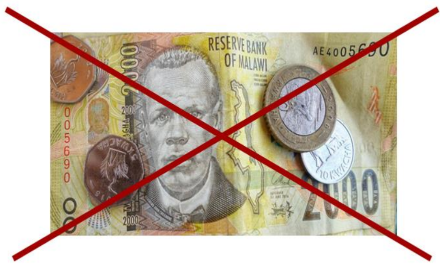
Please choose which treatment you prefer			

Scenario 11: If you had a positive test result which means your risk of developing TB disease over the next 12 months is 10%, which treatment would you prefer?

	Treatment A	Treatment B	No Treatment
The amount of time you would have to be on treatment if you were at risk of developing TB disease.	Five (5) months of tablets 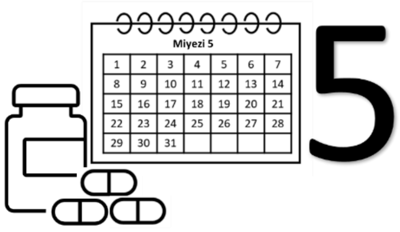	Five (5) months of tablets 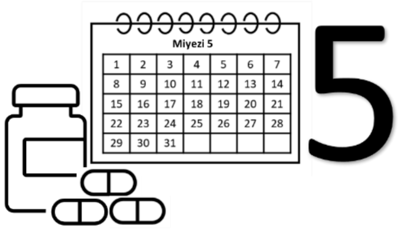	No treatment 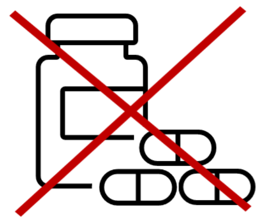
How many tablets you would have to take per dose.	6 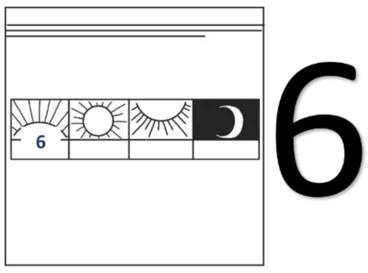	6 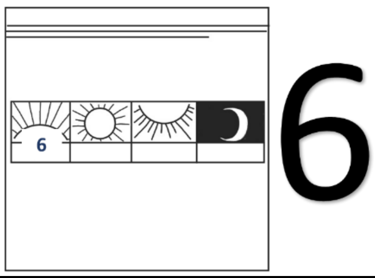	0 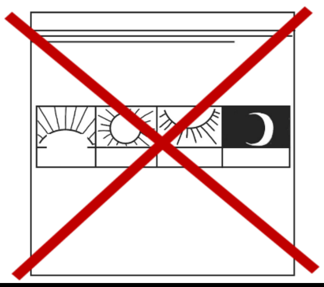
The reduction in the risk of you being unwell with TB disease, after completing treatment.	95%<br>Chiopsezo cha matenda a TB mukamaliza kumwa mankhwala chitsika kuchoka pa 10% kufika pa 1% 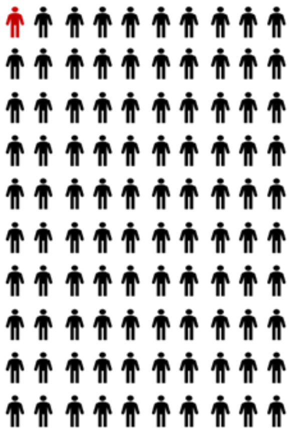	50%<br>Chiopsezo cha matenda a TB mukamaliza kumwa mankhwala chitsika kuchoka pa 10% kufika pa 5% 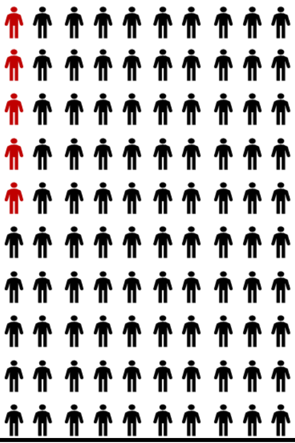	0%<br>Chiopsezo chikhalabe pa 10% 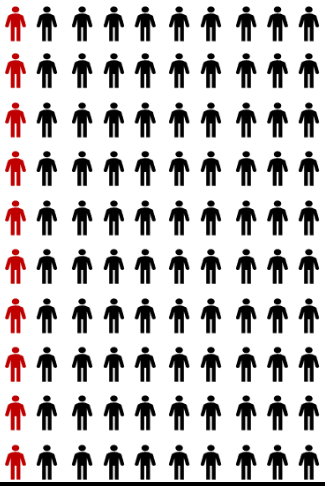
Whether you can still infect others after completing treatment.	Does not stop you from passing TB on to others. 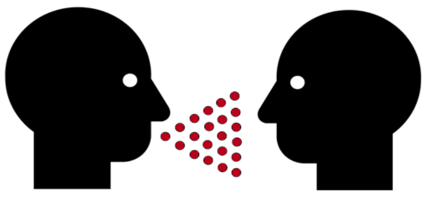	Will completely stop you from passing TB on to others. 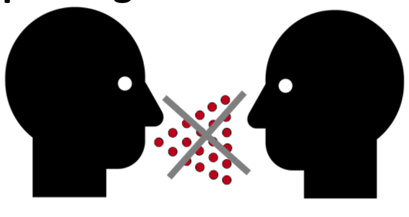	Does not stop you from passing TB on to others. 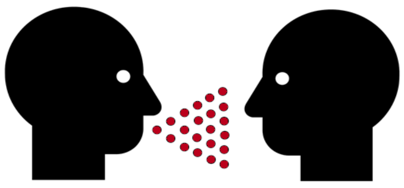
Possible harmful side-effects from the treatment.	Minimal side effects- generally not noticeable such as brief feeling of sickness. 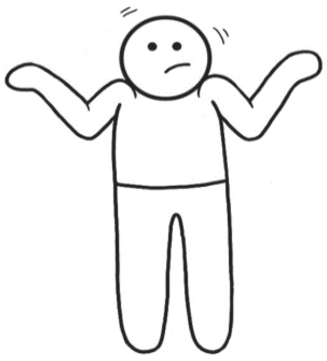	Mild side effects present each day- may make you feel like not enjoying socializing but able to work. 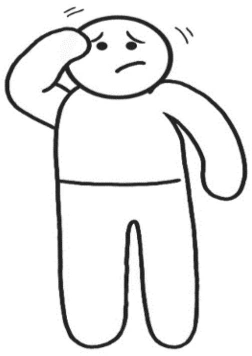	No side effects 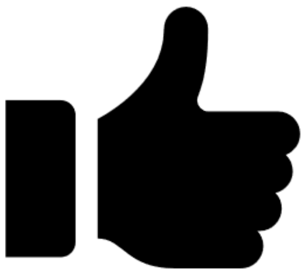
How often you would have to be checked on by health workers.	Three (3) times a month 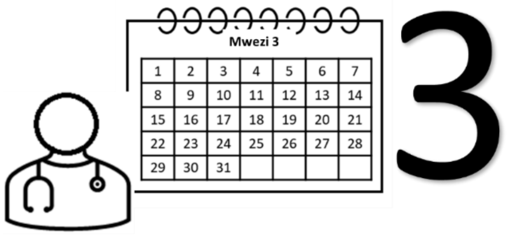	Three (3) times a month 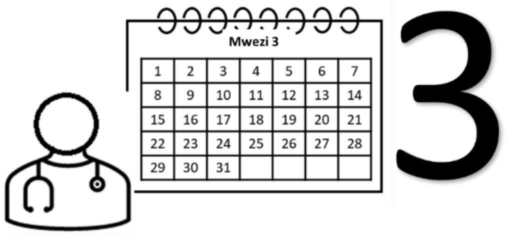	None 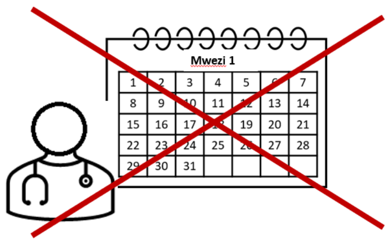
How much you would spend travelling from your home to a healthcare facility to access care in a year.	K2,400 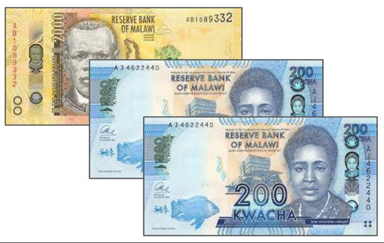	K6,000 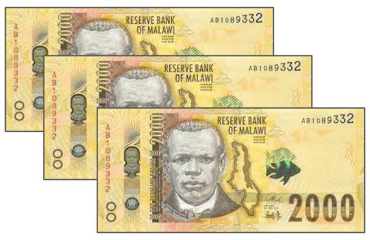	K0 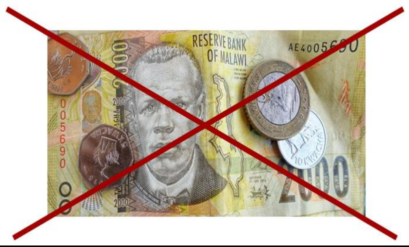
Please choose which treatment you prefer			
