# Supplementary material for: Treatment preferences among people at risk of developing tuberculosis: A discrete choice experiment
Source: PLOS Glob Public Health. 2024 Jul 19;4(7):e0002804. doi: 10.1371/journal.pgph.0002804 (PMC11259259; doi:10.1371/journal.pgph.0002804)
Supplement: S2 Appendix — (PDF) [file pgph.0002804.s007.pdf]

RADIO+ TB: DISCRETE CHOICE EXPERIMENT QUESTIONNAIRE

Scenario 1: **Ngati munayezetsa ndikupezeka ndi matenda, zimene zikutanthauza kuti chiopsezo chanu chodzakhala ndi TB mu miyezi 12 ikubwerayi chili pa 30%, ndi thandizo la mankhwala liti limene mungakonde mutalandira?**

	Thandizo A	Thandizo B	Palibe
Kutalika kwa nthawi yomwe mungakhale mukumwa mankhwala ngati muli pachiopsezo choti mukhoza kukhala ndi matenda a TB	miyezi itatu (3) mukumwa mapilisi 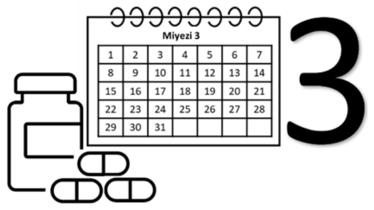	Miyezi inayi (4) mukumwa mapilisi 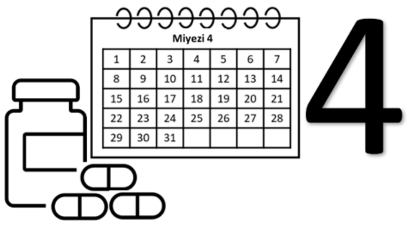	Palibe thandizo la mankhwala limene mungakonde 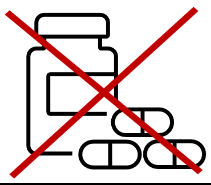
Nambala ya ma pilisi pa nthawi iliyonse imene mukumwa mankhwala	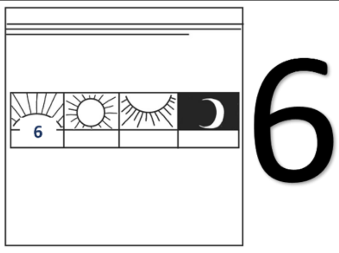	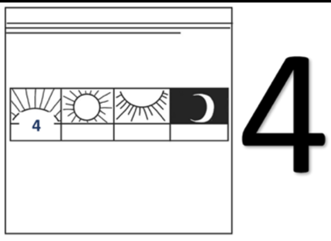	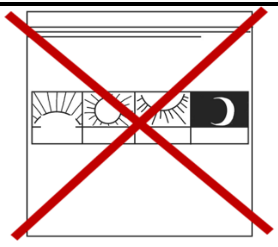
Kachepedwe ka chiopsezo choti mukhoza kudwala TB mukamaliza kulandira thandizo la mankhwala	50%<br>Chiopsezo cha matenda a TB mukamaliza kumwa mankhwala chitsika kuchoka pa 30% kufika pa 15% 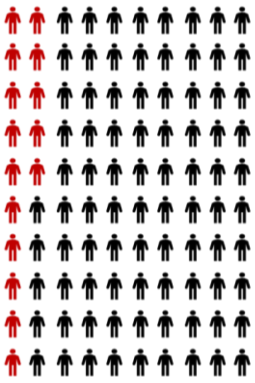	65%<br>Chiopsezo cha matenda a TB mukamaliza kumwa mankhwala chitsika kuchoka pa 30% kufika pa 11% 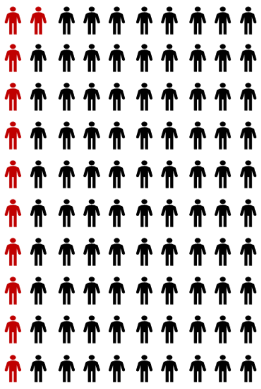	0%<br>Chiopsezo chikhalabe pa 30% 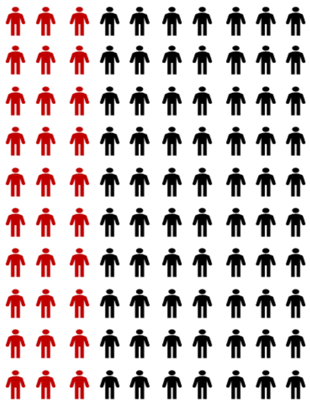
Kuona ngati mungapatsirebe ena matendawa ngakhale mutamaliza kumwa mankhwala	Aletseratu inu kupatsira ena TB. 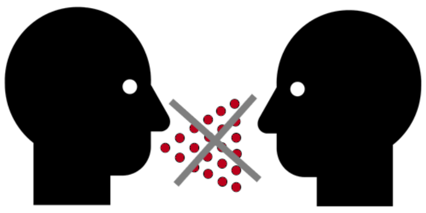	Aletseratu inu kupatsira ena TB. 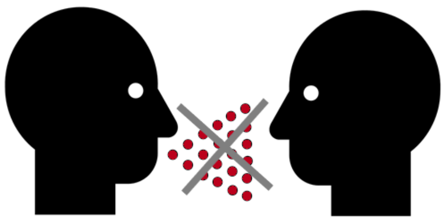	Kuthekera kopatsira ena TB sikuchepetsedwa. 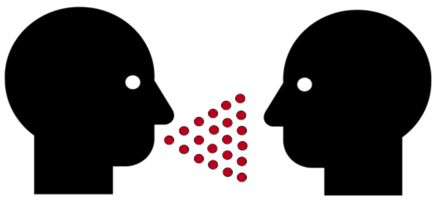
Zotsatira zosakhala bwino zimene zingabwere chifukwa cholandira mankhwala	Zotsatira zocheperako mphamvu tsiku lina lililonse – zokupangitsani kusasangalala pamene muli pamodzi ndi ena koma mukutha kugwira ntchito bwinobwino 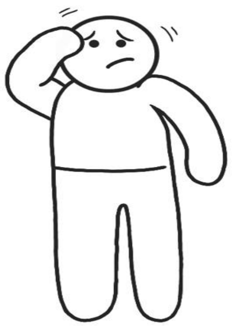	Zotsatira zosadetsa nkhawa ndipo zosazindikirika kwenikweni monga kumva ngati mukudwala kwa kanthawi kochepa 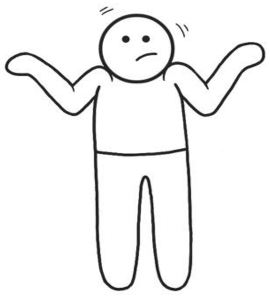	Palibe zotsatira zobwera chifukwa chokumwa mankhwala 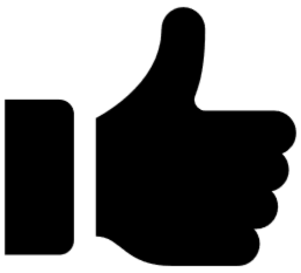
Mudzafunika kumaonedwa ndi azaumoyo mowirikiza bwanji	Palibe 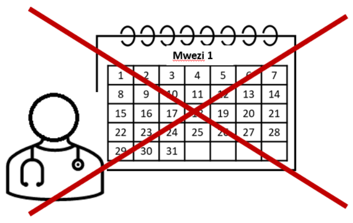	katatu (3) pa mwezi 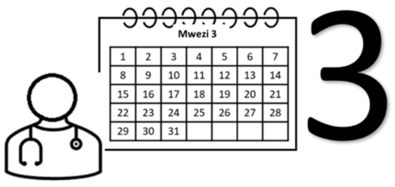	Palibe 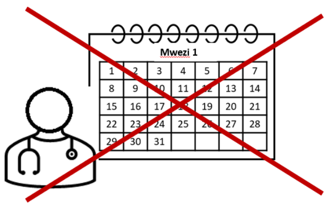
Ndalama zomwe mungagwiritse ntchito kuyenda kuchokera kunyumba kwanu kupita ku chipatala kukalandira thandizo la mankhwala pa chaka	K2,400 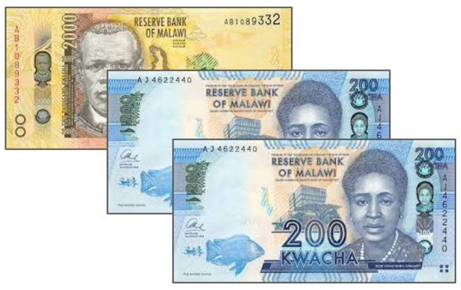	K6,000 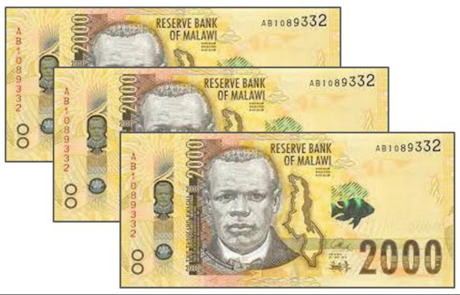	K0 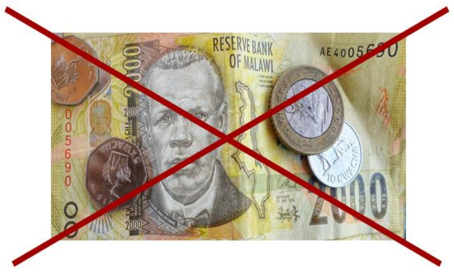
**Chonde sankhani mtundu wa thandizo la mankhwala limene mulingakonde**			

Scenario 2: Ngati munayezetsa ndikupezeka ndi matenda, zimene zikutanthauza kuti chiopsezo chanu chodzakhala ndi TB mu miyezi 12 ikubwerayi chili pa 10%, ndi thandizo liti la mankhwala limene mungakonde kulandira?

	Thandizo A	Thandizo B	Palibe
Kutalika kwa nthawi yomwe mungakhale mukumwa mankhwala ngati muli pachiopsezo choti mukhoza kukhala ndi matenda a TB	Miyezi iwiri (2) mukumwa mapilisi	miyezi itatu (3) mukumwa mapilisi	Palibe thandizo la mankhwala
Nambala ya ma pilisi pa nthawi iliyonse imene mukumwa mankhwala	4	2	0
Kachepedwe ka chiopsezo choti mukhoza kudwala TB mukamaliza kulandira thandizo la mankhwala	80%<br>Chiopsezo cha matenda a TB mukamaliza kumwa mankhwala chitsika kuchoka pa 10% kufika pa 2%	95%<br>Chiopsezo cha matenda a TB mukamaliza kumwa mankhwala chitsika kuchoka pa 10% kufik pa 1%	0%<br>Chiopsezo chikhalabe pa 10%
Kuona ngati mungapatsirebe ena matendawa ngakhale mutamaliza kumwa mankhwala onse	Achepetsa kuthekera kopatsira ena TB ndi theka	Kuthekera kopatsira ena TB sikuchepetsedwa	Kuthekera kopatsira ena TB sikuchepetsedwa
Zotsatira zosakhala bwino zimene zingabwere chifukwa cholandira mankhwala	Palibe zotsatira zosakhala bwino zobwera chifukwa chokumwa mankhwala.	Zotsatira zocheperako mphamvu tsiku lina lililonse – zokupangitsani kusasangalala pamene muli pamodzi ndi ena koma mukutha kugwira ntchito bwinobwino	Palibe zotsatira zosakhala bwino zobwera chifukwa chokumwa mankhwala.
Mudzafuna kumaonedwa ndi azaumoyo mowirikiza bwanji	Palibe	Palibe	Palibe
Ndalama zomwe mungagwiritse ntchito kuyenda kuchokera kunyumba kwanu kupita ku chipatala kukalandira thandizo la mankhwala pa chaka	K0	K6,000	K0

Chonde sankhani mtundu wa thandizo la mankhwala limene mulingakonde

Scenario 3: Ngati munayezetsa ndikupezeka ndi matenda zimene zikutanthauza kuti chiopsezo chanu chodzakhala ndi TB mu miyezi 12 ikubwerayi chili pa 50%, ndi thandizo liti la mankhwala limene mungakonde kulandira?

	Thandizo A	Thandizo B	Palibe
Kutalika kwa nthawi yomwe mungakhale mukumwa mankhwala ngati muli pachiopsezo choti mukhoza kukhala ndi matenda a TB	Miyezi inayi (4) mukumwa mapilisi 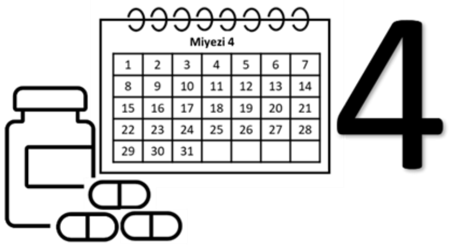	Miyezi isanu ndi umodzi (6) mukumwa mapilisi 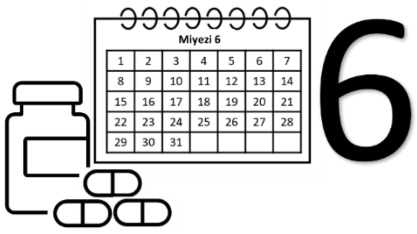	Palibe thandizo la mankhwala 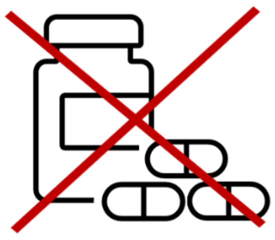
Nambala ya ma pilisi pa nthawi iliyonse imene mukumwa mankhwala	2 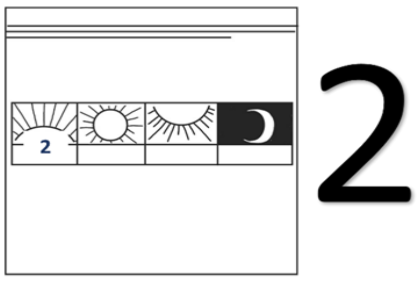	2 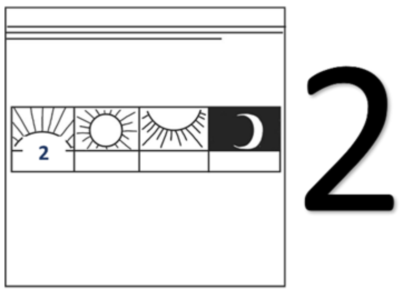	0 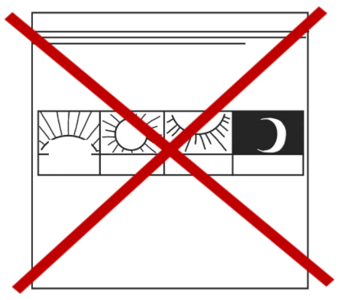
Kachepedwe ka chiopsezo choti mukhoza kudwala TB mukamaliza kulandira thandizo la mankhwala	50% Chiopsezo cha matenda a TB mukamaliza kumwa mankhwala chitsika kuchoka pa 50% kufika pa 25% 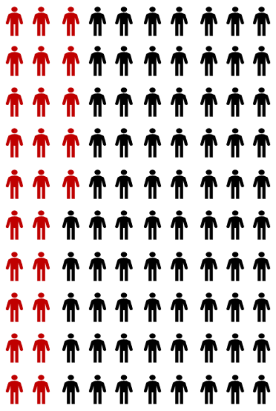	80% Chiopsezo cha matenda a TB mukamaliza kumwa mankhwala chitsika kuchoka pa 50% kufika pa 10% 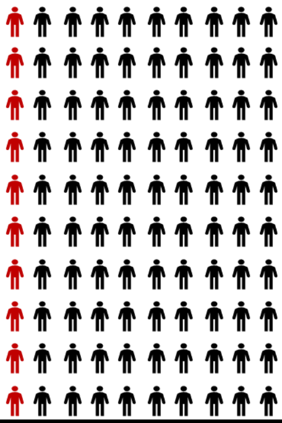	0% Chiopsezo chikhalabe pa 50% 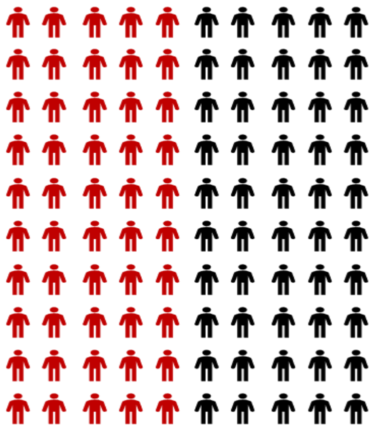
Kuona ngati mungapatsirebe ena matendawa ngakhale mutamaliza kumwa mankhwala onse	Kuthekera kopatsira ena TB sikuchepetsedwa 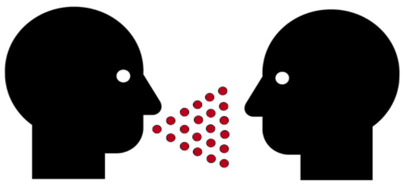	Kuthekera kopatsira ena TB sikuchepetsedwa 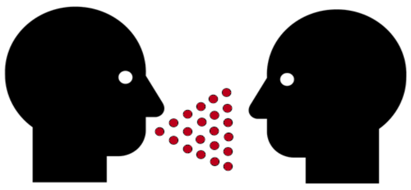	Kuthekera kopatsira ena TB sikuchepetsedwa 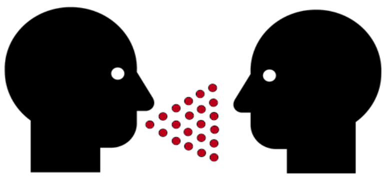
Zotsatira zosakhala bwino zimene zingabwere chifukwa cholandira mankhwala	Zotsatira zodetsa nkhawa pang'ono zomwe zingakupangitseni kuti musiye ntchito zanu nkupita kukaonana ndi adokotala 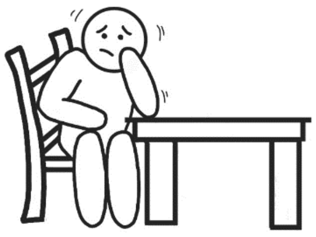	Palibe zotsatira zosakhala bwino zobwera chifukwa chokumwa mankhwala. 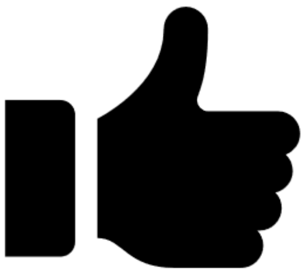	Palibe zotsatira zosakhala bwino zobwera chifukwa chokumwa mankhwala. 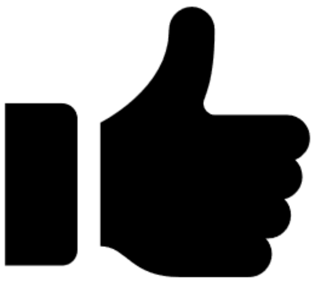
Mudzafuna kumaonedwa ndi azaumoyo mowirikiza bwanji	Palibe 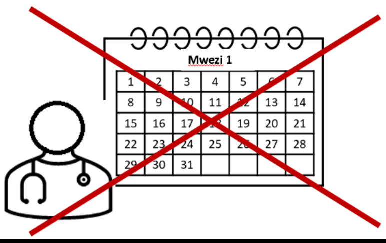	Kamodzi (1) pa mwezi 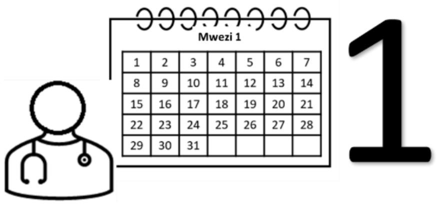	Palibe 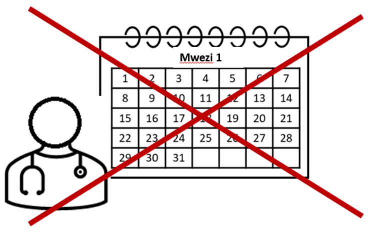
Ndalama zomwe mungagwiritse ntchito kuyenda kuchokera kunyumba kwanu kupita ku chipatala kukalandira thandizo la mankhwala pa chaka	K6000 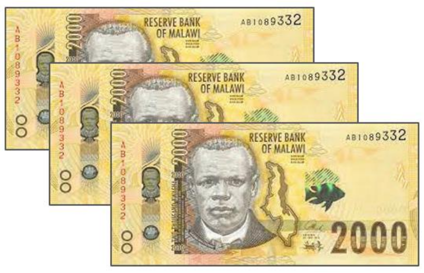	K0 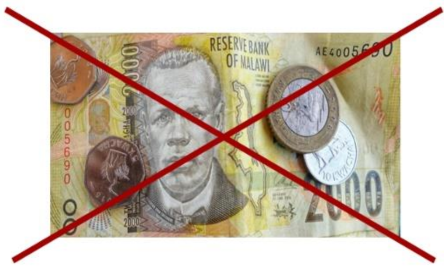	K0 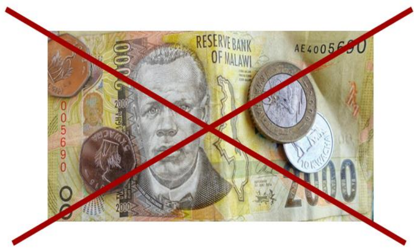
Chonde sankhani mtundu wa thandizo la mankhwala limene mulingakonde			

Scenario 4: **Ngati munayezetsa ndikupezeka ndi matenda zimene zikutanthauza kuti chiopsezo chanu chodzakhala ndi TB mu miyezi 12 ikubwerayi chili pa 10%, ndi thandizo liti la mankhwala limene mungakonde kulandira?**

	Thandizo A	Thandizo B	Palibe
Kutalika kwa nthawi yomwe mungakhale mukumwa mankhwala ngati muli pachiopsezo choti mukhoza kukhala ndi matenda a TB	Miyezi isanu ndi umodzi (6) mukumwa mapilisi 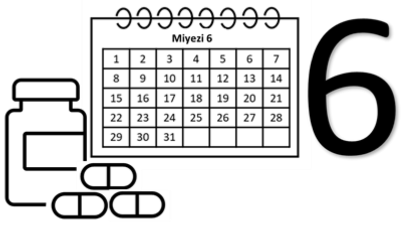	Miyezi inayi (4) mukumwa mapilisi 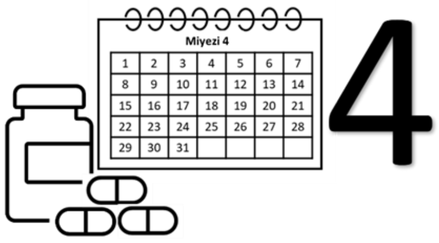	Palibe thandizo la mankhwala 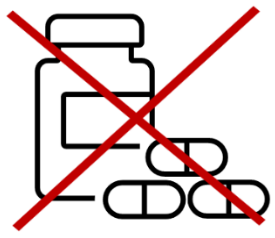
Nambala ya ma pilisi pa nthawi iliyonse imene mukumwa mankhwala	4 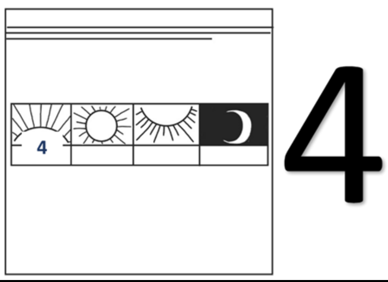	6 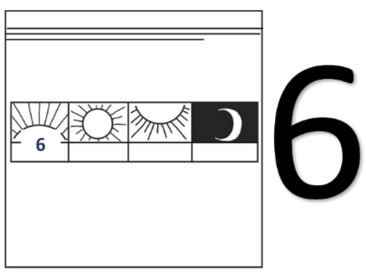	0 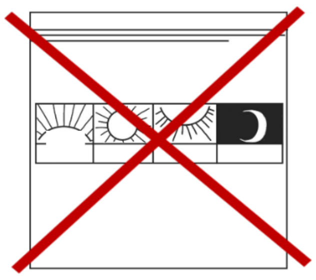
Kachepedwe ka chiopsezo choti mukhoza kudwala chifukwa cha matenda a TB, mukamaliza kulandira thandizo la mankhwala.	95% Chiopsezo cha matenda a TB mukamaliza kumwa mankhwala chitsika kuchoka pa 10% kufika pa 1% 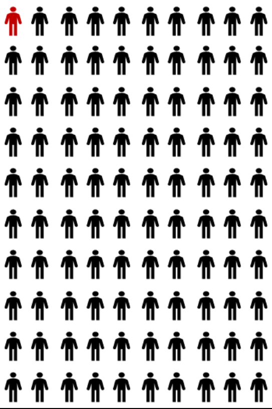	65% Chiopsezo cha matenda a TB mukamaliza kumwa mankhwala chitsika kuchoka pa 10% kufika pa 4% 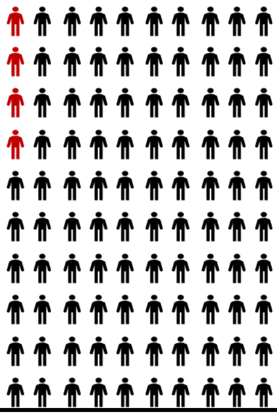	0% Chiopsezo chikhalabe pa 10% 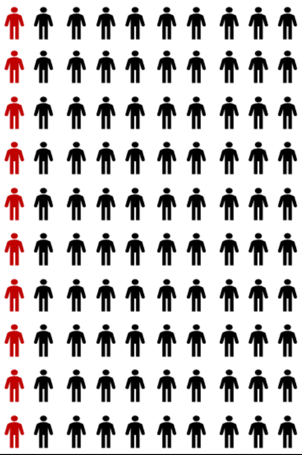
Kuona ngati mungapatsirebe ena matendawa ngakhale mutamaliza kumwa mankhwala onse	Aletseratu inu kupatsira ena TB. 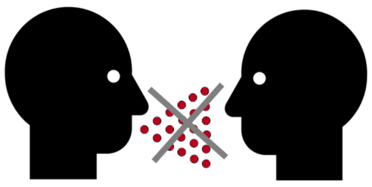	Kuthekera kopatsira ena TB sikuchepetsedwa 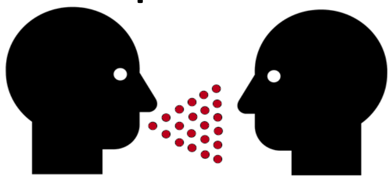	Kuthekera kopatsira ena TB sikuchepetsedwa 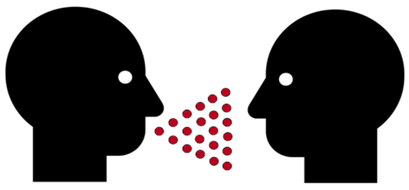
Zotsatira zosakhala bwino zimene zingabwere chifukwa cholandira mankhwala	Zotsatira zodetsa nkhawa pang'ono zomwe zingakupangitseni kuti musiye ntchito zanu nkupita kukaonana ndi adokotala 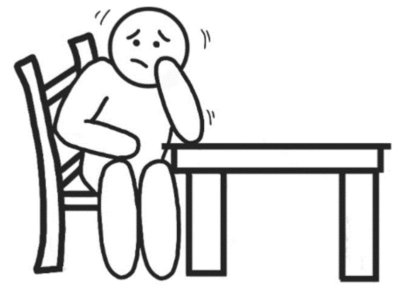	Zotsatira zodetsa nkhawa pang'ono zomwe zingakupangitseni kuti musiye ntchito zanu nkupita kukaonana ndi adokotala 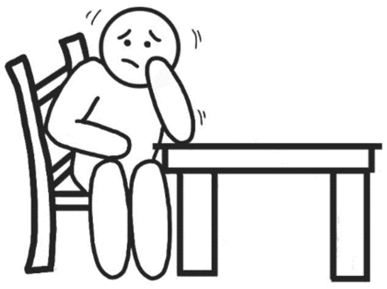	Palibe zotsatira zosakhala bwino zobwera chifukwa chokumwa mankhwala. 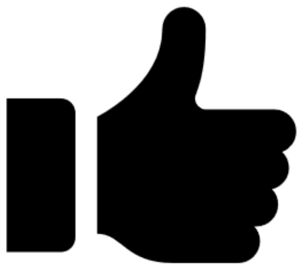
Mudzafuna kumaonedwa ndi azaumoyo mowirikiza bwanji	Kamodzi (1) pa mwezi 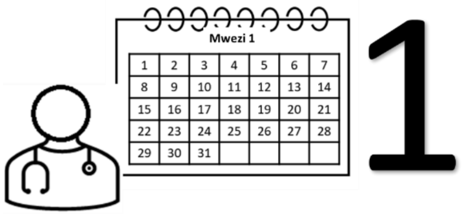	Palibe 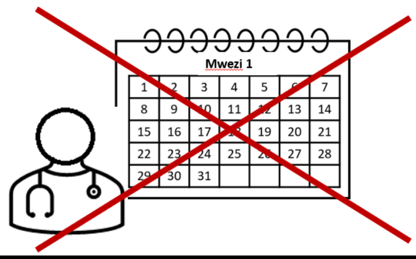	Palibe 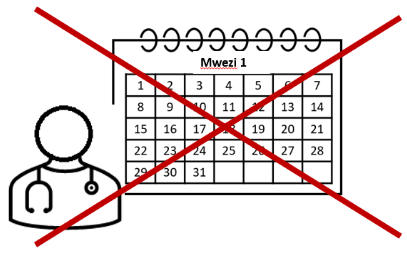
Ndalama zomwe mungagwiritse ntchito kuyenda kuchokera kunyumba kwanu kupita ku chipatala kukalandira thandizo la mankhwala pa chaka	K6,000 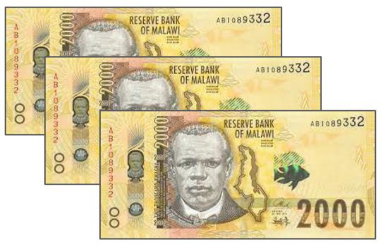	K2,400 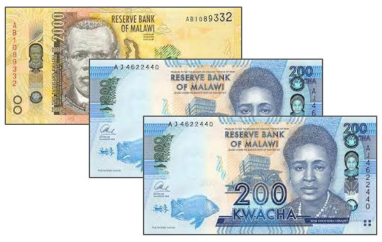	K0 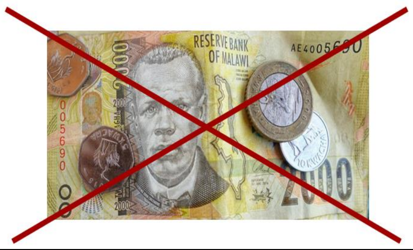

Chonde sankhani mtundu wa thandizo la mankhwala limene mulingakonde

Scenario 5: **Ngati munayezetsa ndikupezeka ndi matenda zimene zikutanthauza kuti chiopsezo chanu chodzakhala ndi TB mu miyezi 12 ikubwerayi chili pa 10%, ndi thandizo liti la mankhwala limene mungakonde kulandira?**

	Thandizo A	Thandizo B	Palibe
Kutalika kwa nthawi yomwe mungakhale mukumwa mankhwala ngati muli pachiopsezo choti mukhoza kukhala ndi matenda a TB	Miyezi isanu (5) mukumwa mapilisi 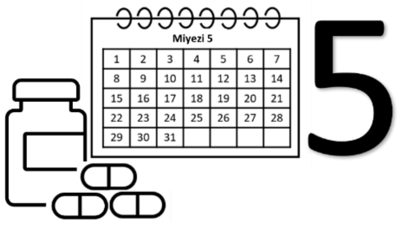	Miyezi isanu (5) mukumwa mapilisi 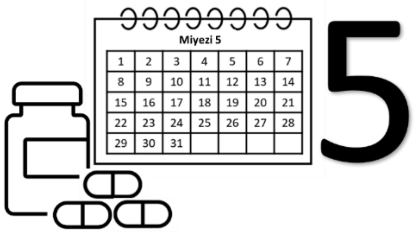	Palibe thandizo la mankhwala 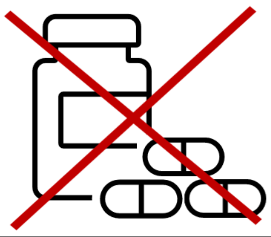
Nambala ya ma pilisi pa nthawi iliyonse imene mukumwa mankhwala	6 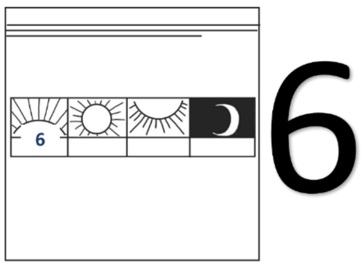	6 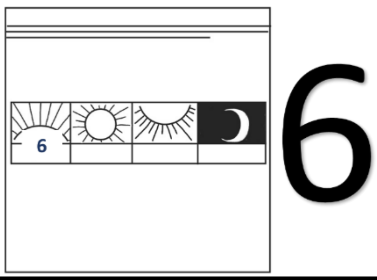	0 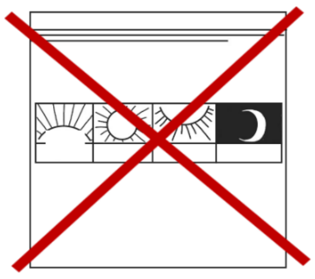
Kuchepedwe ka chiopsezo choti mukhoza kudwala chifukwa cha matenda a TB, mukamaliza kulandira thandizo la mankhwala.	95% Chiopsezo cha matenda a TB mukamaliza kumwa mankhwala chitsika kuchoka pa 10% kufika pa 1% 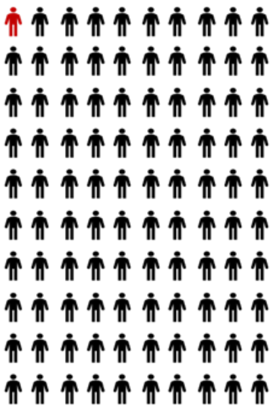	50% Chiopsezo cha matenda a TB mukamaliza kumwa mankhwala chitsika kuchoka pa 10% kufika pa 5% 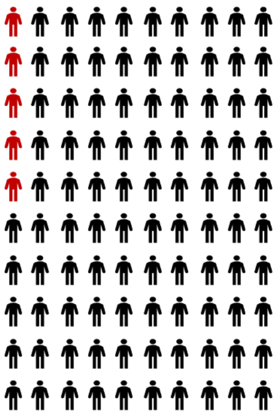	0% Chiopsezo chikhalabe pa 10% 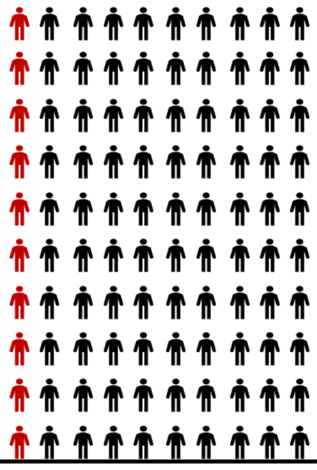
Kuona ngati mungapatsirebe ena matendawa ngakhale mutamaliza kumwa mankhwala	Kuthekera kopatsira ena TB sikuchepetsedwa 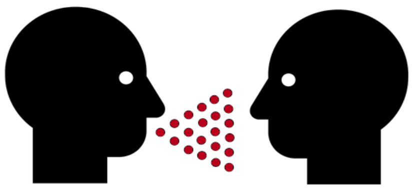	Aletseratu inu kupatsira ena TB. 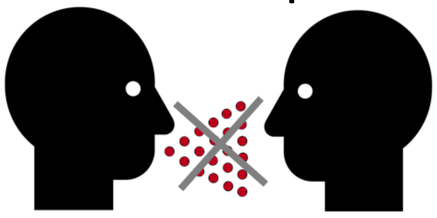	Kuthekera kopatsira ena TB sikuchepetsedwa 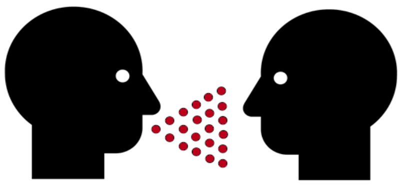
Zotsatira zosakhala bwino zimene zingabwere chifukwa cholandira mankhwala	Zotsatira zosadetsa nkhawa ndipo zosazindikirika kwenikweni monga kumva ngati mukudwala kwa kanthawi kochepa 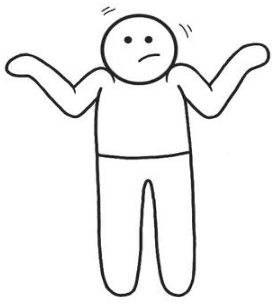	Zotsatira zocheperako mphamvu tsiku lina lililonse – zokupangitsani kusasangalala pamene muli pamodzi ndi ena koma mukutha kugwira ntchito bwinobwino 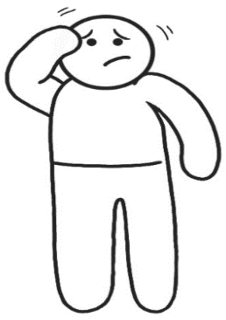	Palibe zotsatira zosakhala bwino zobwera chifukwa chokumwa mankhwala. 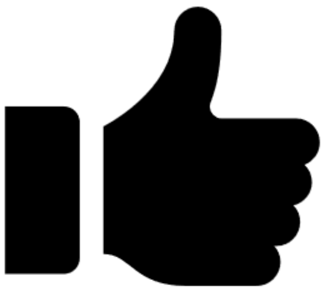
Mudzafuna kumaonedwa ndi azaumoyo mowirikiza bwanji	katatu (3) pa mwezi 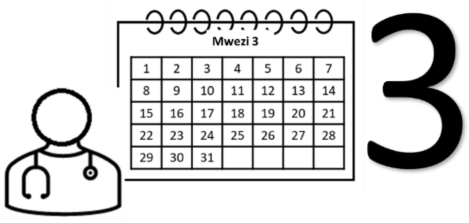	katatu (3) pa mwezi 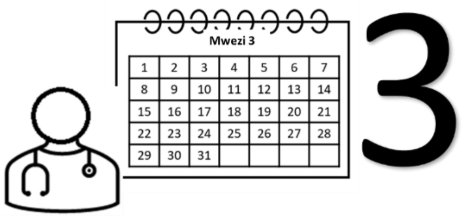	Palibe 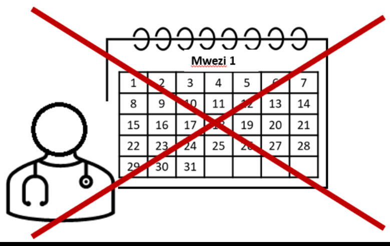
Ndalama zomwe mungagwiritse ntchito kuyenda kuchokera kunyumba kwanu kupita ku chipatala kukalandira thandizo la mankhwala pa chaka	K2,400 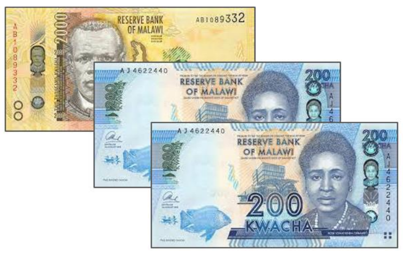	K6,000 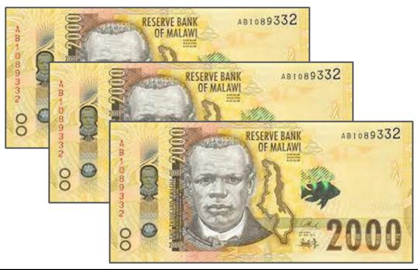	K0 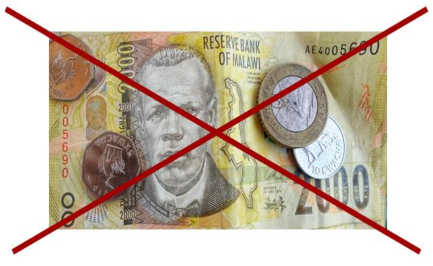

Chonde sankhani mtundu wa thandizo la mankhwala limene mulingakonde

Scenario 6: Ngati munayezetsa ndikupezeka ndi matenda zimene zikutanthauza kuti chiopsezo chanu chodzakhala ndi TB mu miyezi 12 ikubwerayi chili pa 30%, ndi thandizo liti la mankhwala limene mungakonde kulandira?

	Thandizo A	Thandizo B	Palibe
Kutalika kwa nthawi yomwe mungakhale mukumwa mankhwala ngati muli pachiopsezo choti mukhoza kukhala ndi matenda a TB	Miyezi itatu (3) mukumwa mapilisi 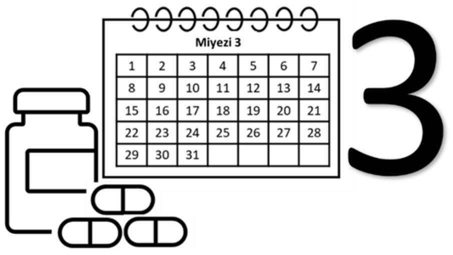	Miyezi itatu (3) mukumwa mapilisi 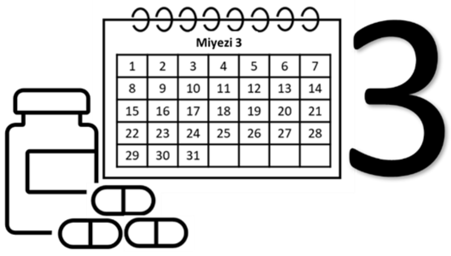	Palibe thandizo la mankhwala 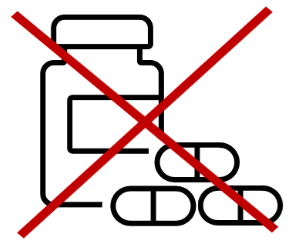
Nambala ya ma pilisi pa nthawi iliyonse imene mukumwa mankhwala	2 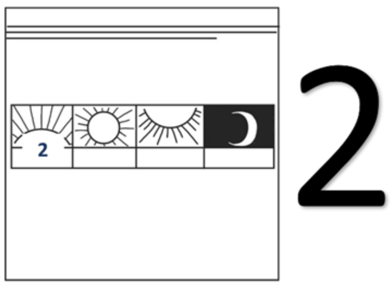	6 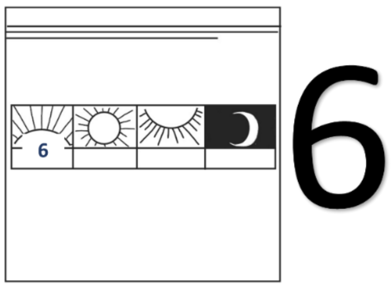	0 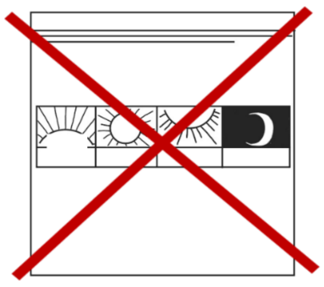
Kuchepedwe ka chiopsezo choti mukhoza kudwala chifukwa cha matenda a TB, mukamaliza kulandira thandizo la mankhwala.	80% Chiopsezo cha matenda a TB mukamaliza kumwa mankhwala chitsika kuchoka pa 30% kufika pa 6% 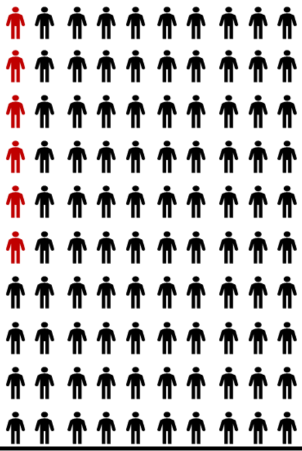	80% Chiopsezo cha matenda a TB mukamaliza kumwa mankhwala chitsika kuchoka pa 30% kufika pa 6% 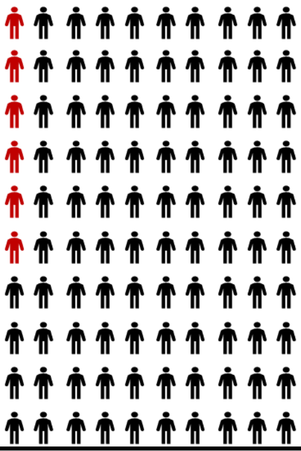	0% Chiopsezo chikhalabe pa 30% 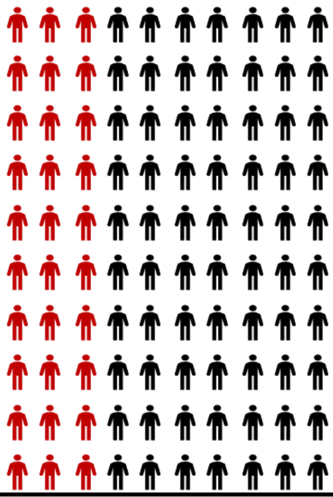
Kuona ngati mungapatsirebe ena matendawa ngakhale mutamaliza kumwa mankhwala	Kuthekera kopatsira ena TB sikuchepetsedwa 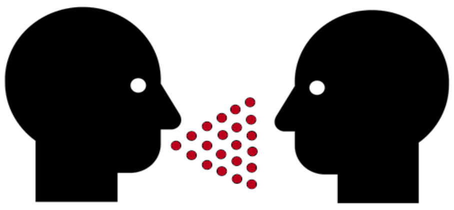	Achepetsa kuthekera koti mukhoza kupatsira ena TB ndi theka. 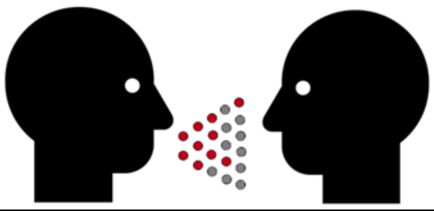	Kuthekera kopatsira ena TB sikuchepetsedwa 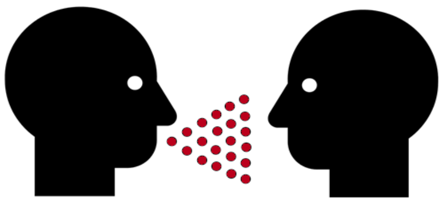
Zotsatira zosakhala bwino zimene zingabwere chifukwa cholandira mankhwala	Zotsatira zosadetsa nkhawa ndipo zosazindikirika kwenikweni monga kumva ngati mukudwala kwa kanthawi kochepa 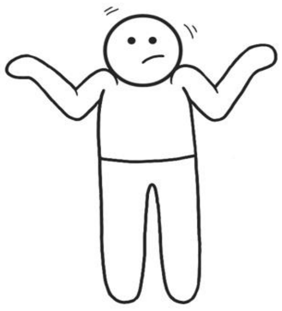	Palibe zotsatira zosakhala bwino zobwera chifukwa chokumwa mankhwala. 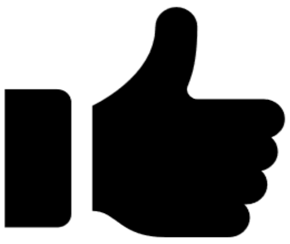	Palibe zotsatira zosakhala bwino zobwera chifukwa chokumwa mankhwala. 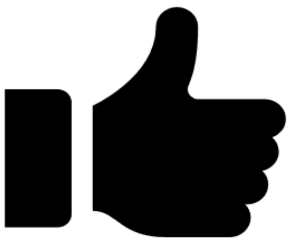
Mudzafuna kumaonedwa ndi azaumoyo mowirikiza bwanji	Katatu (3) opa mwezi 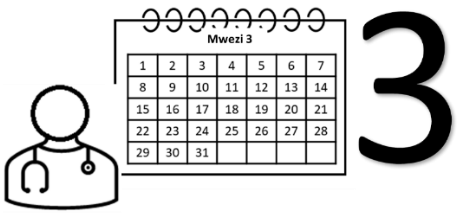	kamodzi (1) pa mwezi 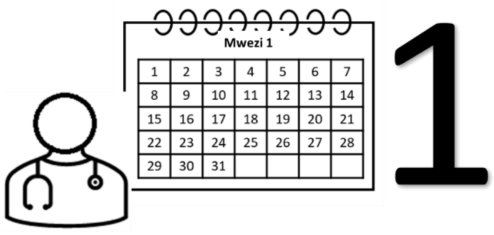	Palibe 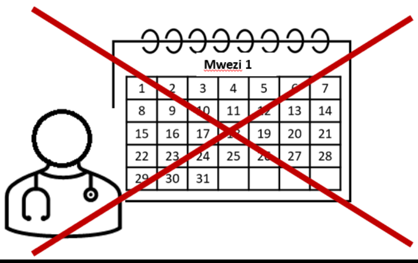
Ndalama zomwe mungagwiritse ntchito kuyenda kuchokera kunyumba kwanu kupita ku chipatala kukalandira thandizo la mankhwala pa chaka	K0 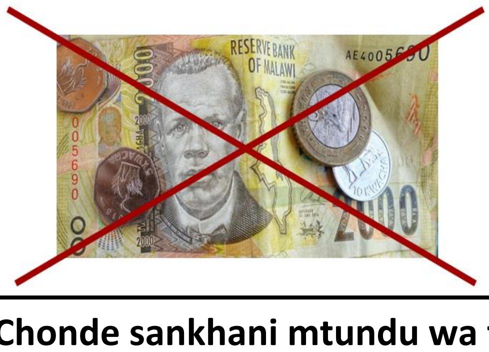	K2,400 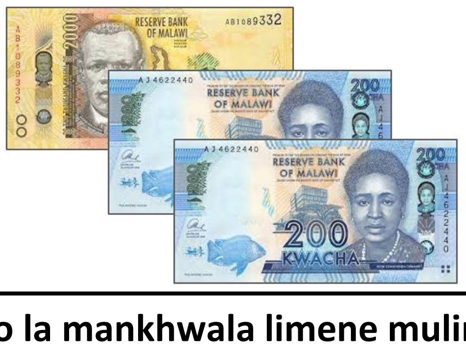	K0 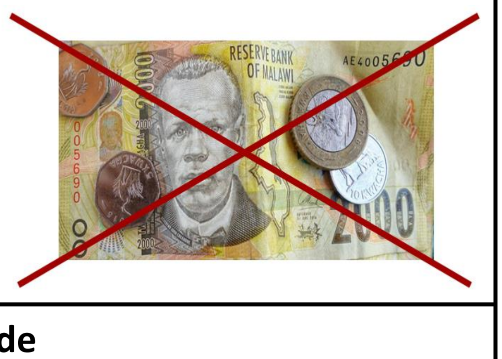

Chonde sankhani mtundu wa thandizo la mankhwala limene mulingakonde

Scenario 7: **Ngati munayezetsa ndikupezeka ndi matenda zimene zikutanthauza kuti chiopsezo chanu chodzakhala ndi TB mu miyezi 12 ikubwerayi chili pa 50%, ndi thandizo liti la mankhwala limene mungakonde kulandira?**

	Thandizo A	Thandizo B	Palibe
Kutalika kwa nthawi yomwe mungakhale mukumwa mankhwala ngati muli pachiopsezo choti mukhoza kukhala ndi matenda a TB	Miyezi inayi (4) mukumwa mapilisi 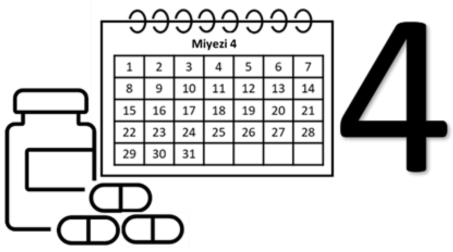	Miyezi iwiri (2) mukumwa mapilisi 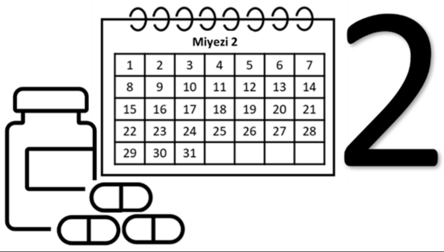	Palibe thandizo la mankhwala 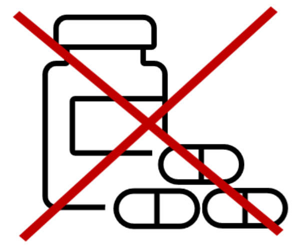
Nambala ya ma pilisi pa nthawi iliyonse imene mukumwa mankhwala	6 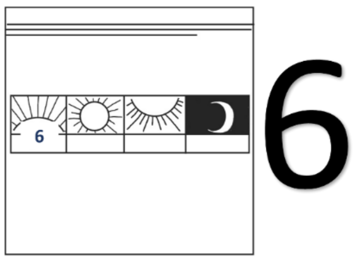	2 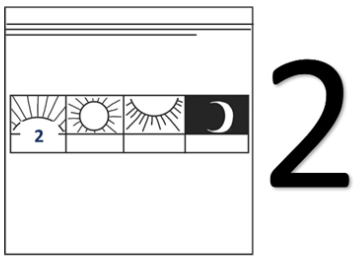	0 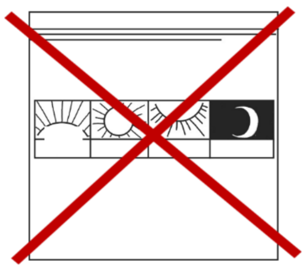
Kuchepedwe ka chiopsezo choti mukhoza kudwala chifukwa cha matenda a TB, mukamaliza kulandira thandizo la mankhwala.	65% Chiopsezo cha matenda a TB mukamaliza kumwa mankhwala chitsika kuchoka pa 50% kufika pa 18% 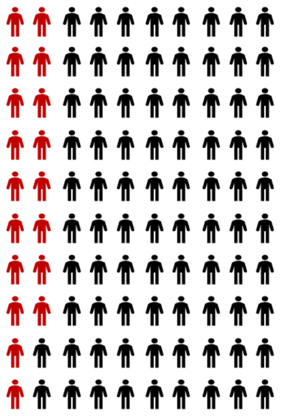	95% Chiopsezo cha matenda a TB mukamaliza kumwa mankhwala chitsika kuchoka pa 50% kufika pa 2% 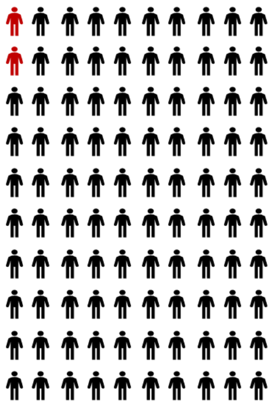	0% Chiopsezo chikhalabe pa 50% 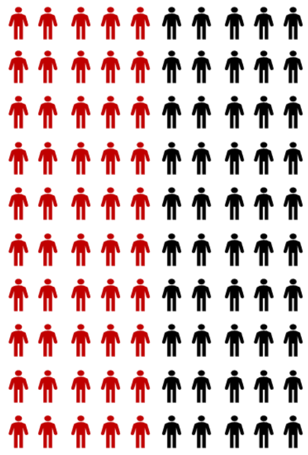
Kuona ngati mungapatsirebe ena matendawa ngakhale mutamaliza kumwa mankhwala	Aletseratu kuti mupatsire ena TB. 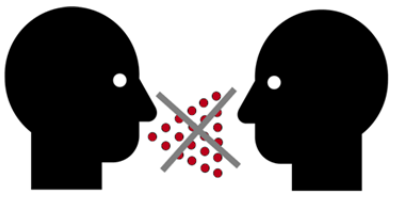	Aletseratu kuti mupatsire ena TB. 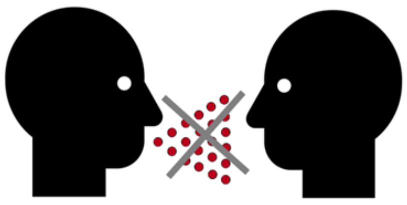	Kuthekera kopatsira ena TB sikuchepetsedwa 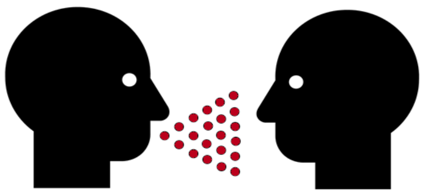
Zotsatira zosakhala bwino zimene zingabwere chifukwa cholandira mankhwala	Zotsatira zosadetsa nkhawa ndipo zosazindikirika kwenikweni monga kumva ngati mukudwala kwa kanthawi kochepa 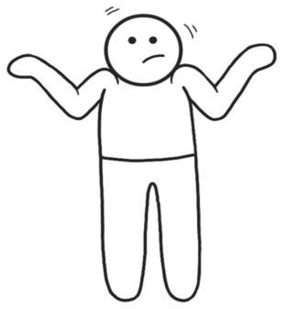	Zotsatira zodetsa nkhawa pang'ono zomwe zingakupangitseni kuti musiye ntchito zanu nkupita kukaonana ndi adokotala 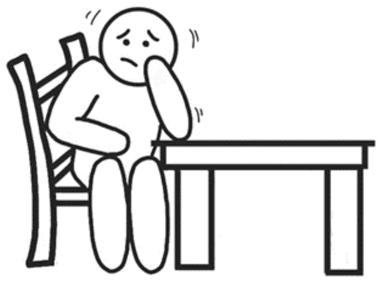	Palibe zotsatira zosakhala bwino zobwera chifukwa chokumwa mankhwala. 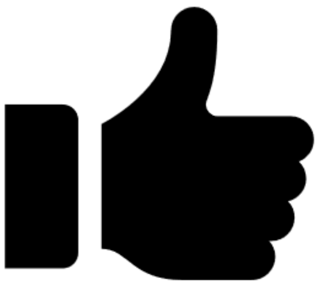
Mudzafuna kumaonedwa ndi azaumoyo mowirikiza bwanji	Palibe 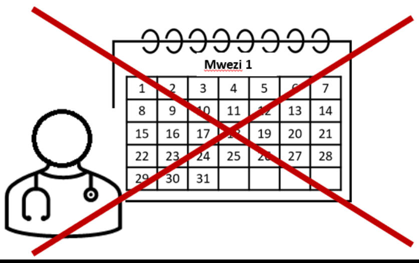	Katatu (3) opa mwezi 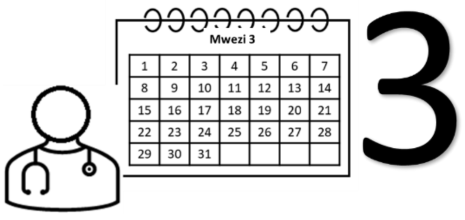	Palibe 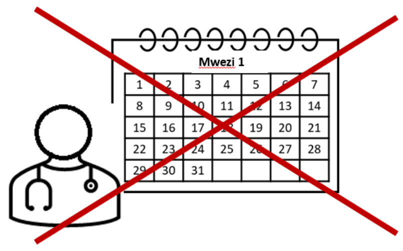
Ndalama zomwe mungagwiritse ntchito kuyenda kuchokera kunyumba kwanu kupita ku chipatala kukalandira thandizo la mankhwala pa chaka	K6,000 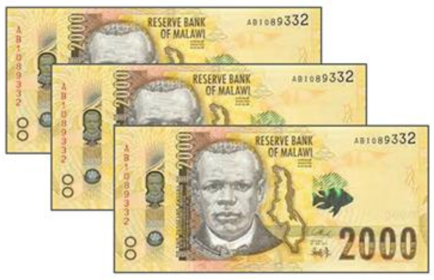	K2400 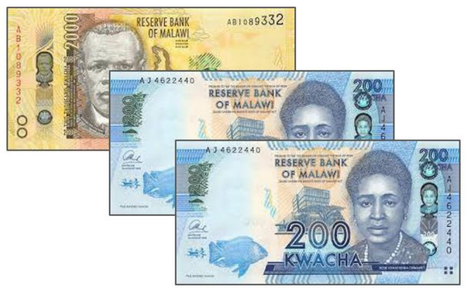	K0 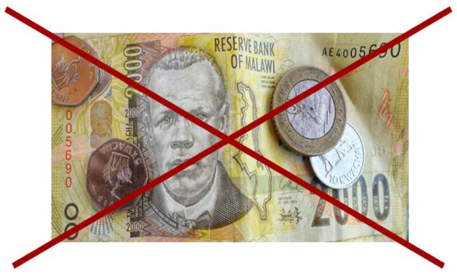

Chonde sankhani mtundu wa thandizo la mankhwala limene mulingakonde

Scenario 8: **Ngati munayezetsa ndikupezeka ndi matenda zimene zikutanthauza kuti chiopsezo chanu chodzakhala ndi TB mu miyezi 12 ikubwerayi chili pa 50%, ndi thandizo liti la mankhwala limene mungakonde kulandira?**

	Thandizo A	Thandizo B	Palibe
Kutalika kwa nthawi yomwe mungakhale mukumwa mankhwala ngati muli pachiopsezo choti mukhoza kukhala ndi matenda a TB	Miyezi isanu ndi umodzi (6) mukumwa mapilisi 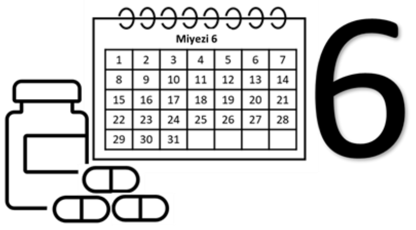	Miyezi isanu (5) mukumwa mapilisi 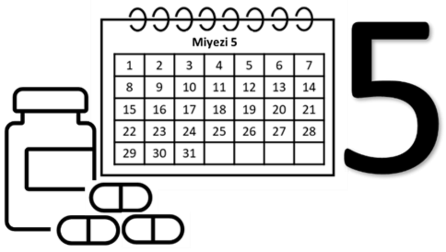	Palibe thandizo la mankhwala 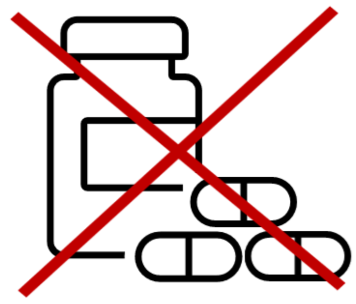
Nambala ya ma pilisi pa nthawi iliyonse imene mukumwa mankhwala	2 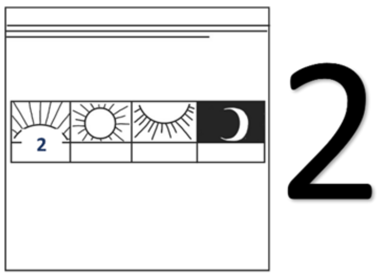	4 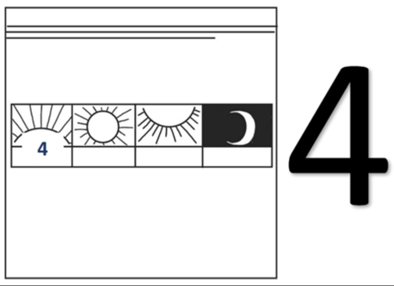	0 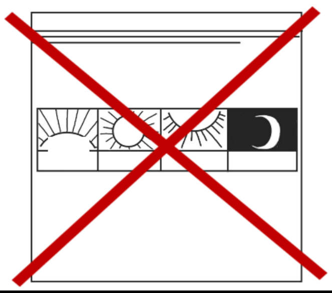
Kuchepedwe ka chiopsezo choti mukhoza kudwala chifukwa cha matenda a TB, mukamaliza kulandira thandizo la mankhwala.	50% Chiopsezo cha matenda a TB mukamaliza kumwa mankhwala chitsika kuchoka pa 50% kufika pa 25% 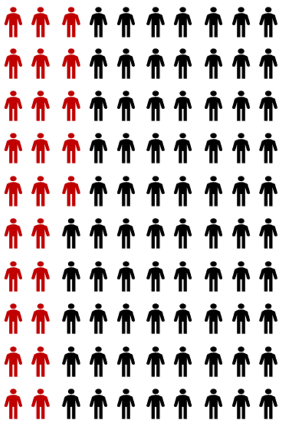	50% Chiopsezo cha matenda a TB mukamaliza kumwa mankhwala chitsika kuchoka pa 50% kufika pa 25% 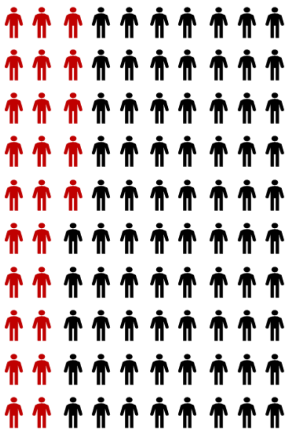	0% Chiopsezo chikhalabe pa 50% 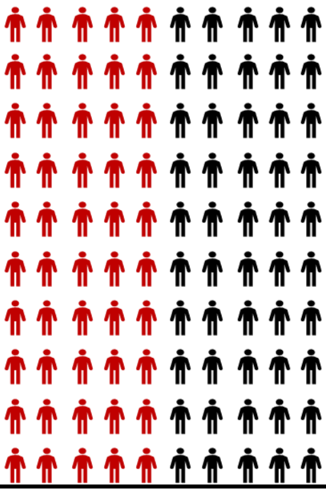
Kuona ngati mungapatsirebe ena matendawa ngakhale mutamaliza kumwa mankhwala	Achepetsa kuthekera koti mukhoza kupatsira ena TB ndi theka. 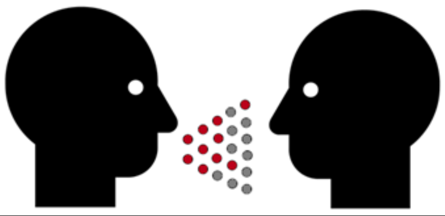	Kuthekera kopatsira ena TB sikuchepetsedwa 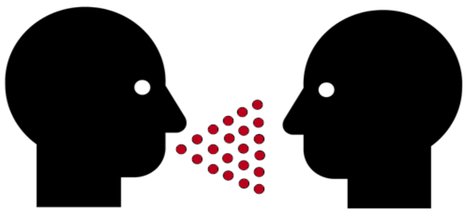	Kuthekera kopatsira ena TB sikuchepetsedwa 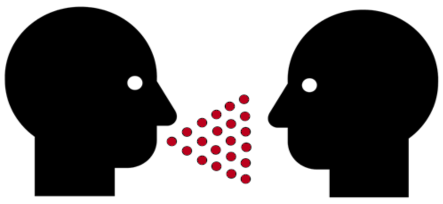
Zotsatira zosakhala bwino zimene zingabwere chifukwa cholandira mankhwala	Palibe zotsatira zosakhala bwino zobwera chifukwa chokumwa mankhwala. 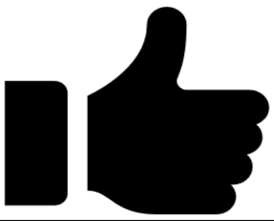	Palibe zotsatira zosakhala bwino zobwera chifukwa chokumwa mankhwala. 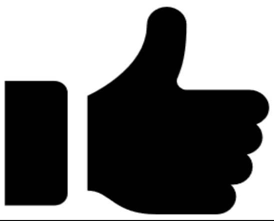	Palibe zotsatira zosakhala bwino zobwera chifukwa chokumwa mankhwala. 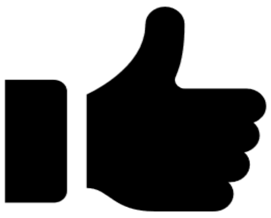
Mudzafuna kumaonedwa ndi azaumoyo mowirikiza bwanji	katatu (3) pa mwezi 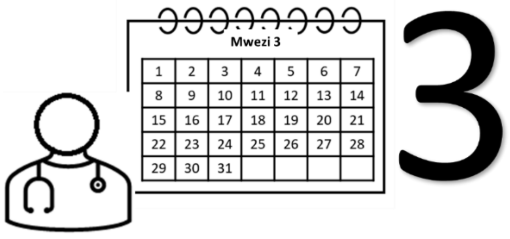	Palibe 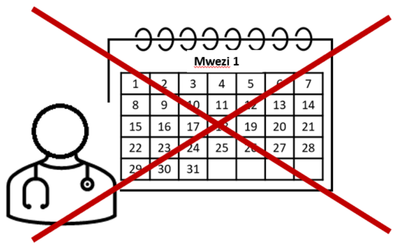	Palibe 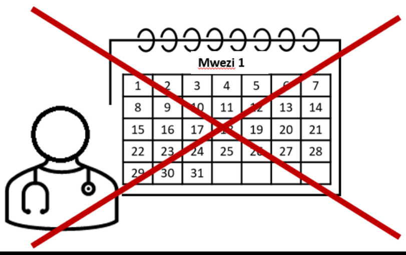
Ndalama zomwe mungagwiritse ntchito kuyenda kuchokera kunyumba kwanu kupita ku chipatala kukalandira thandizo la mankhwala pa chaka	K0 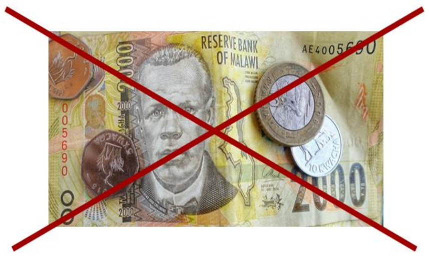	K0 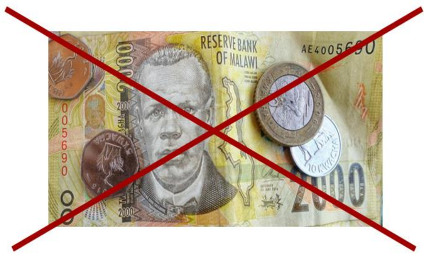	K0 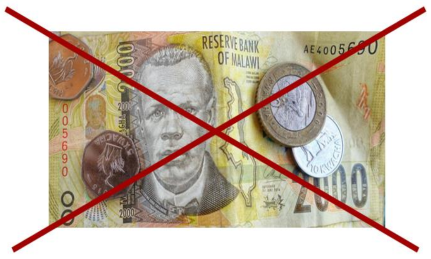

Chonde sankhani mtundu wa thandizo la mankhwala limene mulingakonde

Scenario 9: Ngati munayezetsa ndikupezeka ndi matenda zimene zikutanthauza kuti chiopsezo chanu chodzakhala ndi TB mu miyezi 12 ikubwerayi chili pa 30%, ndi thandizo liti la mankhwala limene mungakonde kulandira?

	Thandizo A	Thandizo B	Palibe
Kutalika kwa nthawi yomwe mungakhale mukumwa mankhwala ngati muli pachiopsezo choti mukhoza kukhala ndi matenda a TB	Miyezi isanu (5) mukumwa mapilisi 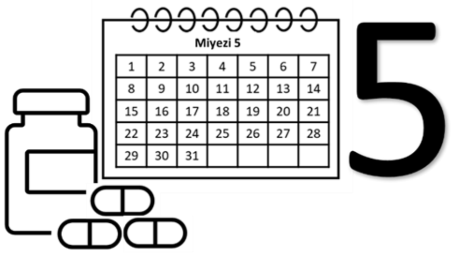	Miyezi iwiri (2) mukumwa mapilisi 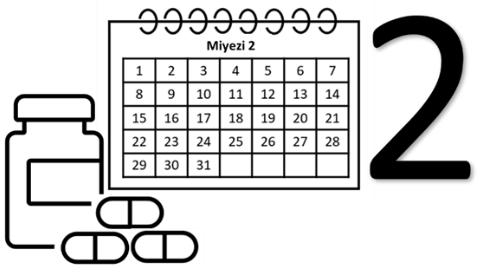	Palibe thandizo la mankhwala 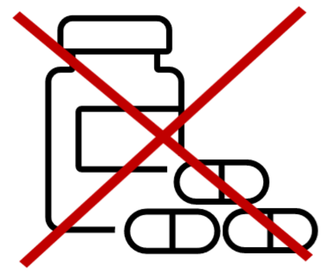
Nambala ya ma pilisi pa nthawi iliyonse imene mukumwa mankhwala	4 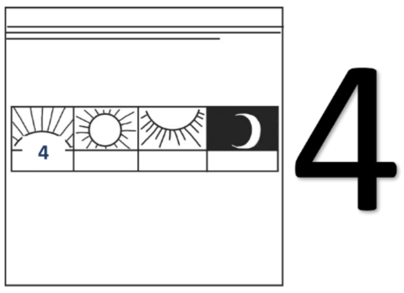	4 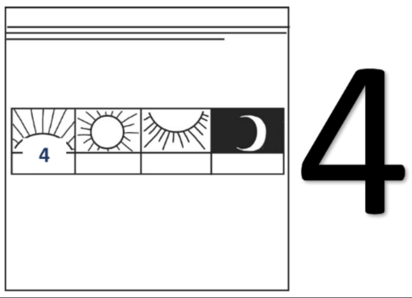	0 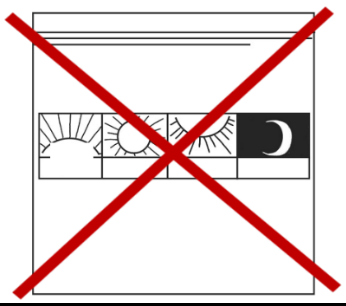
Kuchepedwe ka chiopsezo choti mukhoza kudwala chifukwa cha matenda a TB, mukamaliza kulandira thandizo la mankhwala.	65% Chiopsezo cha matenda a TB mukamaliza kumwa mankhwala chitsika kuchoka pa 30% kufika pa 11% 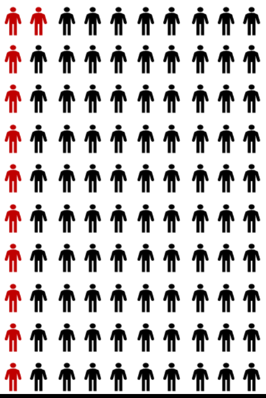	50% Chiopsezo cha matenda a TB mukamaliza kumwa mankhwala chitsika kuchoka pa 30% kufika pa 15% 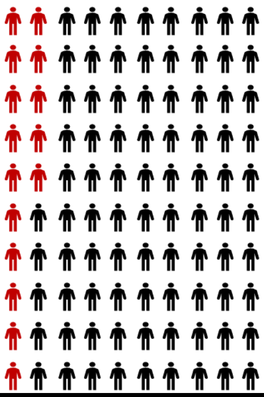	0% Chiopsezo chikhalabe pa 30% 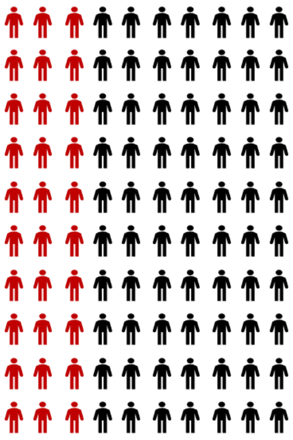
Kuona ngati mungapatsirebe ena matendawa ngakhale mutamaliza kumwa mankhwala	Achepetsa kuthekera koti mukhoza kupatsira ena TB ndi theka. 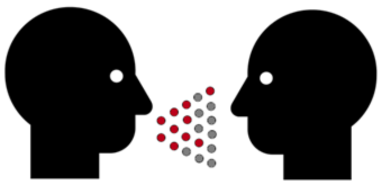	Achepetsa kuthekera koti mukhoza kupatsira ena TB ndi theka. 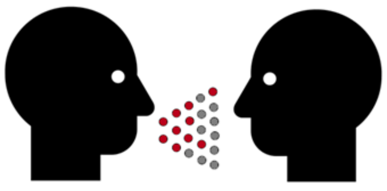	Kuthekera kopatsira ena TB sikuchepetsedwa 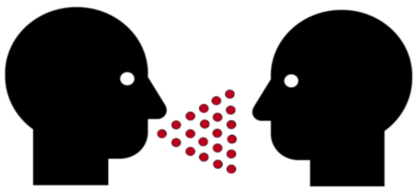
Zotsatira zosakhala bwino zimene zingabwere chifukwa cholandira mankhwala	Zotsatira zocheperako mphamvu tsiku lina lililonse – zokupangitsani kusasangalala pamene muli pamodzi ndi ena koma mukutha kugwira ntchito bwinobwino 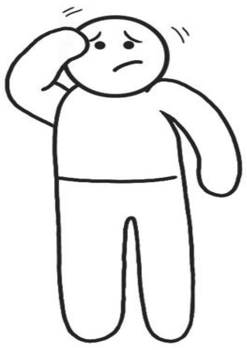	Zotsatira zosadetsa nkhawa ndipo zosazindikirika kwenikweni monga kumva ngati mukudwala kwa kanthawi kochepa 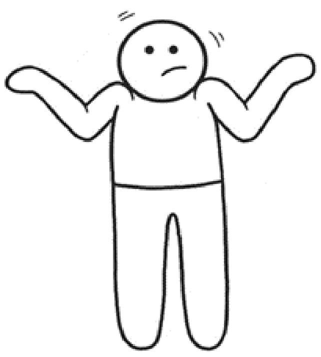	Palibe zotsatira zosakhala bwino zobwera chifukwa chokumwa mankhwala. 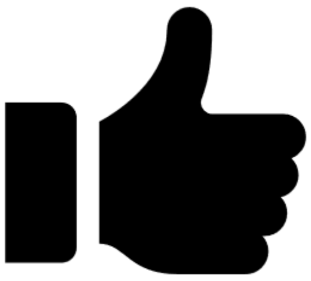
Mudzafuna kumaonedwa ndi azaumoyo mowirikiza bwanji	kamodzi (1) pa mwezi 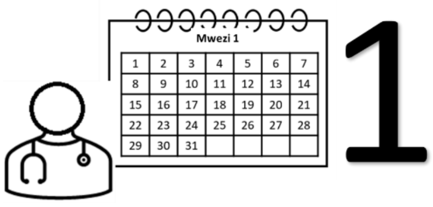	kamodzi (1) pa mwezi 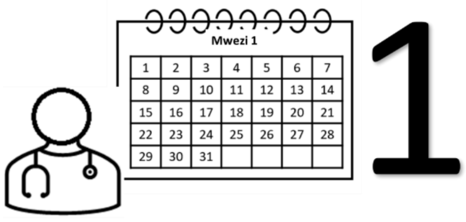	Palibe 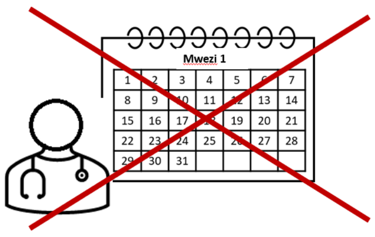
Ndalama zomwe mungagwiritse ntchito kuyenda kuchokera kunyumba kwanu kupita ku chipatala kukalandira thandizo la mankhwala pa chaka	K0 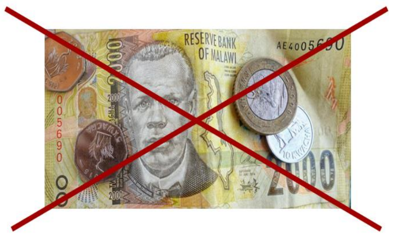	K0 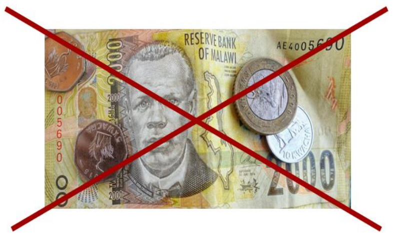	K0 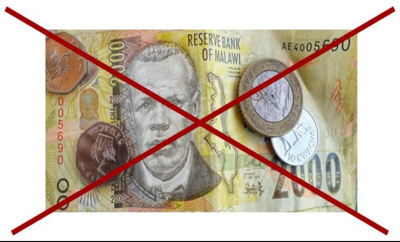

Chonde sankhani mtundu wa thandizo la mankhwala limene mulingakonde

Scenario 10: Ngati munayezetsa ndikupezeka ndi matenda zimene zikutanthauza kuti chiopsezo chanu chodzakhala ndi TB mu miyezi 12 ikubwerayi chili pa 10%, ndi thandizo liti la mankhwala limene mungakonde kulandira?

	Thandizo A	Thandizo B	Palibe
Kutalika kwa nthawi yomwe mungakhale mukumwa mankhwala ngati muli pachiopsezo choti mukhoza kukhala ndi matenda a TB	Miyezi iwiri (2) mukumwa mapilisi 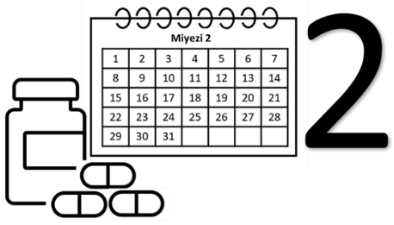	Miyezi isanu ndi umodzi (6) mukumwa mapilisi 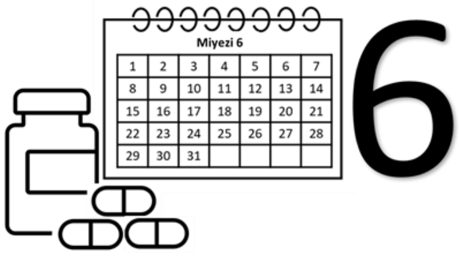	Palibe thandizo la mankhwala 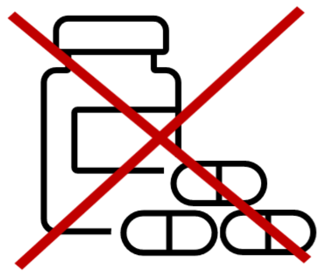
Nambala ya ma pilisi pa nthawi iliyonse imene mukumwa mankhwala	2 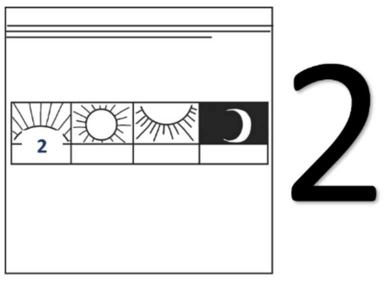	2 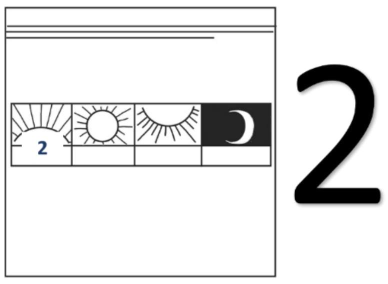	0 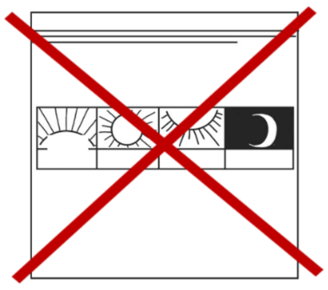
Kuchepedwe ka chiopsezo choti mukhoza kudwala chifukwa cha matenda a TB, mukamaliza kulandira thandizo la mankhwala.	65% Chiopsezo cha matenda a TB mukamaliza kumwa mankhwala chitsika kuchoka pa 10% kufika pa 4% 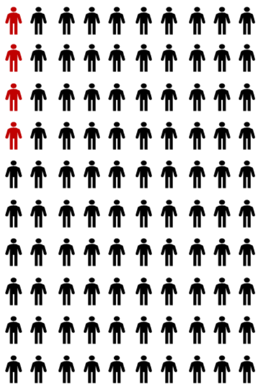	65% Chiopsezo cha matenda a TB mukamaliza kumwa mankhwala chitsika kuchoka pa 10% kufika pa 4% 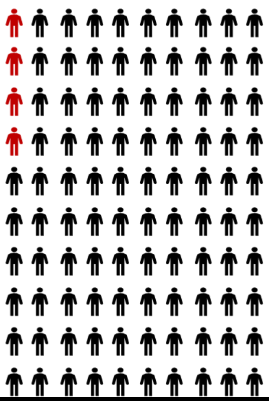	0% Chiopsezo chikhalabe pa 10% 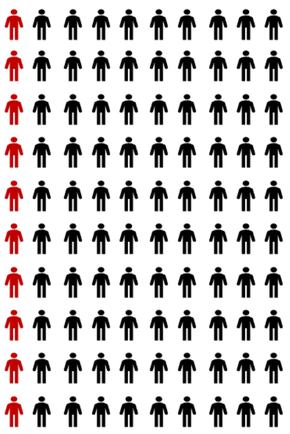
Kuona ngati mungapatsirebe ena matendawa ngakhale mutamaliza kumwa mankhwala	Kuthekera kopatsira ena TB sikuchepetsedwa 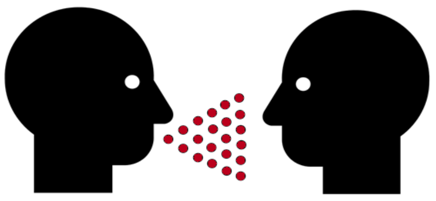	Achepetsa kuthekera koti mukhoza kupatsira ena TB ndi theka. 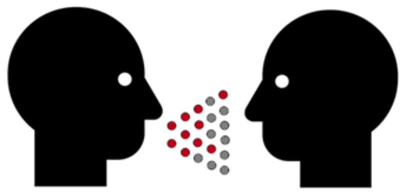	Kuthekera kopatsira ena TB sikuchepetsedwa 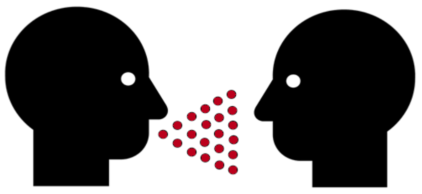
Zotsatira zosakhala bwino zimene zingabwere chifukwa cholandira mankhwala	Palibe zotsatira zosakhala bwino zobwera chifukwa chokumwa mankhwala. 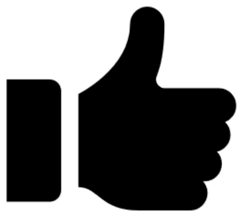	Zotsatira zosadetsa nkhawa ndipo zosazindikirika kwenikweni monga kumva ngati mukudwala kwa kanthawi kochepa 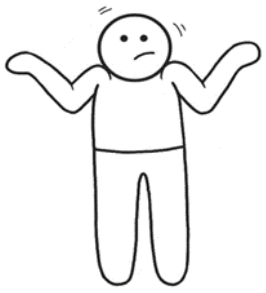	Palibe zotsatira zosakhala bwino zobwera chifukwa chokumwa mankhwala. 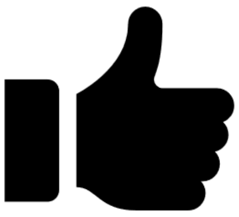
Mudzafuna kumaonedwa ndi azaumoyo mowirikiza bwanji	kamodzi (1) pa mwezi 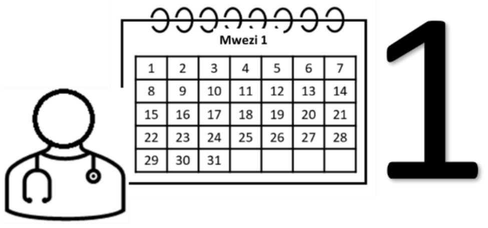	Palibe 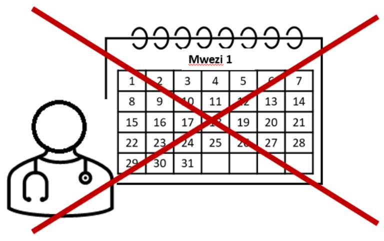	Palibe 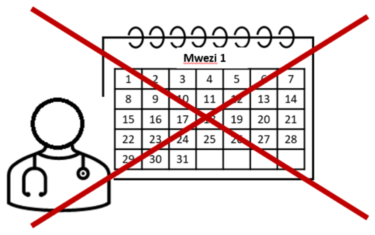
Ndalama zomwe mungagwiritse ntchito kuyenda kuchokera kunyumba kwanu kupita ku chipatala kukalandira thandizo la mankhwala pa chaka	K2400 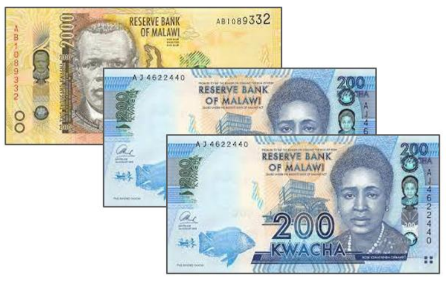	K0 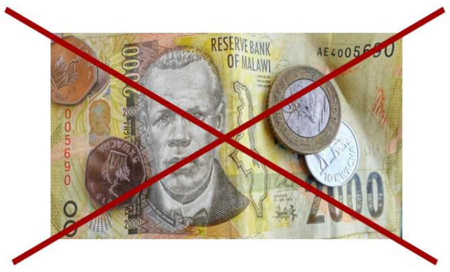	K0 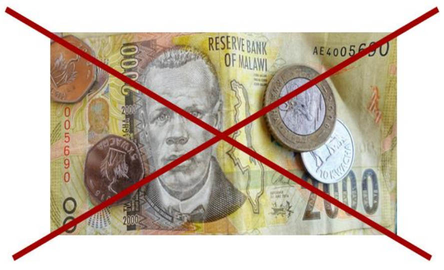
Chonde sankhani mtundu wa thandizo la mankhwala limene mulingakonde			

Scenario 11: **Ngati munayezetsa ndikupezeka ndi matenda zimene zikutanthauza kuti chiopsezo chanu chodzakhala ndi TB mu miyezi 12 ikubwerayi chili pa 10%, ndi thandizo liti la mankhwala limene mungakonde kulandira?**

	Thandizo A	Thandizo B	Palibe
Kutalika kwa nthawi yomwe mungakhale mukumwa mankhwala ngati muli pachiopsezo choti mukhoza kukhala ndi matenda a TB	Miyezi isanu (5) mukumwa mapilisi 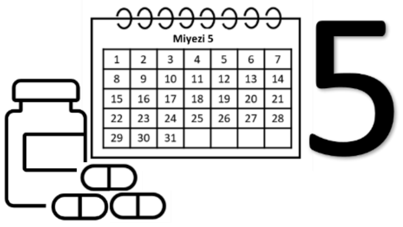	Miyezi isanu (5) mukumwa mapilisi 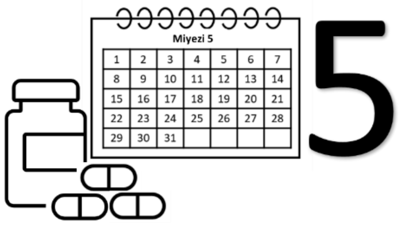	Palibe thandizo la mankhwala 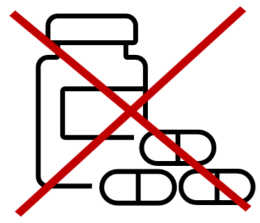
Nambala ya ma pilisi pa nthawi iliyonse imene mukumwa mankhwala	6 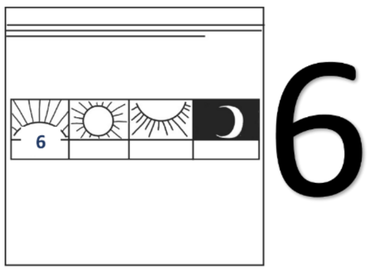	6 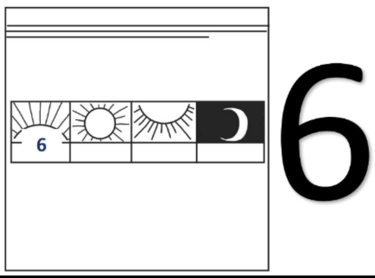	0 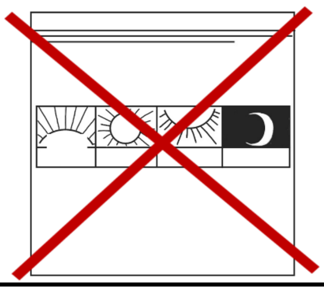
Kuchepedwe ka chiopsezo choti mukhoza kudwala chifukwa cha matenda a TB, mukamaliza kulandira thandizo la mankhwala.	95% Chiopsezo cha matenda a TB mukamaliza kumwa mankhwala chitsika kuchoka pa 10% kufika pa 1% 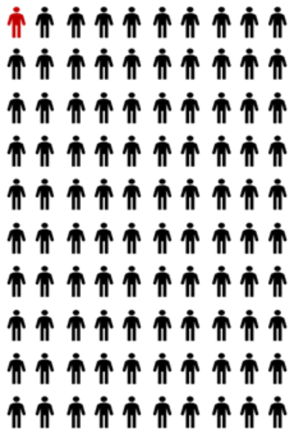	50% Chiopsezo cha matenda a TB mukamaliza kumwa mankhwala chitsika kuchoka pa 10% kufika pa 5% 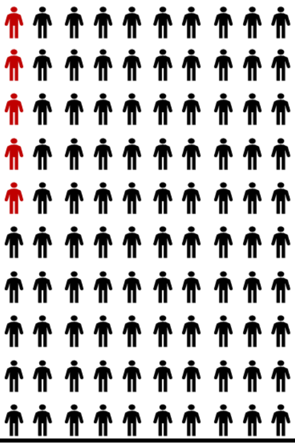	0% Chiopsezo chikhalabe pa 10% 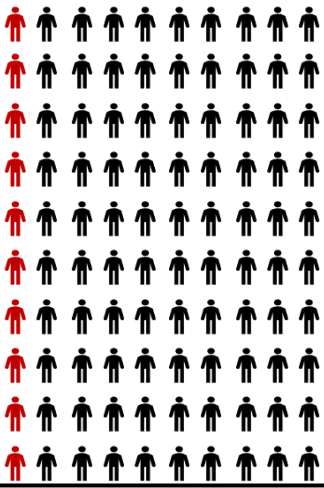
Kuona ngati mungapatsirebe ena matendawa ngakhale mutamaliza kumwa mankhwala	Kuthekera kopatsira ena TB sikuchepetsedwa 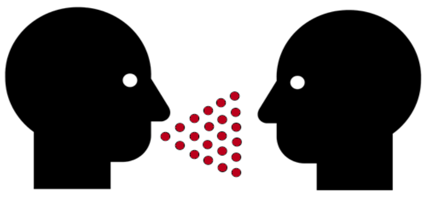	Aletseratu inu kupatsira ena TB. 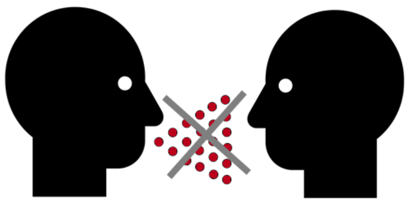	Kuthekera kopatsira ena TB sikuchepetsedwa 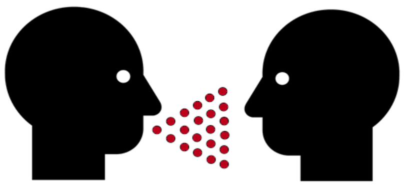
Zotsatira zosakhala bwino zimene zingabwere chifukwa cholandira mankhwala	Zotsatira zosadetsa nkhawa ndipo zosazindikirika kwenikweni monga kumva ngati mukudwala kwa kanthawi kochepa 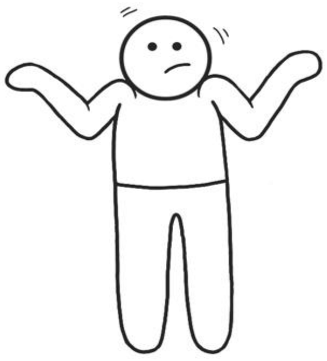	Zotsatira zocheperako mphamvu tsiku lina lililonse – zokupangitsani kusasangalala pamene muli pamodzi ndi ena koma mukutha kugwira ntchito bwinobwino 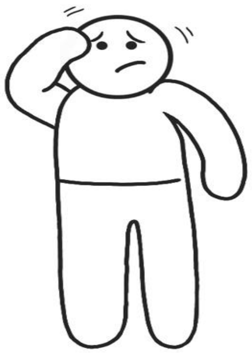	Palibe zotsatira zosakhala bwino zobwera chifukwa chokumwa mankhwala. 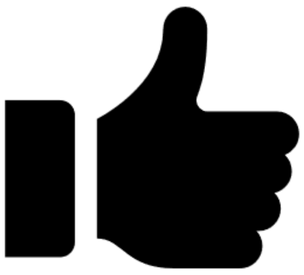
Mudzafuna kumaonedwa ndi azaumoyo mowirikiza bwanji	katatu (3) pa mwezi 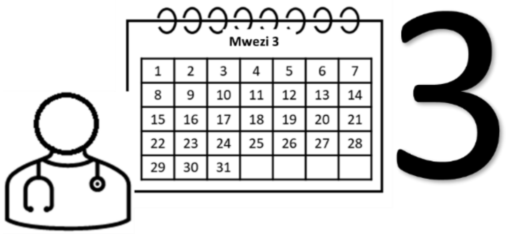	katatu (3) pa mwezi 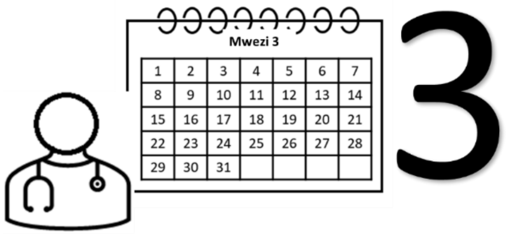	Palibe 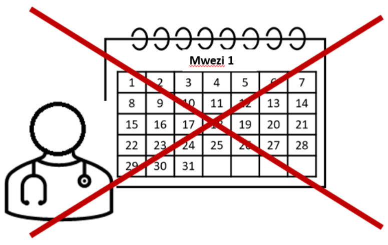
Ndalama zomwe mungagwiritse ntchito kuyenda kuchokera kunyumba kwanu kupita ku chipatala kukalandira thandizo la mankhwala pa chaka	K2,400 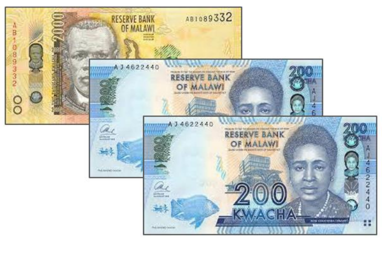	K6,000 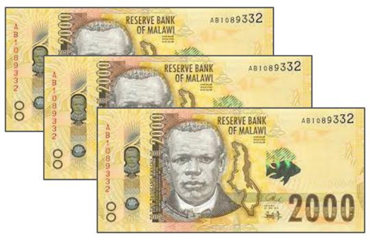	K0 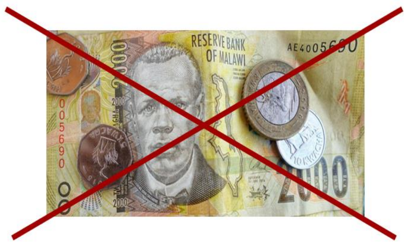
Chonde sankhani mtundu wa thandizo la mankhwala limene mulingakonde			
