# Supplementary material for: Treatment preferences among people at risk of developing tuberculosis: A discrete choice experiment
Source: PLOS Glob Public Health. 2024 Jul 19;4(7):e0002804. doi: 10.1371/journal.pgph.0002804 (PMC11259259; doi:10.1371/journal.pgph.0002804)
Supplement: S3 Appendix — (PDF) [file pgph.0002804.s008.pdf]

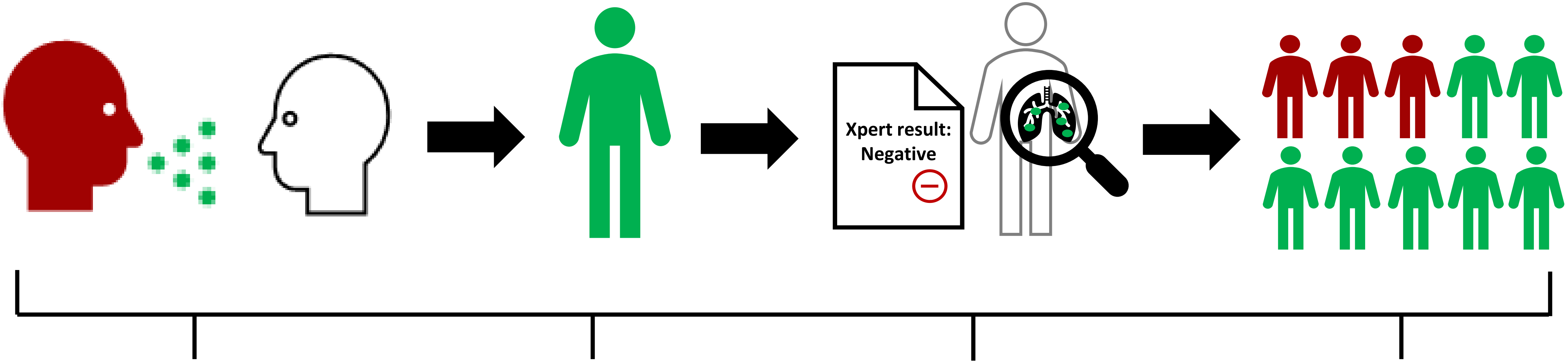

Mukakhudzana ndi munthu amene ali ndi matenda a TB, mukhoza kutenga TB.

When in contact with someone with TB disease, you can get infected with TB.

Matenda akalowa mthupi anthu ena amapezeka kuti ayamba kudwala matenda a TB **ndikukhala ndi zizindikiro monga kukhosomola ndipo akhoza kumamva kuti akudwala**

*After getting infected some people may start to develop TB disease **and have symptoms such as a cough and may feel sick.***

Mukapita ku kiliniki ndi **kukayezetsa TB pogwiritsa nthcito makhololo, nthawi zina zotsatira zanu** zimatha kutuluka zopanda matenda (**negative)** koma pounika mu chifuwa ndi X-ray, matendawa **akhoza kuoneka pa mapapo anu. Izi zikhoza kutanthauza kuti TB siinayambe mthupi mwanu pakali pano koma mukhoza kudzakhala ndi TB mtsogolo muno.**

*If you go to the clinic and take a **test for TB using sputum, sometimes** your results can come out **negative** but with a chest X-ray, the disease **can be seen on your lungs. This can mean you haven't developed TB disease now but you may develop TB disease in the future.***

Kuyeza kwa tsopano kukhoza kuthandizira **kulosera ngati mungadzakhale ndi TB** mtsogolo muno, **Koma si wina aliyense** amene zotsatira zake zasonyeza kuti ali ndi matendawa(positive) amene angadzadwale TB.

A new test can help **predict whether you will develop TB disease** in the future, **but not everyone** with a positive test will develop TB disease.
