# Supplementary material for: Treatment preferences among people at risk of developing tuberculosis: A discrete choice experiment
Source: PLOS Glob Public Health. 2024 Jul 19;4(7):e0002804. doi: 10.1371/journal.pgph.0002804 (PMC11259259; doi:10.1371/journal.pgph.0002804)
Supplement: S4 Appendix — (DOCX) [file pgph.0002804.s009.docx]

**Radio+ TB**

**In-depth Interview Guide**

| Topic  **Kuwunika mutu wa nkhani** | Questions  **Mafunso** |
| --- | --- |
| Background  **Mawu oyamba** | 1. How long have you had a cough or felt unwell?   **Kodi mwakhala mukukhosomola kapena kusamva bwino mthupi kwa nthawi yaitali bwanji?**   1. Where did you initially seek care and why?   **Munapita kuti kukapeza chisamaliro koyambilira ndipo ndi chifukwa chani?**   1. Why did you decide to come to this health facility?   **Ndi chifukwa chani munasankha kubwera pa chipatalachi?**   1. Why did you decide to seek care when you did?   **Kodi nd ichifukwa chani munapita kukapeza chithandizo mutadwala?** |
| Important factors in the diagnosis and treatment of bacteriologically negative but radiologically apparent TB among patients  **Zofunikira pa ntchito yopeza ndikupereka thandizo la mankhwala kwa odwala TB imene imayamba ndi tizirombo ta bacteria imene simaoneka wodwala akayezedwa makhololo koma imapezeka wodwala akaunikidwa mu chifuwa pogwiritsan ntchito makina ounikira mu chifuwa pakati pa odwala TB** | |
| Knowledge on TB  **Kukhala ndi nzeru zokhudzana ndi matenda a TB** | 1. What do you understand about tuberculosis (TB)?   **Kodi mmaganiza kuti TB ndi matenda anji/otani?**   1. Can you describe how people can get infected with TB or how TB spreads?   **Kodi mungafotokoze m’mene TB imafalira?**   1. What are the signs and symptoms of TB?   **Chimene chingakuzindikiritseni kuti munthu ali ndi TB ndi chani?** |
| Diagnosis of TB (general)  **Kuyeza TB (zonse pamodzi)** | 1. a. Have you ever been tested for TB or heard of others being tested for TB?   b. what do you know about how TB is diagnosed?  **Kodi munayezedwapo kapena kumvapo kuti ena ayezedwa TB?**  **Mukuziwapo chani za mmene amayezera TB kuchipatala?**   1. What do you think are some good things about how TB is diagnosed?   **chimene chimakusangalasani mukaona mmene anthu amayezedwera ndi chani?**   1. What do you not like about the way people are currently diagnosed for TB?   **chimene sichimakusangalatsani mukaona mmene anthu amayezedwera ndi chani?**   1. What aspect of how TB patients are diagnosed would you change? Why?   **Kodi chimene mungafune chitasintha mukaona mmene anthu amayezedwera pano ndi chani? Chifukwa chani?** |
| Treatment of TB (general)  **Thandizo la mankhwala a TB (zonse pamodzi)** | 1. Can you describe the current treatment for TB?   **Kodi anthu amene apezeka ndi TB amalandira thandizo lanji pano?**   1. What do you think are some good things about how people are treated for TB?   **Kodi thandizolo ndilabwino munjira zanji?**   1. What do you not like about the way people are treated for TB?   **chimene simmachiona bwino pathandizolo ndi chani?**   1. What aspect of how TB patients are treated would you change? Why?   **Ndi chani chimene mungasinthe pa thandizolo?** |
| knowledge on Incipient TB  **Maganizo pa TB imene ikuyamba kumene** | 1. Some people that have TB can go for a sputum TB test at the clinic, but the results can come out negative. But if they go for a chest x-ray the disease can be seen on their lungs. Why do you think someone can have a negative sputum TB test but positive x-ray result?   **Anthu ena amene ali ndi TB akhoza kupita kukayezetsa makhololo ku kiliniki, koma zotsatira zikhoza kusonyeza kuti laibe matenda. Koma atati apite kukayezetsa mu chifuwa pogwiritsa ntchito makina a x-ray, matendawa akhoza kuoneka mu mapapo awo. Ndichifukwa chani mukuganiza kuti munthu akhoza kuyezetsa makhololo ndikukhala osapezeka ndi TB koma zotsatira kutuluka kupezeka kuti ali ndi TB akayezetsa pogwiritsa ntchito makina a x-ray?** |
| Management of incipient TB (general)  **Chisamaliro cha TB imene ikuyamba kumene (zonse pamodzi)** | 1. How would you expect individuals that have a negative sputum test but positive chest-Xray result to be managed?   **kodi anthu amenewa angapatsidwe thandizo lanji, kapena angasamalidwe bwanji?** |
| Diagnosis preferences  **Zokonda pa kapezedwe ka matenda** | 1. If there was a test to see if these individuals were likely to get worse from TB disease and develop a positive sputum test in the future, what would the ideal diagnosis tool/strategy be like for you? Can you give examples?  - Eg: Time to results, pain.   **Ndiye patakhala kuti anthuwa ali ndi kuthekera kodzadwala kwambiri mtsogolo mpaka makhololo kuonetsa kuti ali ndi TB, mungakonde atayezedwa pogwiritsa ntchito njira yotani?**  **Mwachitsanzo: yotenga nthawi yaitali kapena yaifupi? Yosapweteka?**   1. If the test to see if they are likely to get worse from TB disease and develop a positive sputum test in the future is voluntary, what would influence their decision to take up this test?   **kutakhala kuti anthuwa ali ndi mwai wovomera kapena kukana kuyezetsa kuti aone ngati ali ndi kuthekera kodzadwala kwambiri mtsogolo mpaka makhololo awo kuonetsa kuti ali ndi TB, chingawapangitse kuti apite kukayezetsa ndi chani?**   1. What do you think about the diagnosis procedure for TB that is negative on a sputum-based test, but the disease can be detected with a chest x-ray?  - Concerns about the current procedure (no diagnosis or treatment). - Future diagnosis strategies.   **Kodi Maganizo anu ndi otani pa njira yoyezera TB imene imaonetsa kuti mulibe koma mukayezedwa ndi X-ray nkupezeka kuti muli nayo? Imakupatsani nkhawa? Fotokozani.** |
| Treatment preferences  **Zokonda pa kaperekedwe ka tandizo la mankhwala** | 1. If you were found to be positive on such a test, meaning you might get worse from TB disease and develop a positive sputum test in the future, would you be willing to be put on treatment even if it is possible that you may never get worse from TB disease? Why or why not?  - What would be the most important thing for you to consider when offered such a treatment? - What does your answer depend on? Eg: if the treatment has no side-effects, free, treatment duration, risk of disease. - Would the chance of protecting others from catching the disease from you influence your decision?   **Atati akupezani kuti muli ndikuthekera kodzadwalika chifukwa cha matenda a TB nkufika poti makhololo anu asonyeza kuti muli ndi matendawo, mungalole kuyamba kulandira chithandizo, olo mutapanda kudzadwala? Chifukwa chani?**  *- Zofunikira zimene zimene mungakonde/mungaone mutapatsidwa thandKodi ndi chinthu chiti chofunikira kwambiri chimene mungaunikire kweni-kweni mukapatsidwa mtundu wa thandizo la mankwala limeneli*  *- Kodi yankho lanu likudalalira chani? Eg: Ngati thandizo la mankhwlawo lilibe mavuto ena aliwonse, ndi la ulele, kutalika kwa nthawi mukumwa mankhwalawo, chiopsyezo cha matendawa*  *- Kodi mwayi wofuna kuteteza ena ku matendawa kuchokera kwa inu ungakulimbikitseni kupanga chiganizo?*   1. What would your ideal treatment for preventing you from getting worse with TB disease in the future look like?  - Side effects, treatment duration   **Kodi mungafune kuti mankhwala okuthandizani kuti musadzadwalike TB mtsogolomu adzakhale otani?**  **-** *Zotsatira zobwera chifukwa chodana ndi mankhwala, kutlaika kwa nthawi mukumwa mankhwalawo treatment duration*   1. What do you think about the treatment procedure for TB that is negative on a sputum test but can be detected with a chest X-ray?  - Concerns about the current procedure (no treatment) - Future treatment strategies.   **Kodi Maganizo anu ndi otani pa thandizo limene lingaperekedwe kwa anthu amene sanapezeke ndi TB pamene amayezetsa makhololo koma apezeka nayo pamene amawayeza ndi X-ray?**  **-** *Nkhawa zokhudzana ndi ndondomeko zimene zilipo pakali pano (popanda kupereka thandizo la mankhwala)*  *- Malamulo operekera thandizo la mankhwala mtsogolo muno.* |
| **Experiences**  **Zokumana nazo** | |
| Access  **Kufikirika** | 1. Do you think TB services are easily accessible?  - If you wanted to access TB care, what might make it hard for you to get it?   **Kodi mukuganiza kuti thandizo la TB limapezeka mosavuta?**  **-** *Mukafuna kupeza chisamaliro cha TB, ndi chani chimapangitsa thandizo limeneli kukhala lovuta kupeza?* |
| Trust in the healthcare system  **Chikhulupiliro pa chisamaliro cha umoyo** | 1. What are your perceptions on the healthcare system’s ability to diagnose and treat TB?   **Kodi maganizo anu ndiotani pa kuthekera kwa zipatala kupeza ndikupereka thandizo la mankhwala la TB?** |
| Diagnostic procedures in the trial  **Ndondomeko zopezera matenda mu kafukufuku woyeserayu** | 1. As a participant of this study, you underwent different diagnostic procedures, can you describe each of the diagnostic procedure that you went through?   **Ngati wotenga nawo mbali mukafukufukuyu, mwadutsa mundondomeko zoyezera matenda zosiyana-siyana, mungafotokoze ndondomeko zosiyana-siyana zoyezera matenda zimene mwadutsamo?**   1. What were your experiences when undergoing the following TB diagnostic procedures:  - the digital chest x-ray? - undergoing the face mask sampling? - undergoing the blood test? - Undergoing the sputum test?   **Kodi mwakumana ndi zotani pa nthawi imene mumadutsa mu ndondomeko zoyezetsa TB zotsatirazi:**   - **Ndondomeko yoyeza mu chifuwa pogwiritsa ntchito makina x-ray a digital?** - **Ndondomeko yotenga zoyesa kuchokera pa masiki?** - **Ndondomeko yoyeza magazi?** - **Ndondomeko yoyeza makhololo?**  1. Would you be willing to undergo these procedures to find out if you might have TB disease? Which specific procedures would you be willing to accept when offered? Why? Which specific procedures would be hard for you to accept when offered? Why?    - 1. **Kodi mungakhale okonzeka kuti ndondomekozi zizigwiritsidwa ntchito pa inu pofuna kupeza ngati muli ndi matenda a TB?**      2. **Ndi ndondomeko ziti kweni-kweni zimene mungakhale okonzeka kugwiritsa ntchito mutapatsidwa kuti musankhe? Ndi chifukwa chani?**      3. **Ndi ndondomeko ziti kweni-kweni zimene mukuona kuti zingakhale zovuta kuvomereza kugwiritsa ntchito mutapatsidwa kuti musankhe? Chifukwa chani?** |
