# Supplementary material for: Treatment preferences among people at risk of developing tuberculosis: A discrete choice experiment
Source: PLOS Glob Public Health. 2024 Jul 19;4(7):e0002804. doi: 10.1371/journal.pgph.0002804 (PMC11259259; doi:10.1371/journal.pgph.0002804)
Supplement: S5 Appendix — (DOCX) [file pgph.0002804.s010.docx]

**RADIO+ TB DCE**

**Cognitive Interview Guide**

Opening statements:

Thank you for taking time to speak with me, my name is _____. We have asked you to participate in this exercise because we are studying patients’ preferences for the management of TB disease that cannot be detected with a standard sputum test. However, the disease can be detected with a chest x-ray. We are aiming to develop a clear questionnaire that can allow us to see what aspects of TB diagnosis and management patients consider important. As you go through the process, we will ask you questions about your understanding of the questionnaire. This process is likely to take about one hour to complete. Feel free to let me know if you need a break at any time. You can also stop the interview if you do not want to continue the discussion. Before we begin do you have any questions?

**Mawu Oyamba:**

**Zikomo chifukwa chotenga nthawi kulankhulana nane, dzina langa ndi _____. Takupemphani kuti mutenge nawo mbali muchochitika ichi chifukwa chakuti tikuchita kafukufuku pa zokonda zanu pa m’me mungasamalire matenda a TB amene sangathe kupezeka poyeza makhololo. Komabe, matendawa akhoza kupezeka pounika mu chifuwa pogwiritsa ntchito makina ounikira a x-ray. Tili ndi cholinga chofuna kukonza mafunso omveka bwino, amene angathe kutilola kuwona kuti ndi magawo ati akapezedwe ka matenda a TB komanso kasamalidwe kake, amene anthu amawatenga kukhala ofunikira. Pamene muli mkati-kati mwa ndondomekoyi, tidzakufunsani mafunso okhudzana ndi kumvetsa kwanu kwa mafunsowa. Ndondomekoyi itenga pafupi-fupi ola limodzi kuti ithe. Khalani omasuka kundidziwitsa kuti mupumule, nthawi ina iliyonse. Mukhozanso kuimitsa mchezowu mukafuna kuti usapitilire. Tisanayambe china chilichonse, muli ndi mafunso ena aliwonse?**

| 1. **General** | 1. What do you think of the layout of the questionnaire?   **Kodi mukuganizapo bwanji pa m’ndandanda wa mafunsowa?**  **Funsisitsani: Kodi mukumvetsa kuti muyambire pati komanso kuti musankhe chani?**  Probe: Do you understand where to start and which order of the choices to  follow?  **Funsisitsani: Kodi mukumvetsa kuti muyambire pati komanso kuti musankhe chani?**  How can we make the layout of the questionnaire easier to understand and follow?  **Kodi tingakonze bwanji mafunsowa kuti akhale omveka komanso otsatirika bwino?** |
| --- | --- |
|  | 1. What is your understanding of the instructions at the beginning of the questionnaire?   **Kodi mukumvetsa bwanji malangizo amene ali poyambilira pa mafunso?**  Probe: Do you understand what this questionnaire is for?  **Funsisitsani: Kodi mukumvetsa cholinga cha mafunsowa?**  Do you understand how to complete the questionnaire?  **Kodi mukumvetsa m’mene mungayankhire mafunsowa?**  How can we improve the instructions to make it easy for you to understand and follow?  **Kodi tingakonze bwanji malangizowa mwatsopano, kuti kukhale kosavuta kuwamvetsa komanso kuwatsatira?** |
| 1. **Heading (background scenario)** | 1. What is your understanding of the scenario given at the beginning of each choice set?   **Kodi mukumvetsa bwanji ntchito imene yaperekedwa kumayambiliro a gawo la yankho lina lililonse?**  Probe: Do you understand what we mean by “risk of developing TB disease?  **Funsisitsani: kodi mukumvetsa zimene tikutanthauza tikanena kuti “chiopsyezo chokhala ndi matenda a TB?**  How can we reword it to make it easier to understand?  **Kodi mawu amenewa tingawalembenso bwanji kuti akhale osavuta kumvetsa?** |
|  | 1. What is your understanding of the diagrams given with the scenario?   **Kodi mukumvetsa bwanji zithunzi zimene zaperekedwa pa chochitika china chilichonse?**  Probe: Does it help you understand what the risk of developing TB disease means?  **Funsisitsani: Kodi zikukuthandizirani kumvetsa “chiopsyezo chokhala ndi matenda a TB”?**  Do the diagrams simplify your understanding of what the risk of developing TB disease means?  **Kodi zithunzizi zikuphweketsa kumvetsa kwanu za tanthauzo la chiopsyezo chokhala ndi matenda a TB?**  How can we modify the diagrams to make it easier to understand?  **Kodi tingasinthe bwanji zithunzizi kuti zikhale zosavuta kumvetsa?** |
| 1. **Instruction before each choice set** | 1. Please tell me in your own words what the instruction before this choice set is telling you to do?   **Chonde ndifotokozereni momveka bwino zimene malangizo akufotokoza kuti muchite tisanafike pa gawo la yankho lina lililonse limene laperekedwa?**  Probe: How clear is the instruction? How can we improve it to make it easier to understand?  **Funsisitsani: Kodi malangizowo akumveka bwino bwanji? Kodi tingawakonze bwanji mwatsopano kuti akhale osavuta kumvetsa?** |
|  | 1. Please go ahead and carry out the instruction as you understand it, please think out loud while you are completing the task. [Record observations]   **Chonde pitilizani kutsatira malangizo m’mene mukuwamvetsera, Chonde lankhulani zimene mukuganiza pemene mukuchita ntchito imeneyi. [Lembani zimene mwaona]** |
|  | 1. How easy or difficult was it to complete the task?   **Kodi kunali kophweka kapena kovuta bwanji kuti mumalizitse ntchitoyi?**  Probe: If there were any problems, how could these be resolved?  **Funsisitsani: Ngati panali mavuto ena aliwonse, kodi mavutowo angakonzedwe bwanji?** |
| 1. **Attributes and levels** | **NB: To be done for each attribute**   1. What do you understand this attribute to mean?   **Kodi mukumvetsa kuti maonekedwewa akutanthauza chani?**  *Probe: Is the wording of the attribute clear?*  ***Kodi mawu oti maonekedwe akumveka bwino?***   1. How can we make the wording of the attribute easier to understand?   **Kodi mawu onena “*kuchuluka kwa nthawi imene mungakhale nayo kuti mulandire mankhwala mutakhala pa chiopsyezo chokhala ndi matenda a TB”*, tingawalembe bwanji kuti akhale osavuta kumvetsa?**   1. What is your understanding of each of the attributes levels?   **Kodi mukumvetsa bwanji gawo lina lililonse la mlingo wa maonekedwewa?**  *Probe: Is the wording and layout clear?*  ***Funsisitsani: Kodi kalembedwe ndi m’ndandanda wake zikumveka bwino?***  How can we make it easier to understand?  **Kodi tingapange bwanji kuti zimveke bwino?**   1. Do the pictures help you understand what this attribute is?   **Kodi zithunzizi zikukuthandizirani kumvetsa chimene maonekedwewa ali?**  *Probe: can you describe what the pictures show? Are the pictures clear?*  ***Funsisitsani: Kodi mungafotokoze chimene zithunzizi zikuonetsa? Kodi zithunzizi zikuoneka bwino-bwino?***   1. How can we make the pictures for the attribute and its levels easier to understand?   **Kodi tingapange bwanji kuti zithunzi pa maonekedwe ndi milingo yake zikhale zosavuta kumvetsa?** |
